# Supplementary material for: Irradiation-induced palladium-catalyzed decarboxylative desaturation enabled by a dual ligand system
Source: Nat Commun. 2018 Dec 6;9:5215. doi: 10.1038/s41467-018-07694-w (PMC6283874; doi:10.1038/s41467-018-07694-w)
Supplement: Supplementary file 1 — Supplementary Information [file 41467_2018_7694_MOESM1_ESM.pdf]

Irradiation-Induced Palladium-Catalyzed Decarboxylative Desaturation Enabled by a Dual Ligand System

Cheng *et al.*

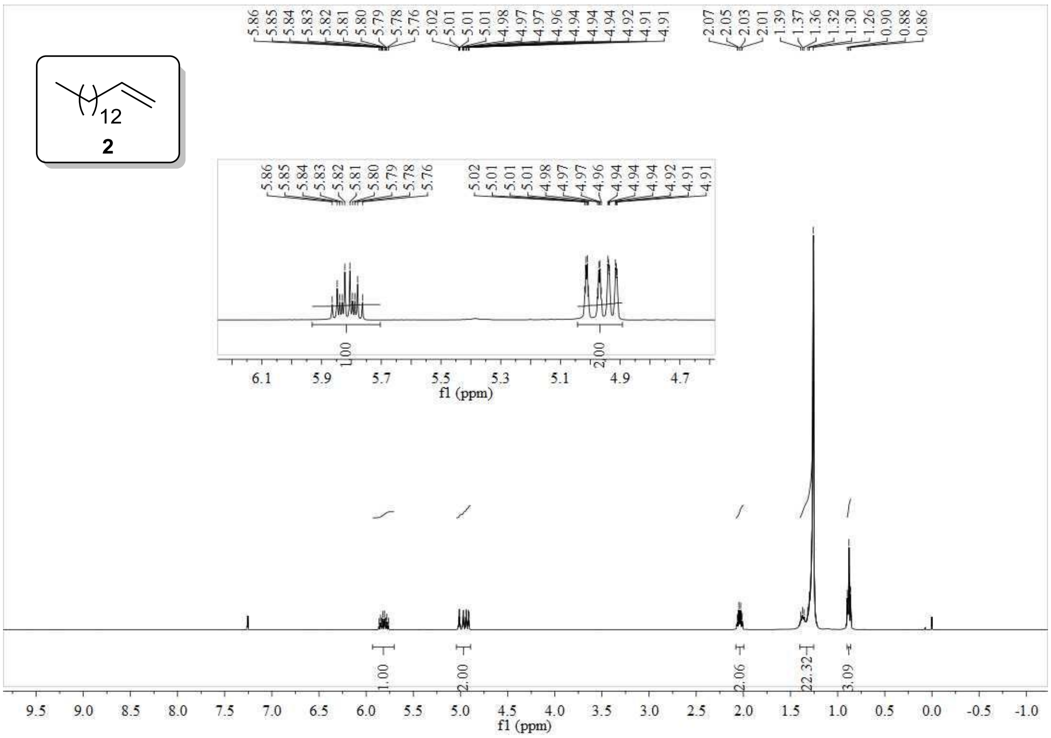

Supplementary Figure 1. ^{1}H NMR spectrum of 1-pentadecene (2)

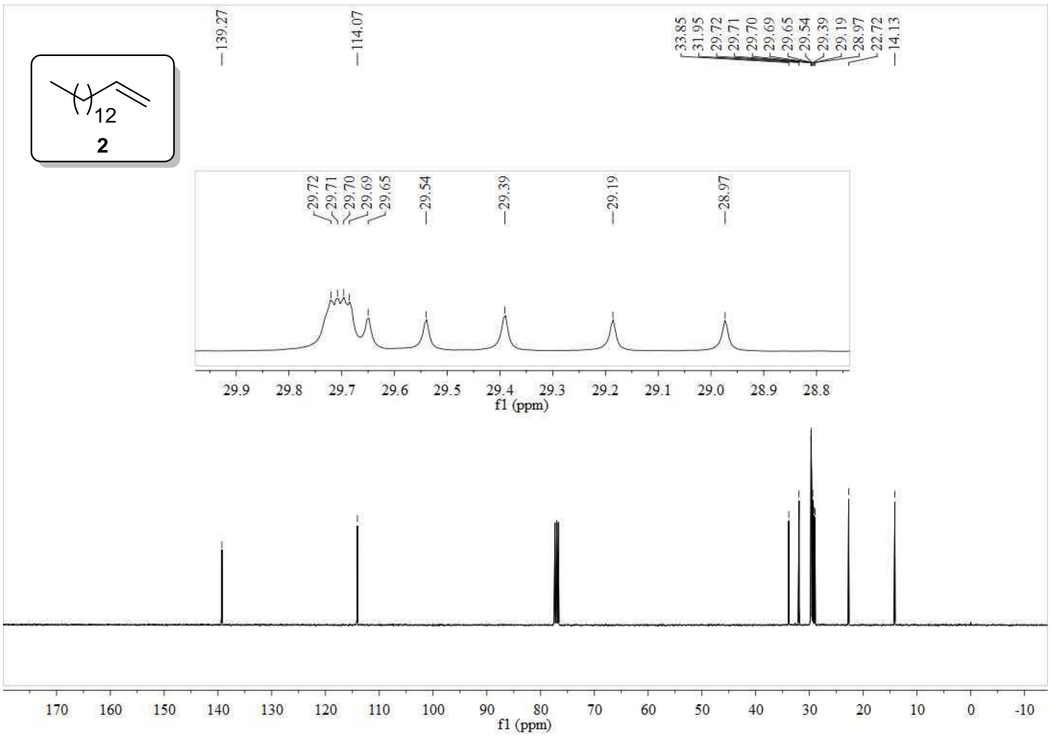

Supplementary Figure 2. ^{13}C NMR spectrum of 1-pentadecene (2)

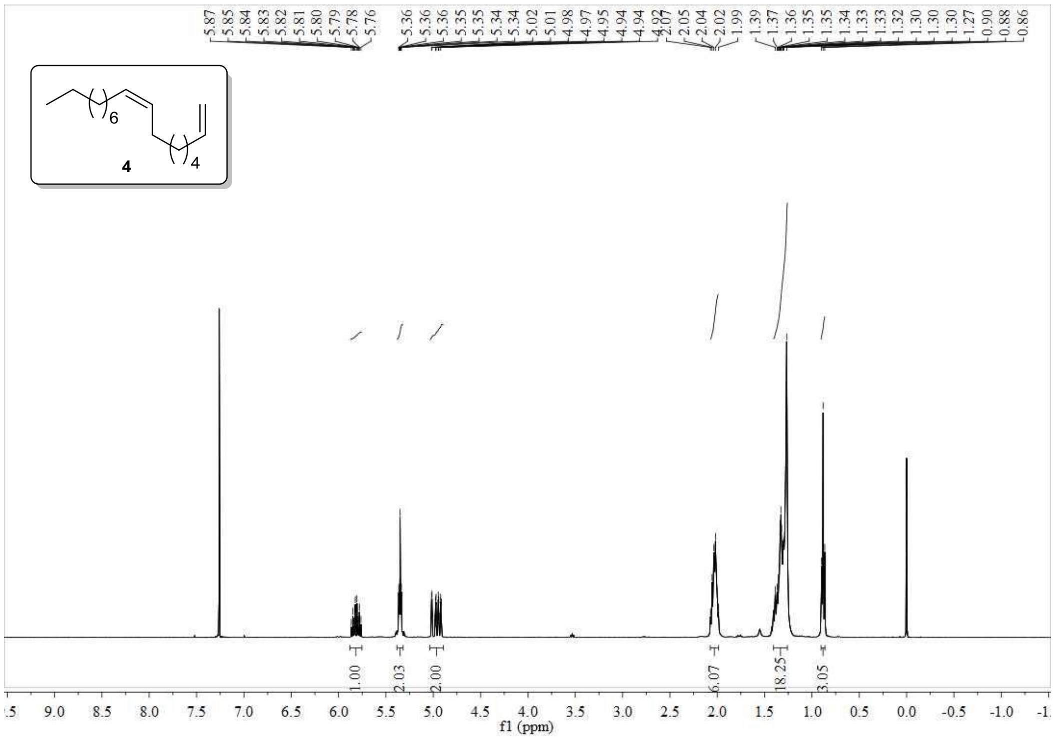

Supplementary Figure 3. ^{1}H NMR spectrum of (*Z*)-heptadeca-1,8-diene (4)

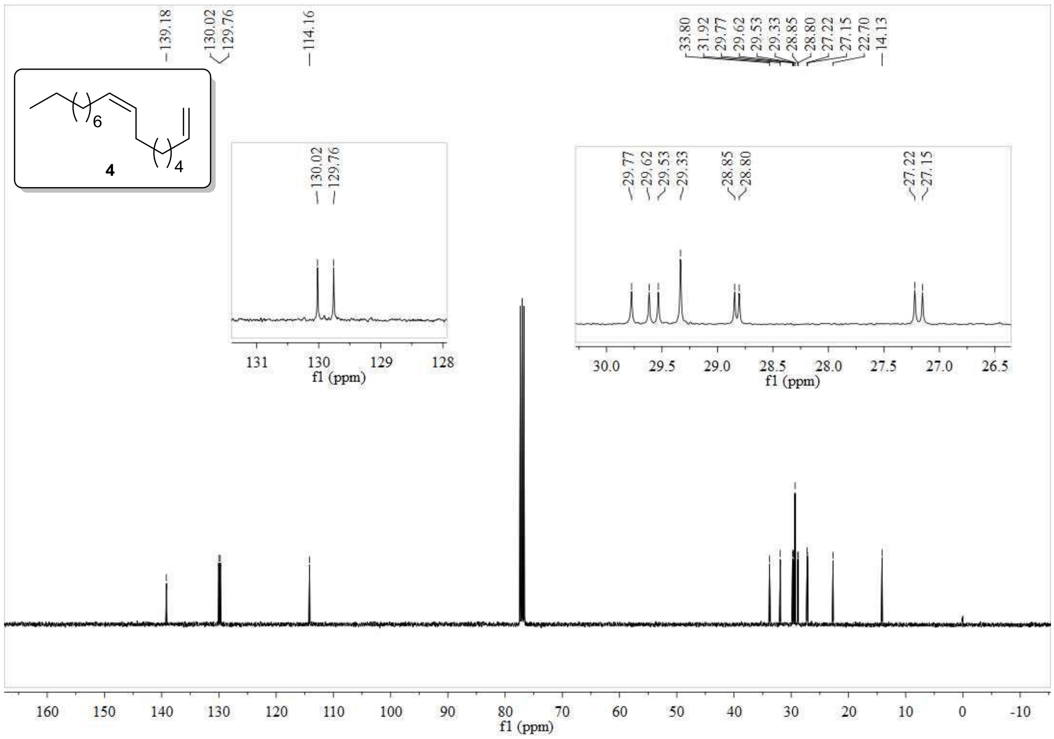

Supplementary Figure 4. ^{13}C NMR spectrum of (*Z*)-heptadeca-1,8-diene (4)

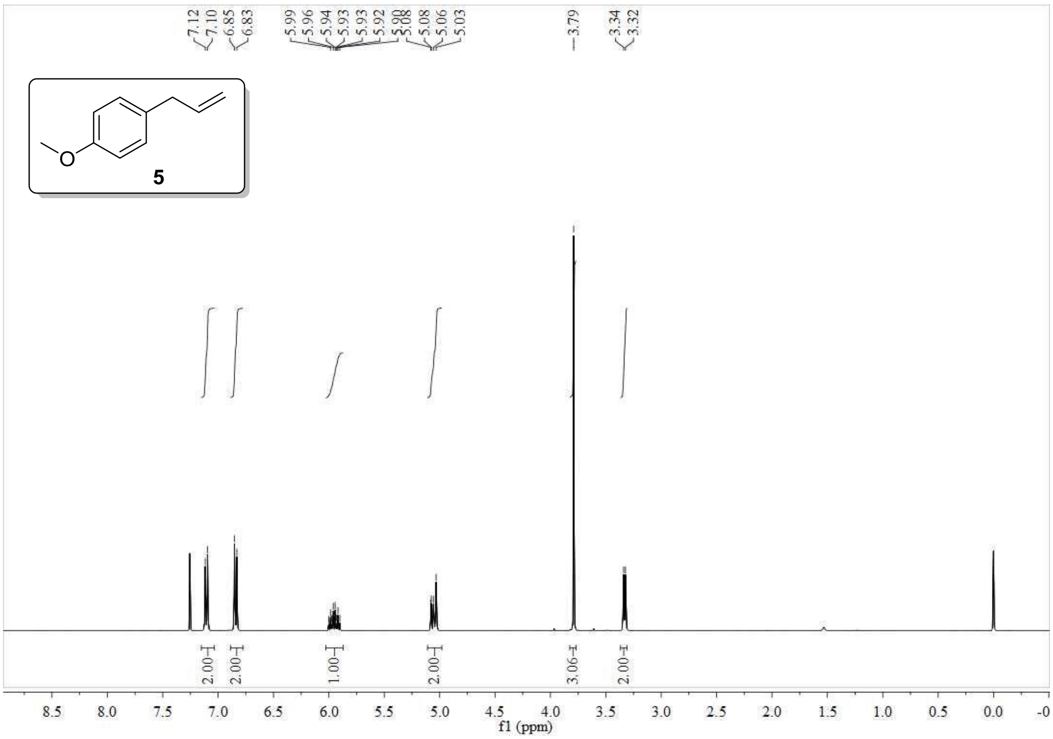

Supplementary Figure 5. ^{1}H NMR spectrum of 1-allyl-4-methoxybenzene (5)

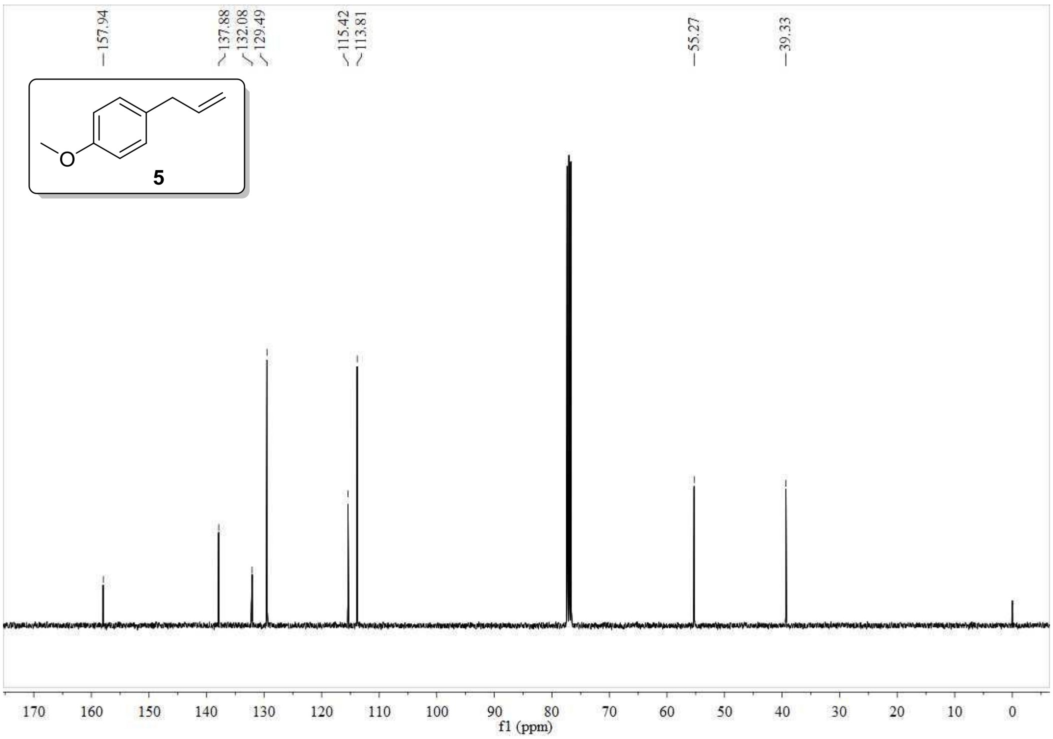

Supplementary Figure 6. ^{13}C NMR spectrum of 1-allyl-4-methoxybenzene (5)

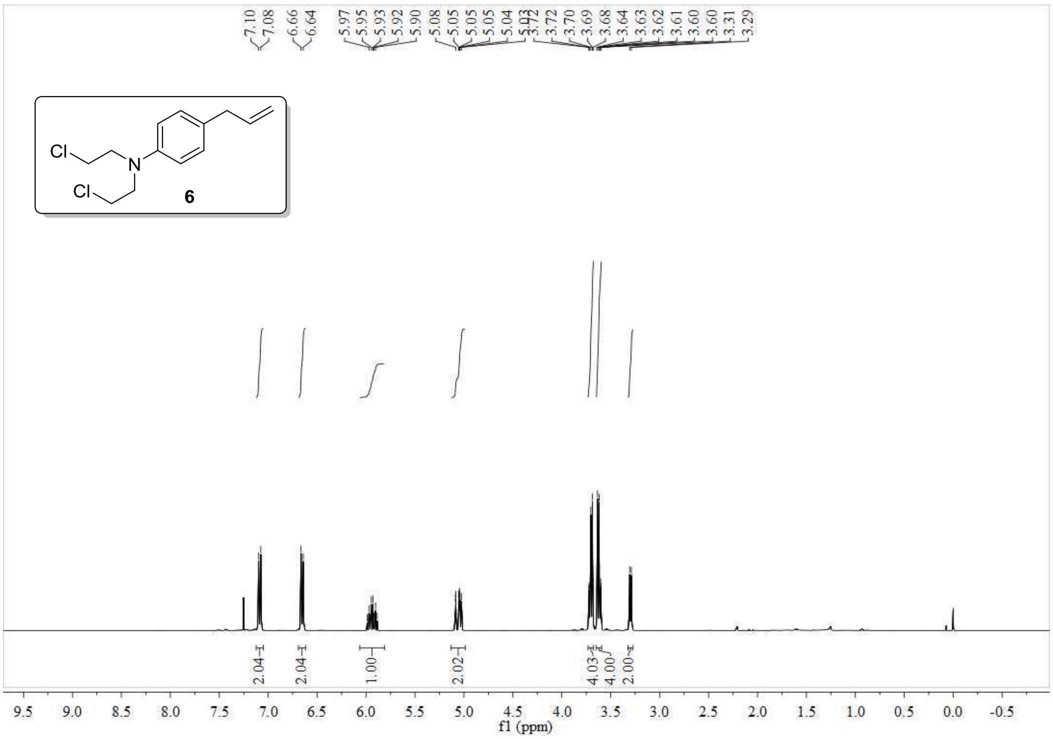

Supplementary Figure 7. ^{1}H NMR spectrum of 4-allyl-*N*,*N*-bis(2-chloroethyl)aniline (6)

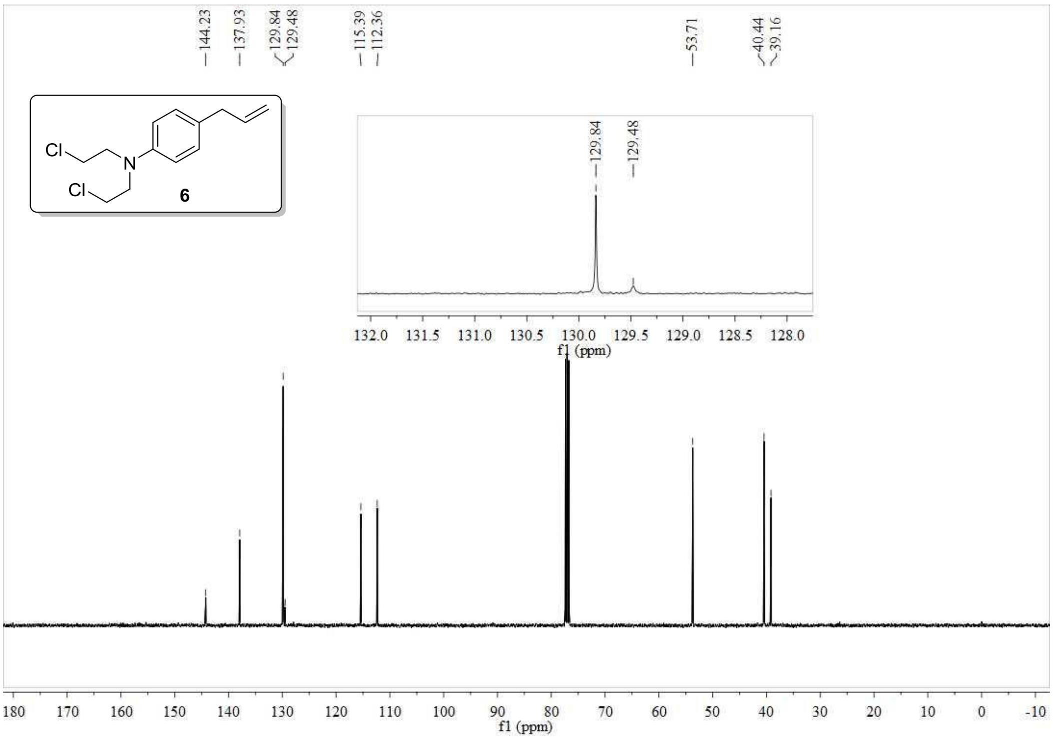

Supplementary Figure 8. ^{13}C NMR spectrum of 4-allyl-*N*,*N*-bis(2-chloroethyl)aniline (6)

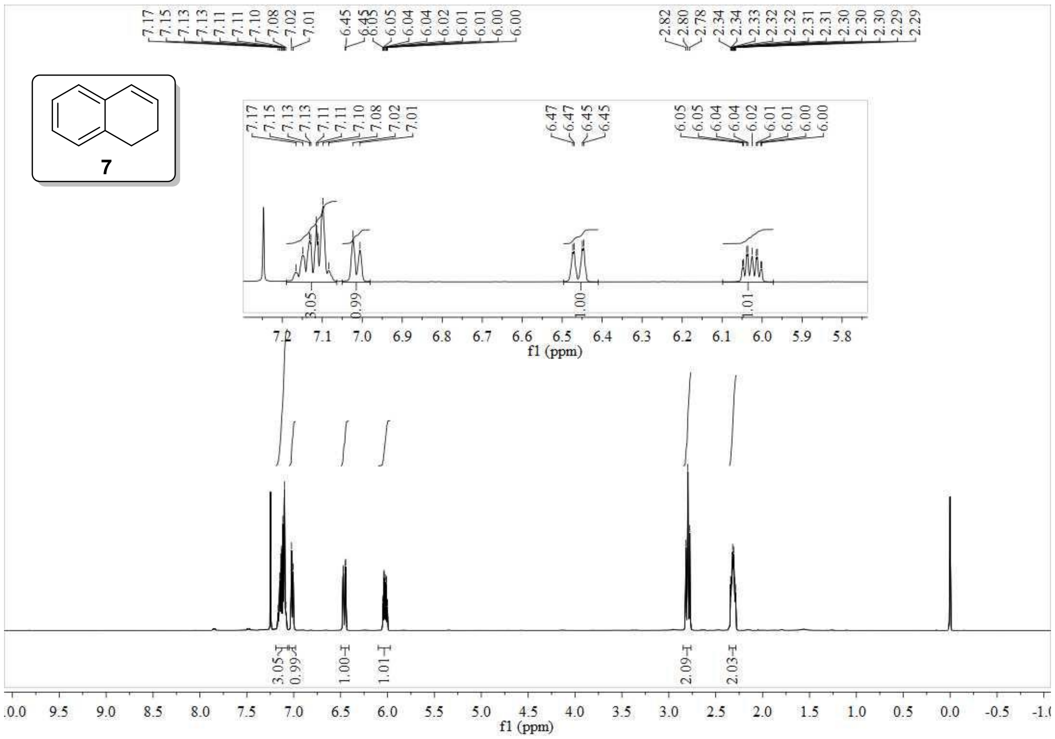

Supplementary Figure 9. ^{1}H NMR spectrum of 1,2-dihydronaphthalene (7)

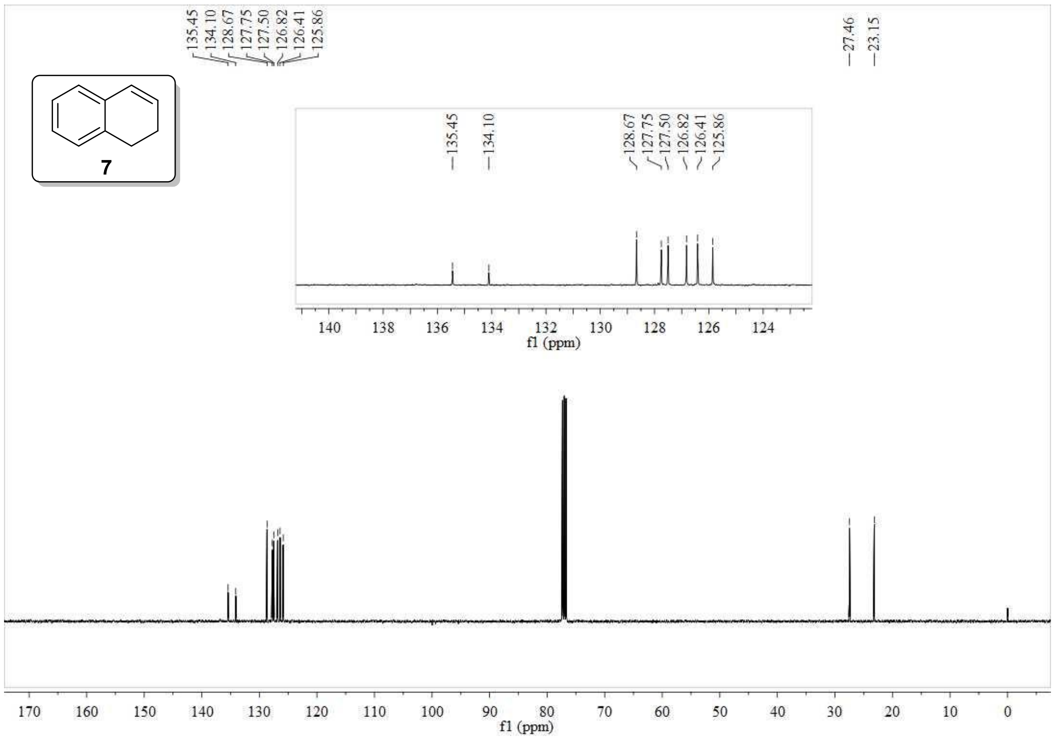

Supplementary Figure 10. ^{13}C NMR spectrum of 1,2-dihydronaphthalene (7)

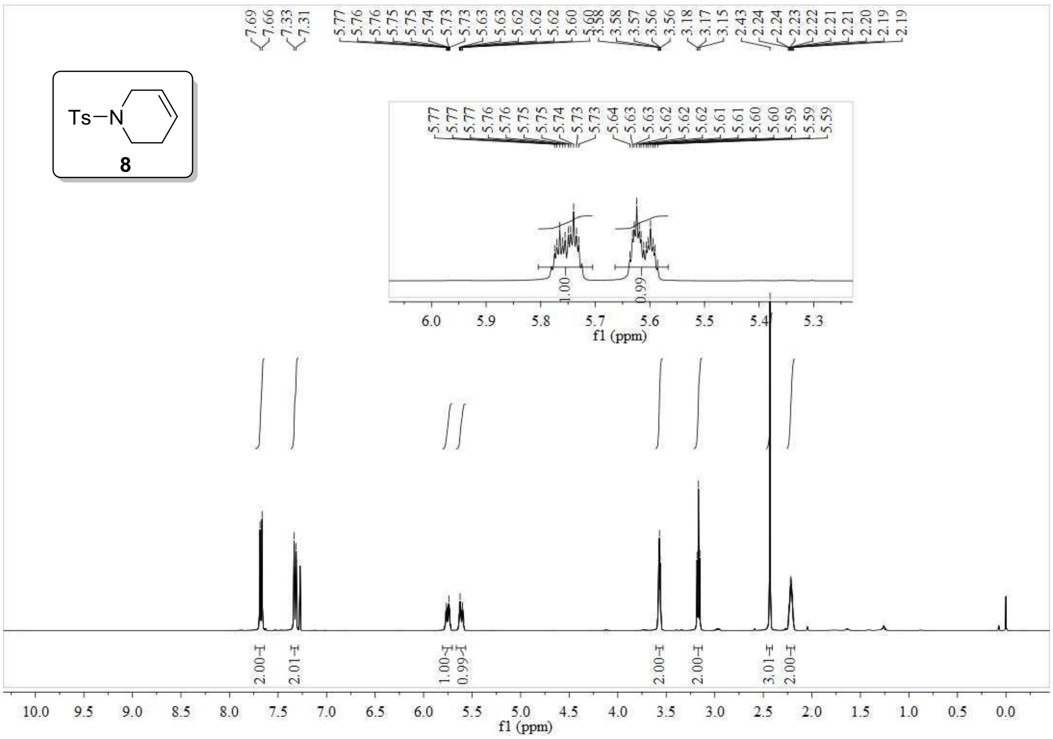

Supplementary Figure 11. ^{1}**H NMR spectrum of 1-tosyl-1,2,3,6-tetrahydropyridine (8)**

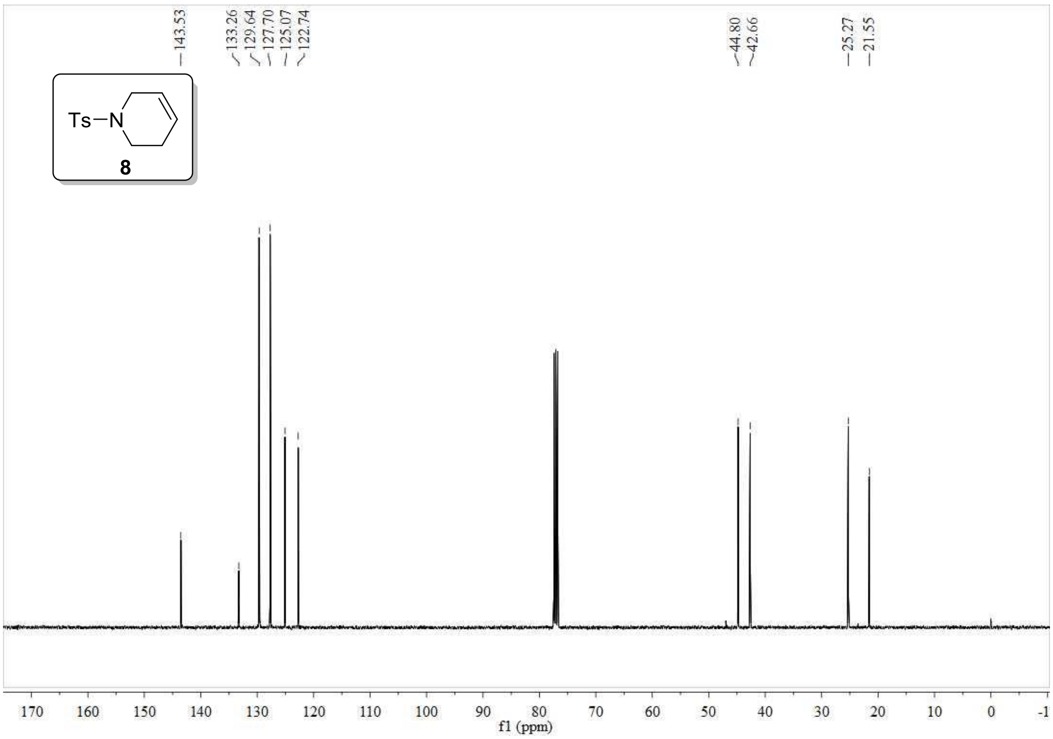

Supplementary Figure 12. ^{13}**C NMR spectrum of 1-tosyl-1,2,3,6-tetrahydropyridine (8)**

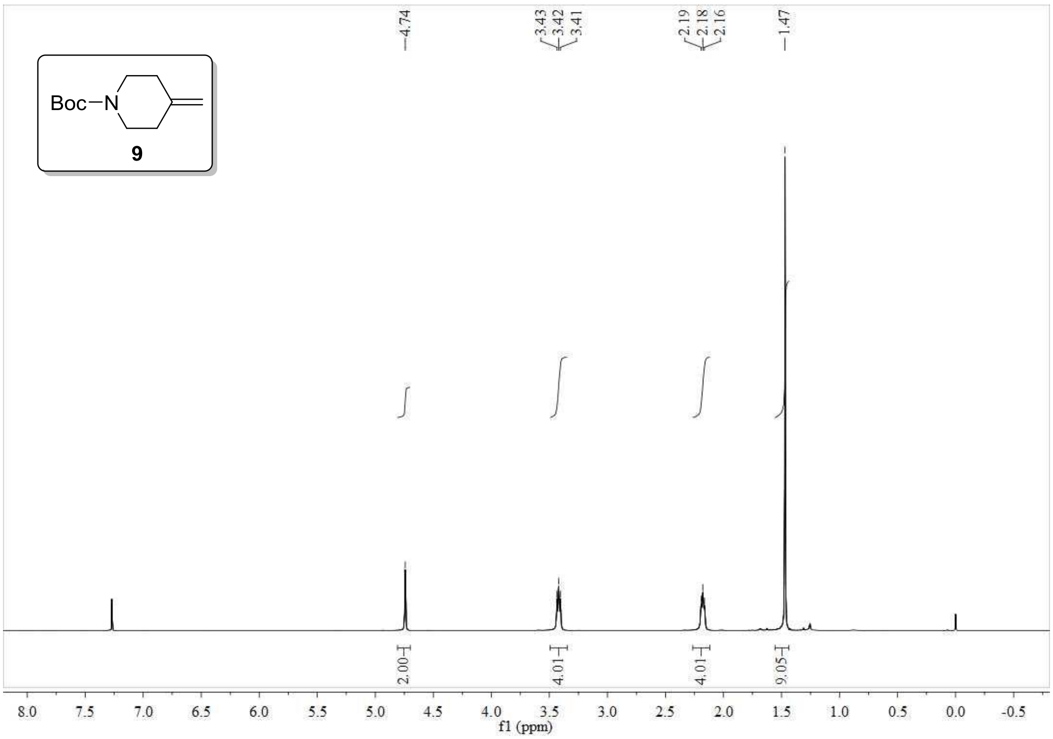

Supplementary Figure 13. ^{1}H NMR spectrum of product 9

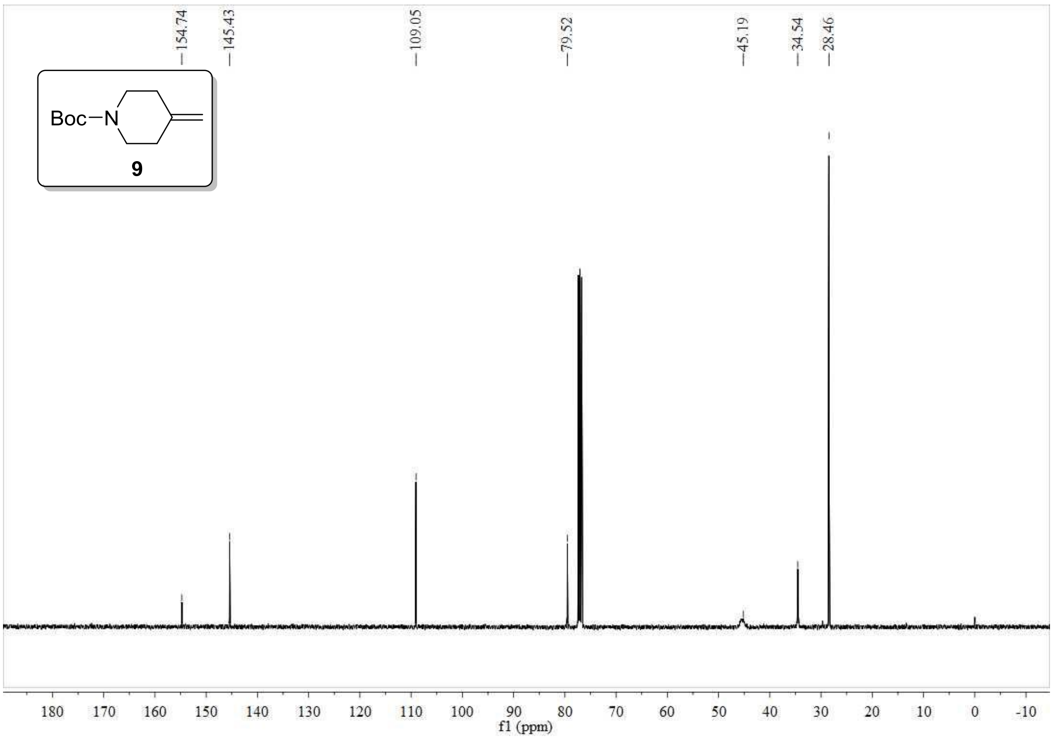

Supplementary Figure 14. ^{13}C NMR spectrum of product 9

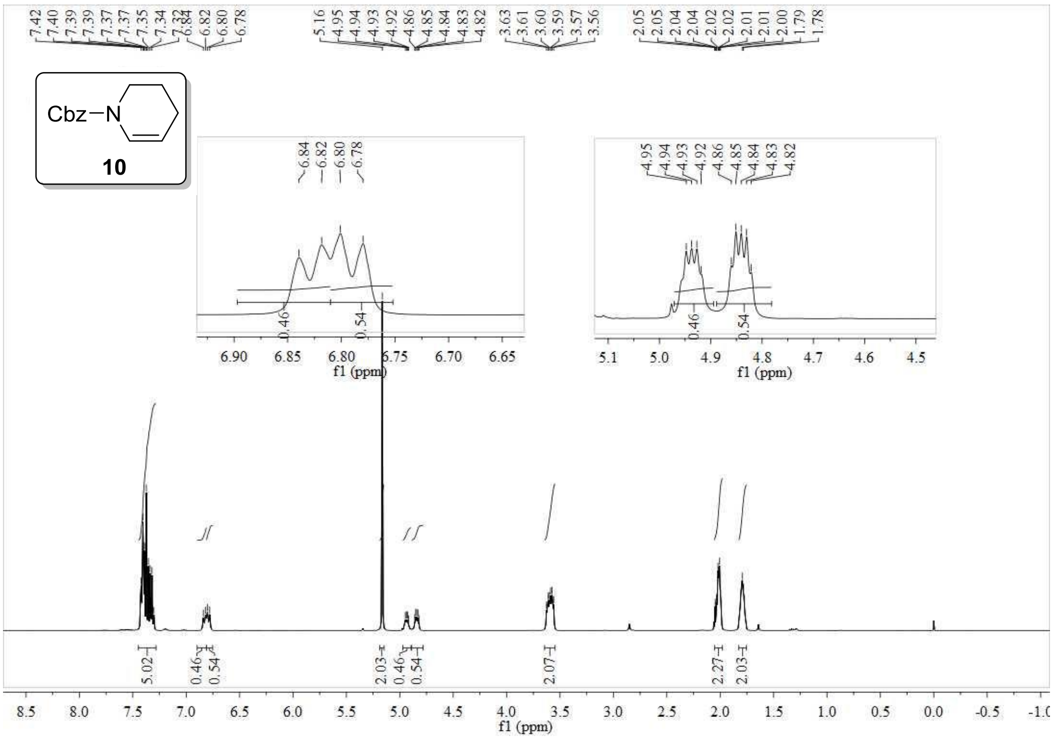

Supplementary Figure 15. ^{1}H NMR spectrum of product 10

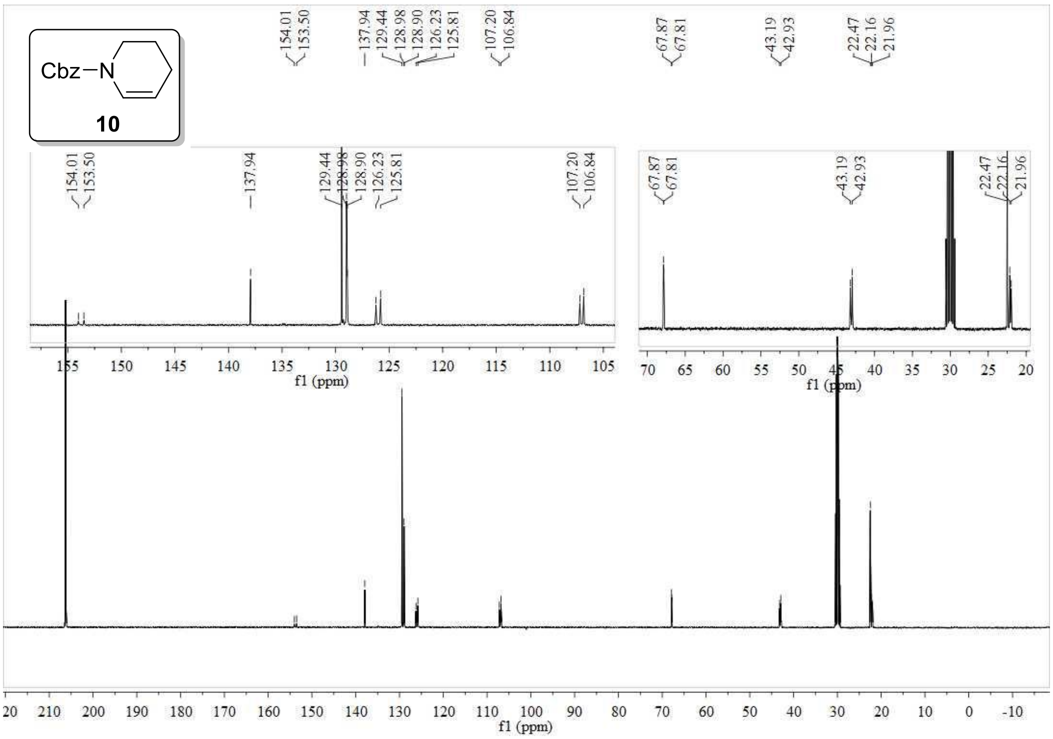

Supplementary Figure 16. ^{13}C NMR spectrum of product 10

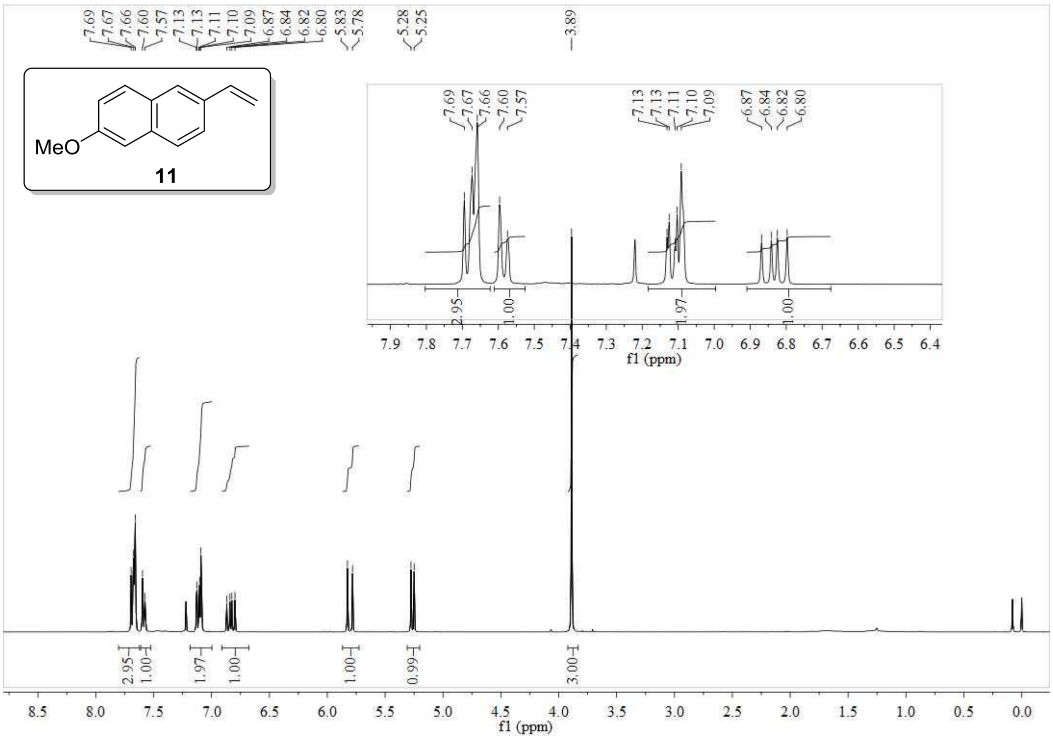

Supplementary Figure 17. ^{1}H NMR spectrum of 2-methoxy-6-vinylnaphthalene (11)

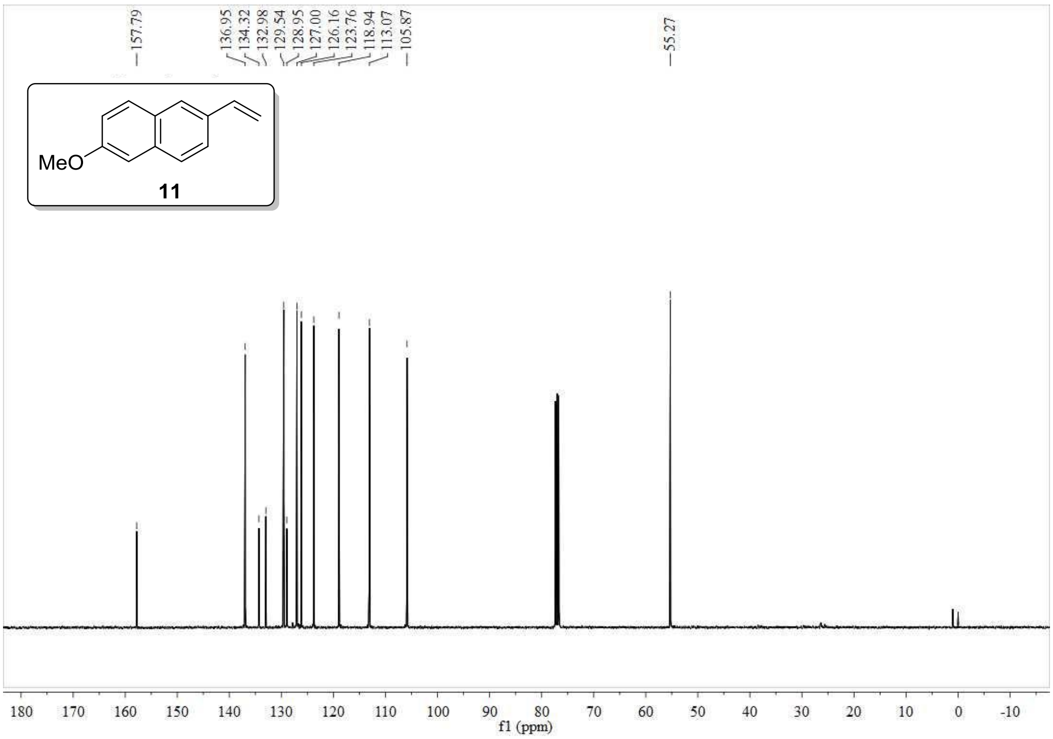

Supplementary Figure 18. ^{13}C NMR spectrum of 2-methoxy-6-vinylnaphthalene (11)

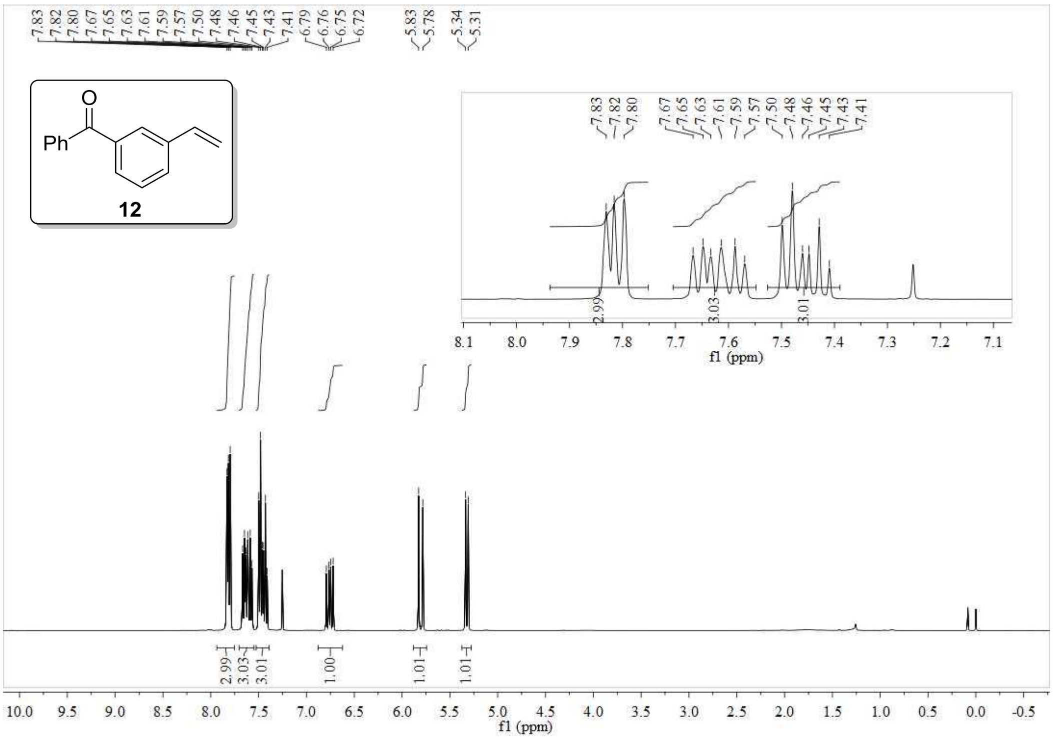

Supplementary Figure 19. ^{1}H NMR spectrum of phenyl(3-vinylphenyl)methanone (12)

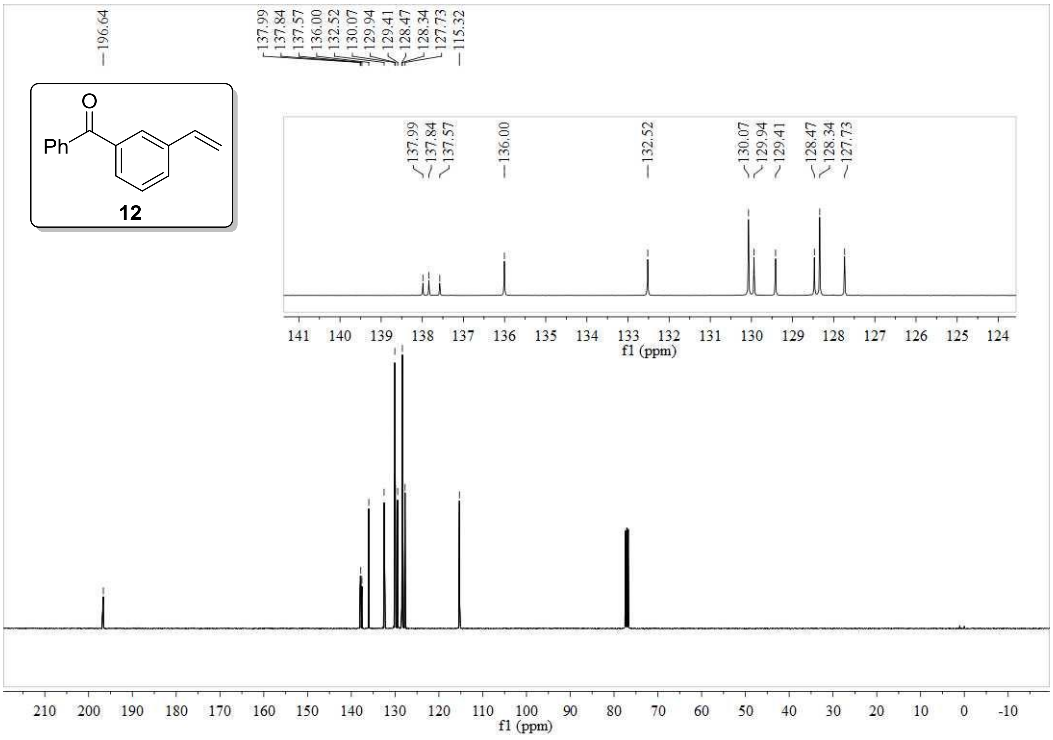

Supplementary Figure 20. ^{13}C NMR spectrum of phenyl(3-vinylphenyl)methanone (12)

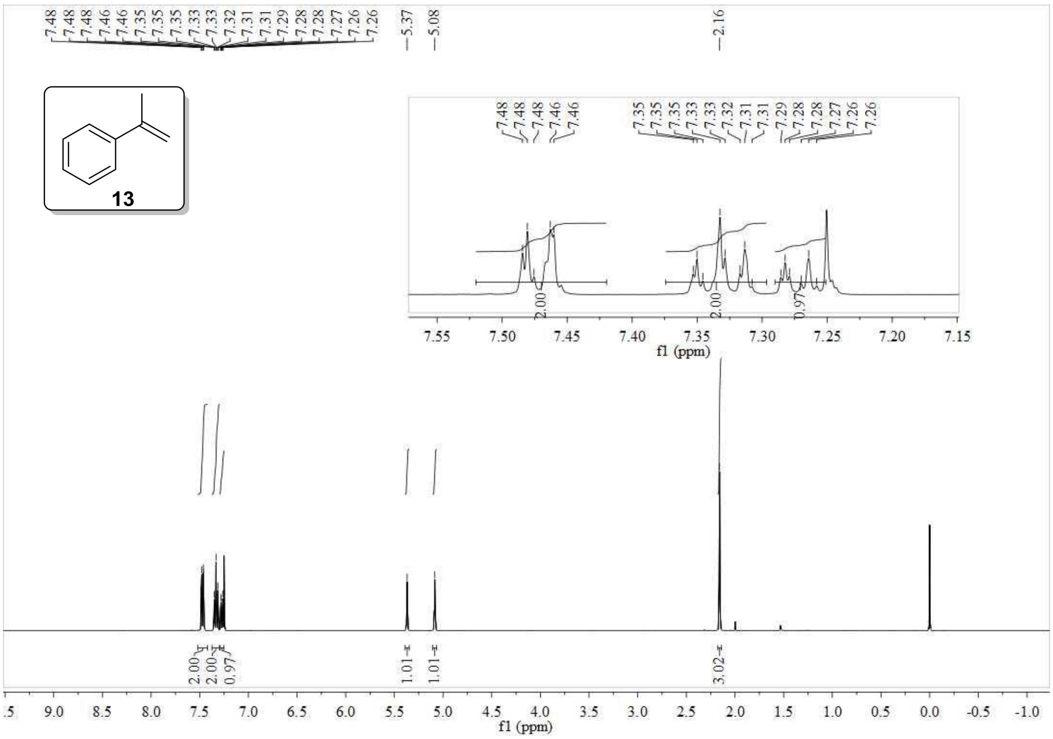

Supplementary Figure 21. ^{1}H NMR spectrum of prop-1-en-2-ylbenzene (13)

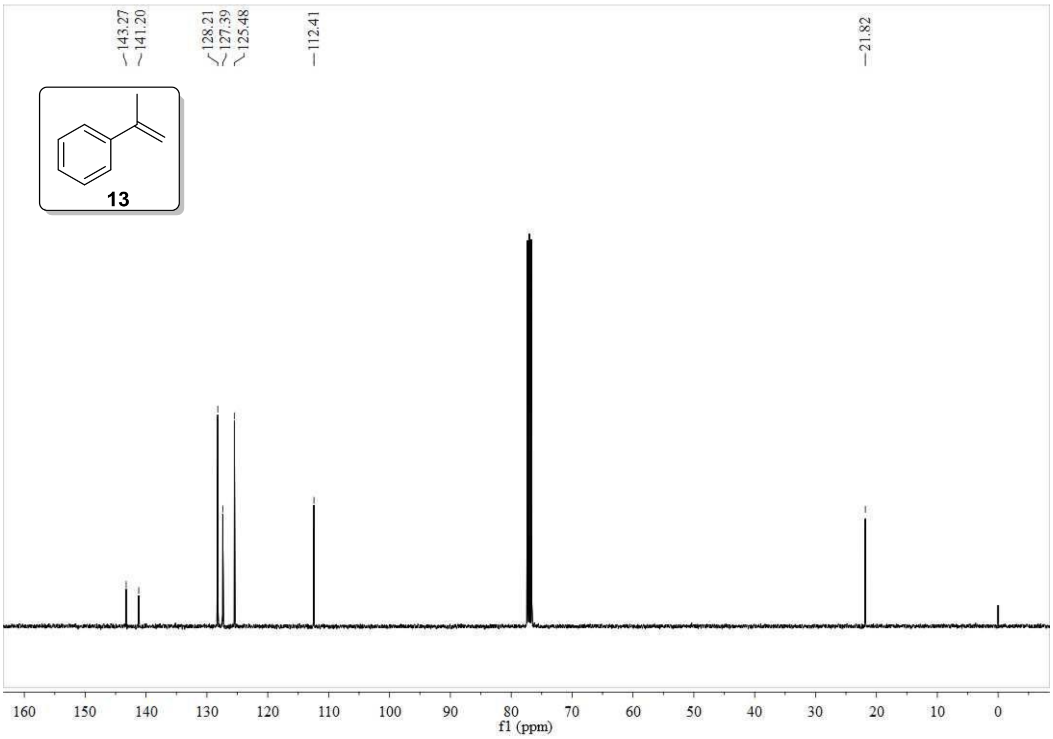

Supplementary Figure 22. ^{13}C NMR spectrum of prop-1-en-2-ylbenzene (13)

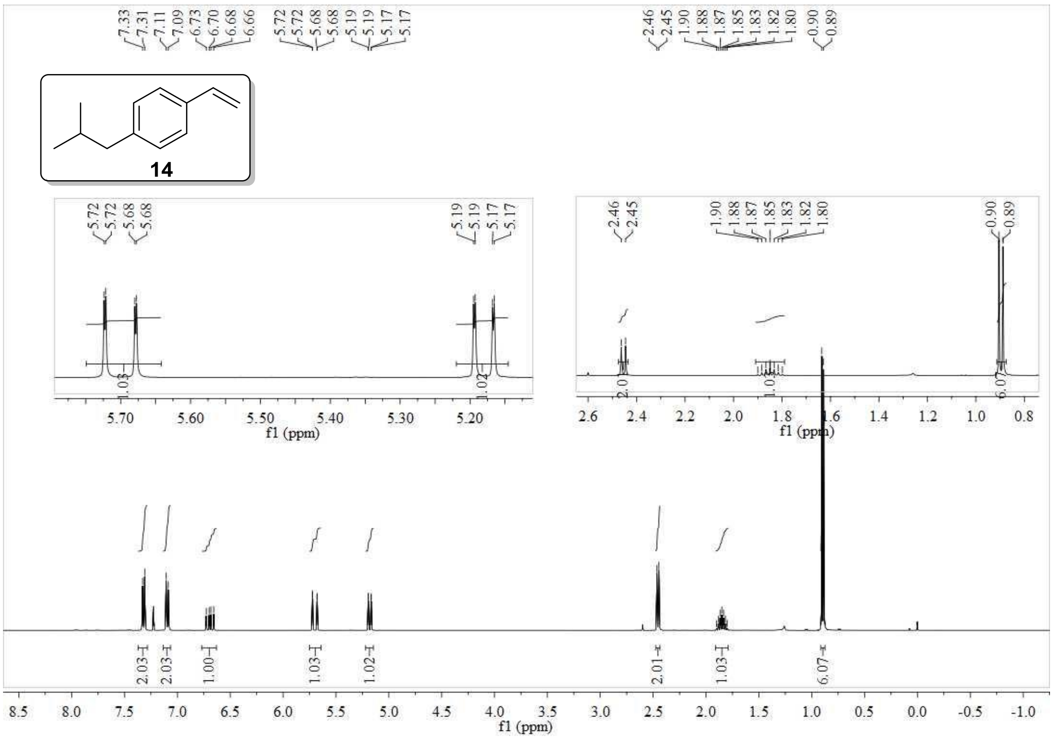

Supplementary Figure 23. ^{1}H NMR spectrum of 1-isobutyl-4-vinylbenzene (14)

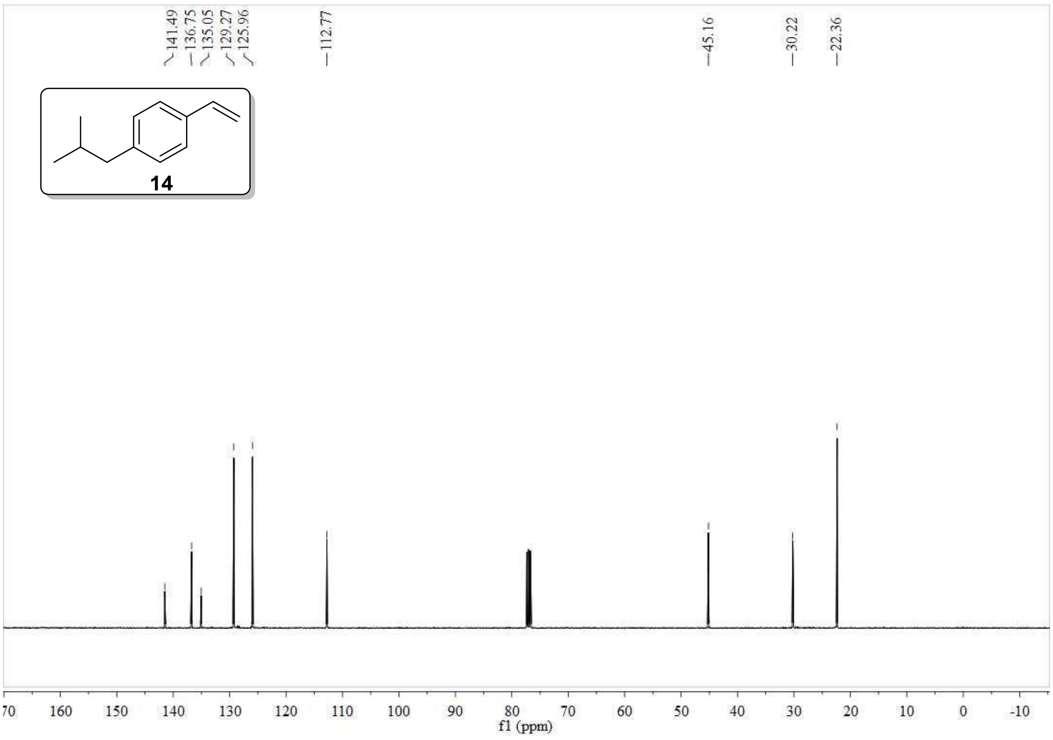

Supplementary Figure 24. ^{13}C NMR spectrum of 1-isobutyl-4-vinylbenzene (14)

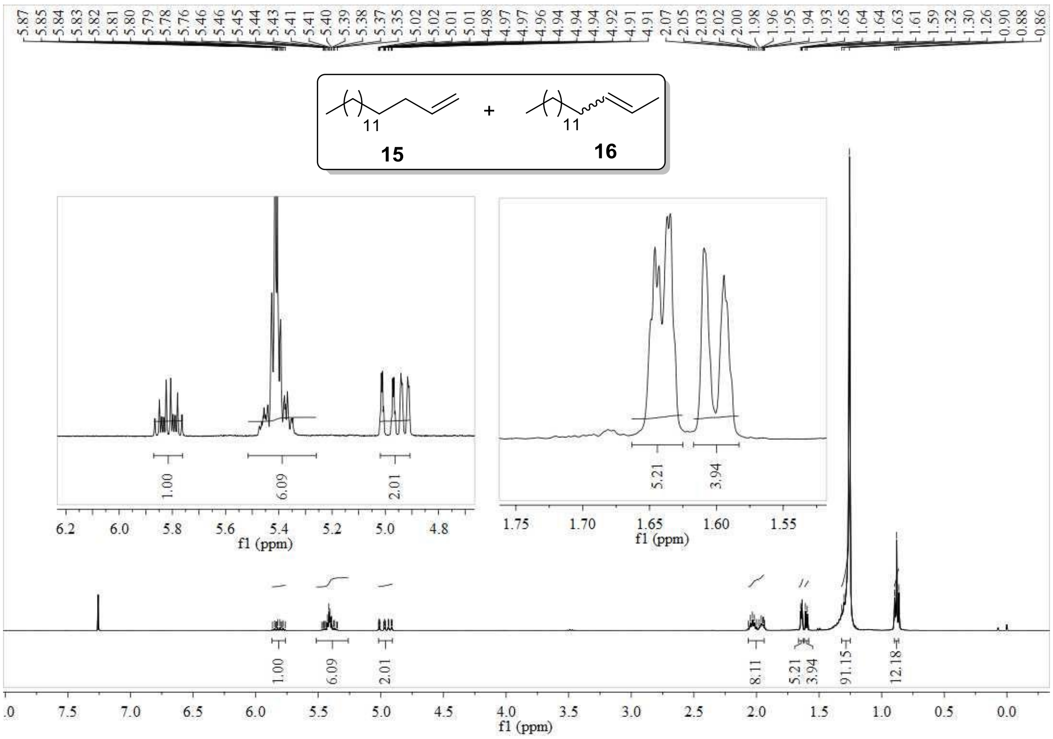

Supplementary Figure 25. ^{1}H NMR spectrum of crude products from S-5

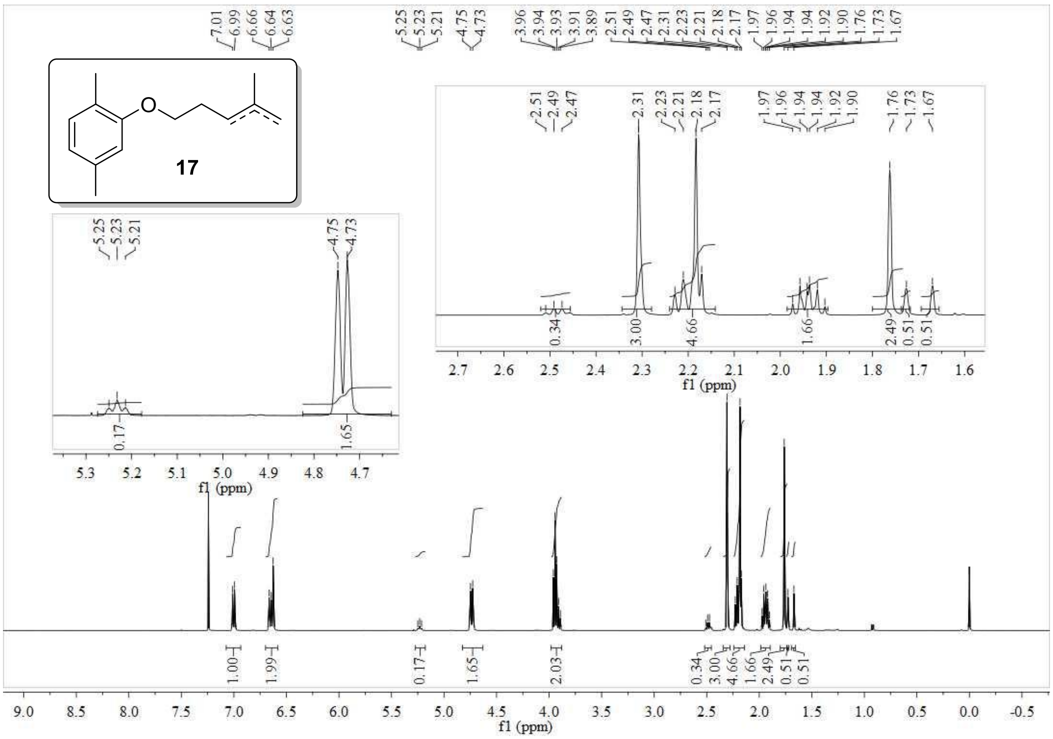

Supplementary Figure 26. ^{1}H NMR spectrum of 17 from S-6

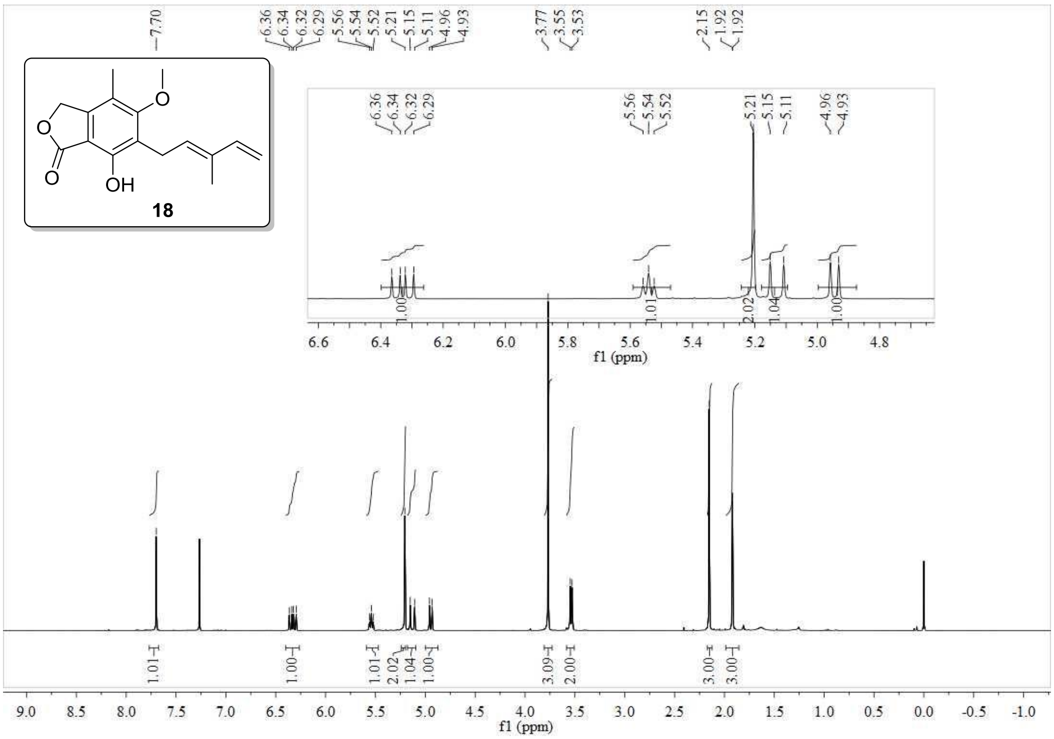

Supplementary Figure 27. ¹H NMR spectrum of product 18

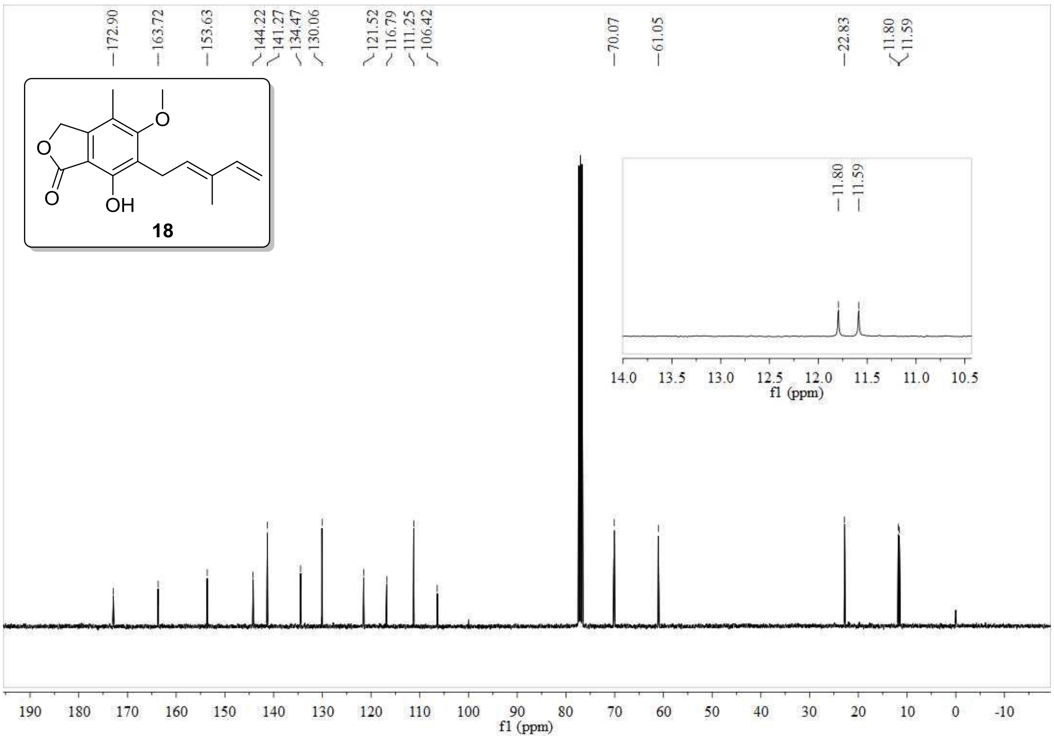

Supplementary Figure 28. ¹³C NMR spectrum of product 18

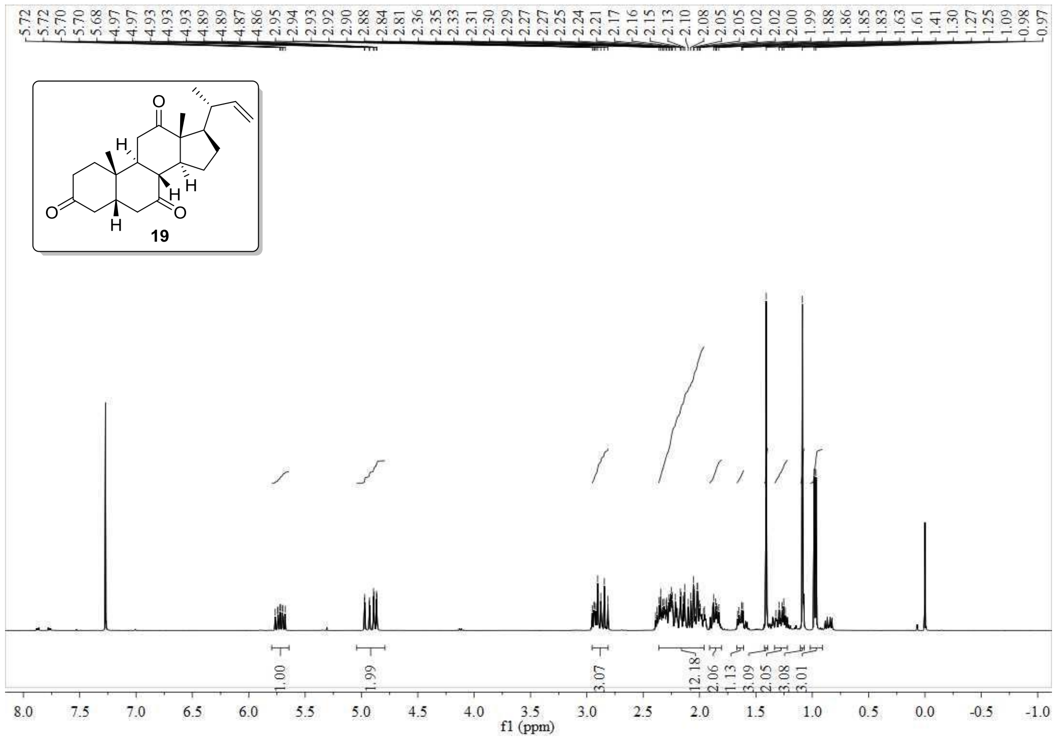

Supplementary Figure 29. ^{1}H NMR spectrum of product 19

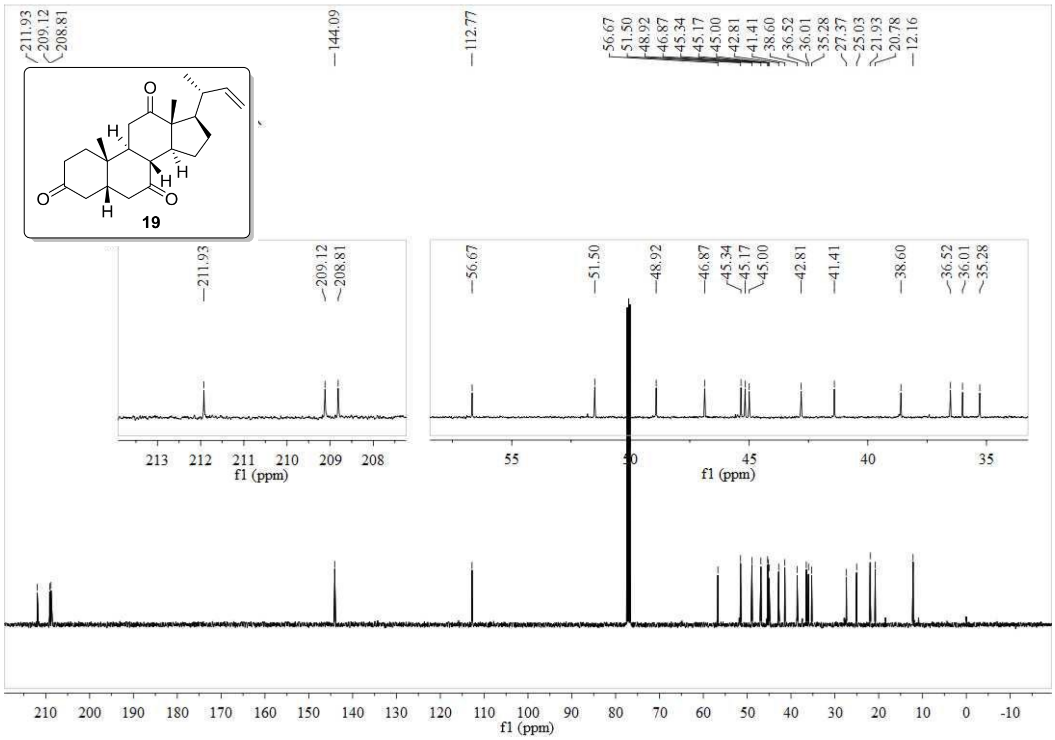

Supplementary Figure 30. ^{13}C NMR spectrum of product 19

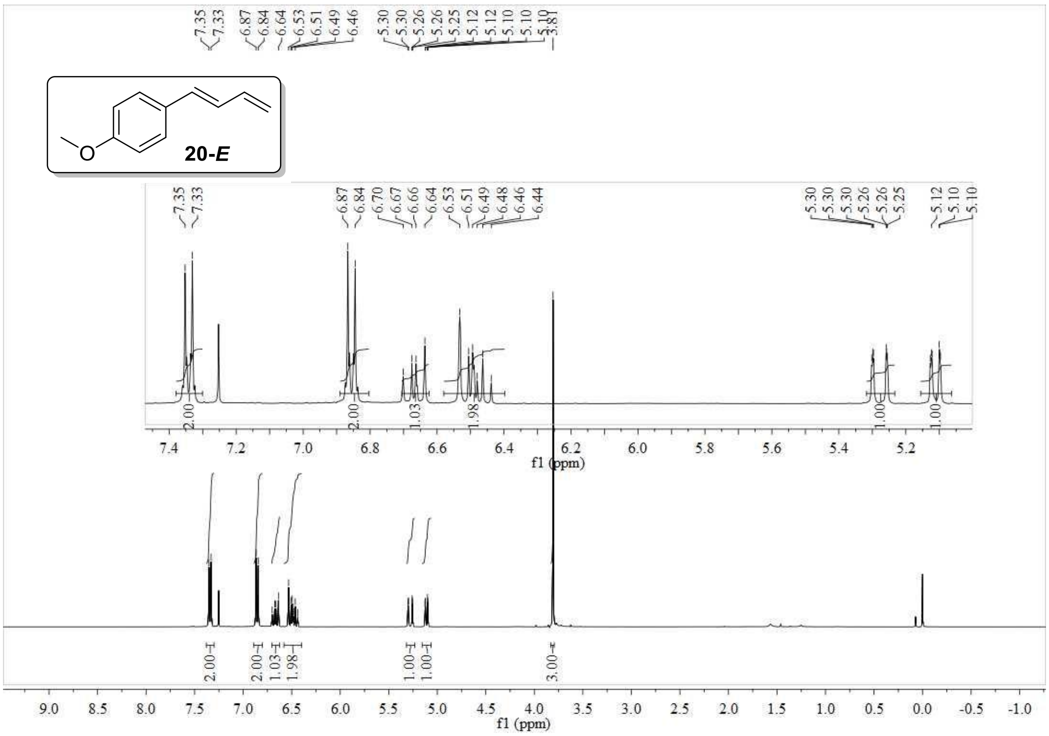

Supplementary Figure 31. **^{1}H NMR spectrum of product 20-*E***

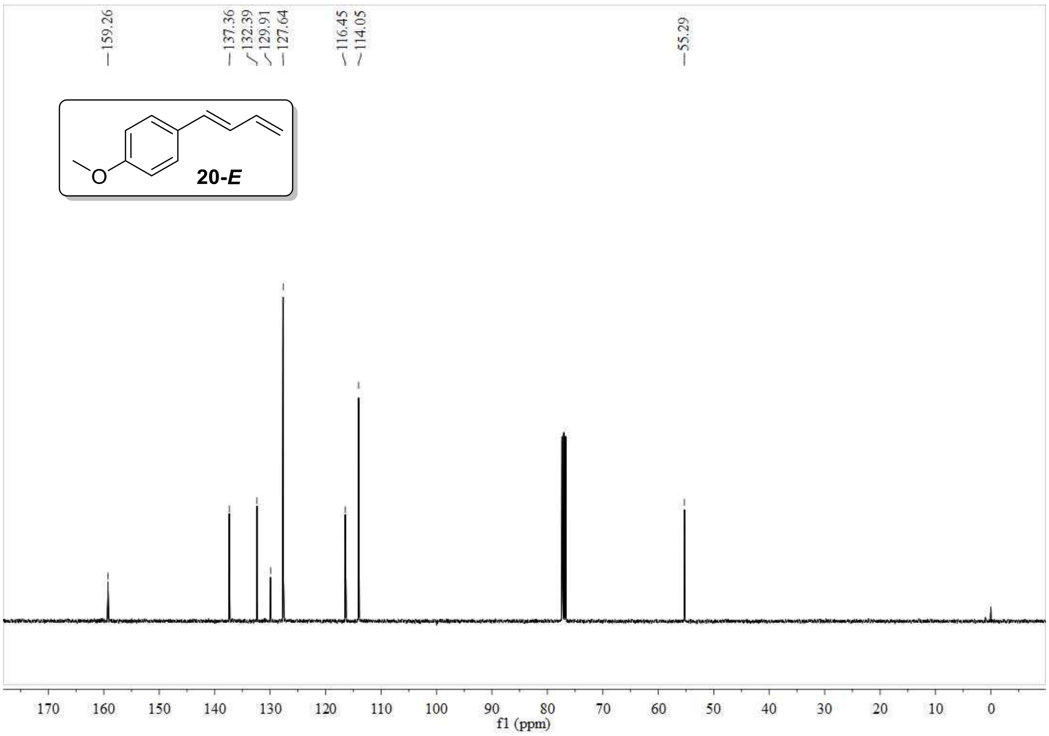

Supplementary Figure 32. **^{13}C NMR spectrum of product 20-*E***

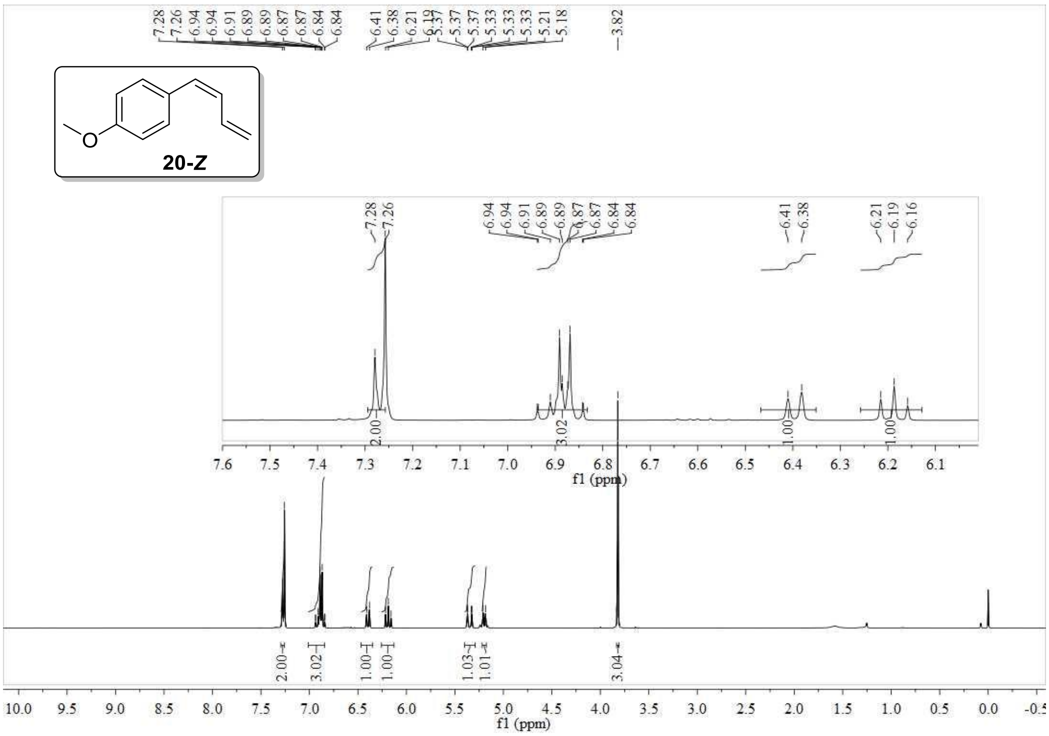

Supplementary Figure 33. ^{1}H NMR spectrum of product 20-*Z*

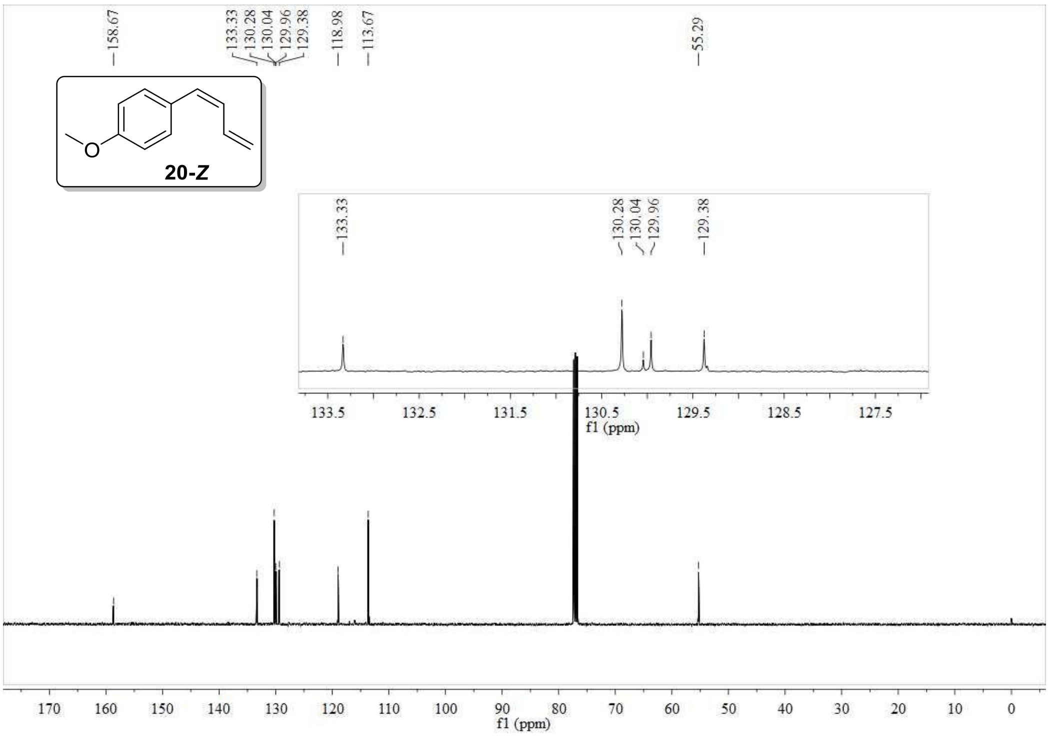

Supplementary Figure 34. ^{13}C NMR spectrum of product 20-*Z*

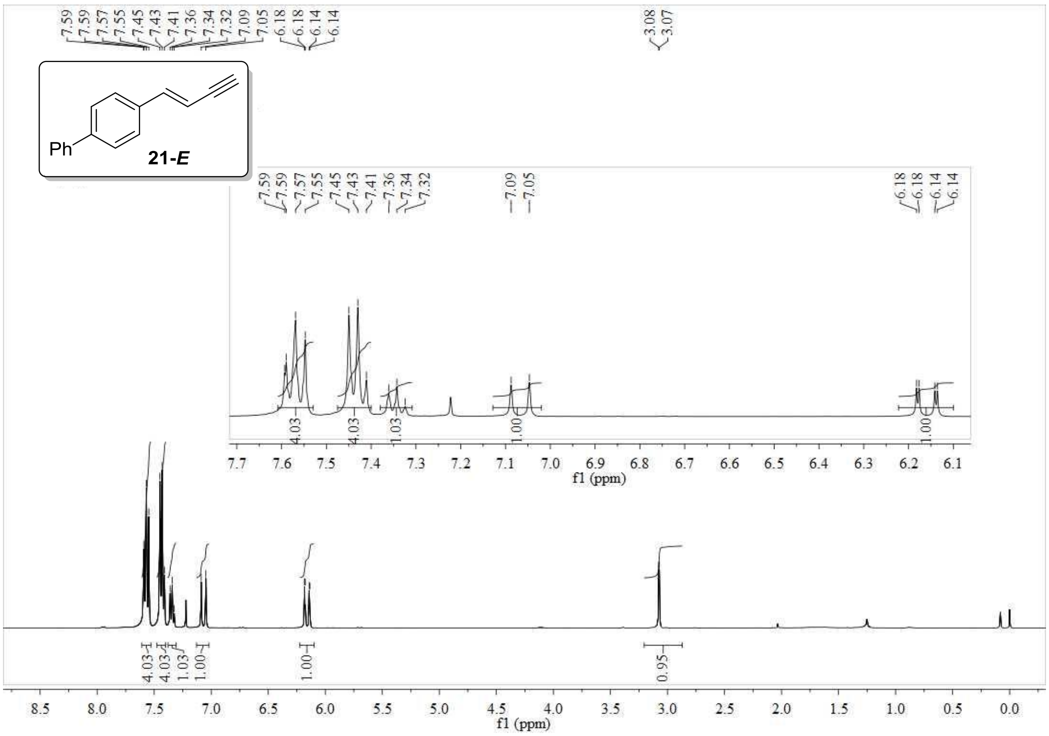

Supplementary Figure 35. ^{1}H NMR spectrum of (*E*)-4-(but-1-en-3-yn-1-yl)-1,1'-biphenyl (21-*E*)

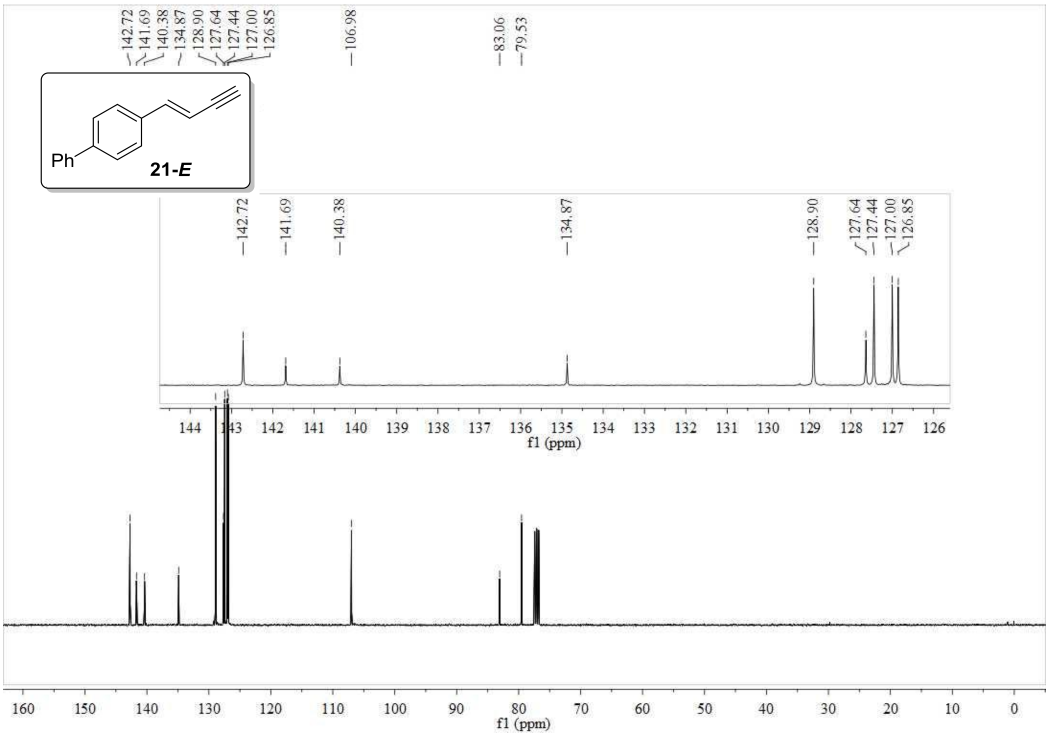

Supplementary Figure 36. ^{13}C NMR spectrum of (*E*)-4-(but-1-en-3-yn-1-yl)-1,1'-biphenyl (21-*E*)

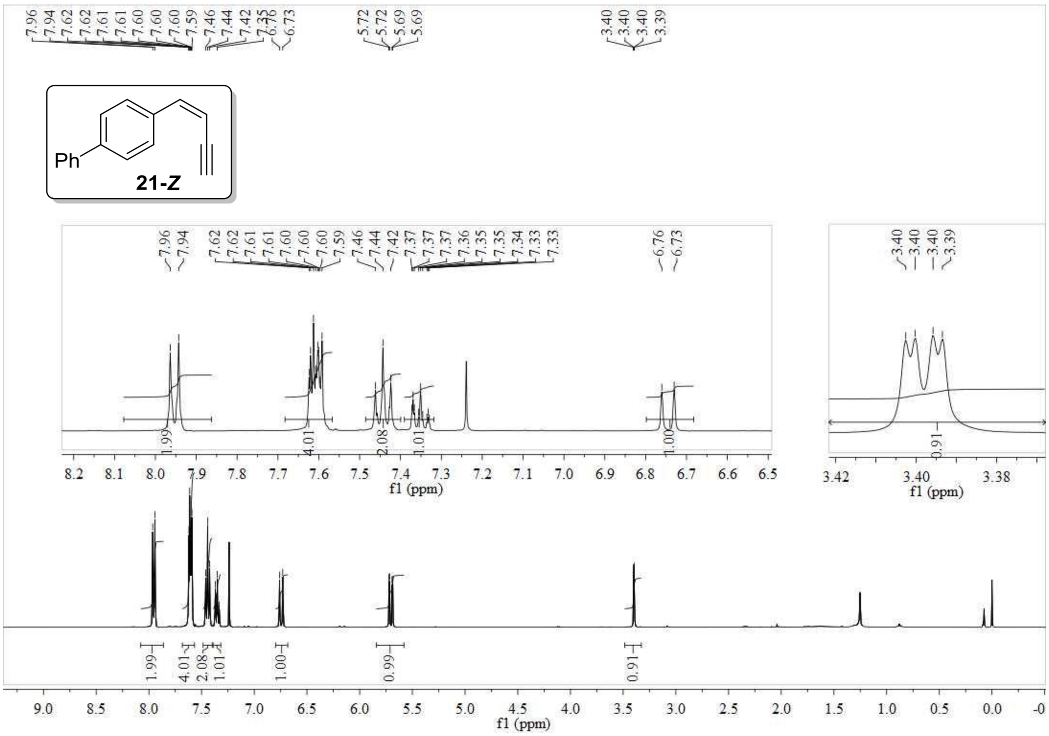

Supplementary Figure 37. ^{1}H NMR spectrum of (*Z*)-4-(but-1-en-3-yn-1-yl)-1,1'-biphenyl (21-*Z*)

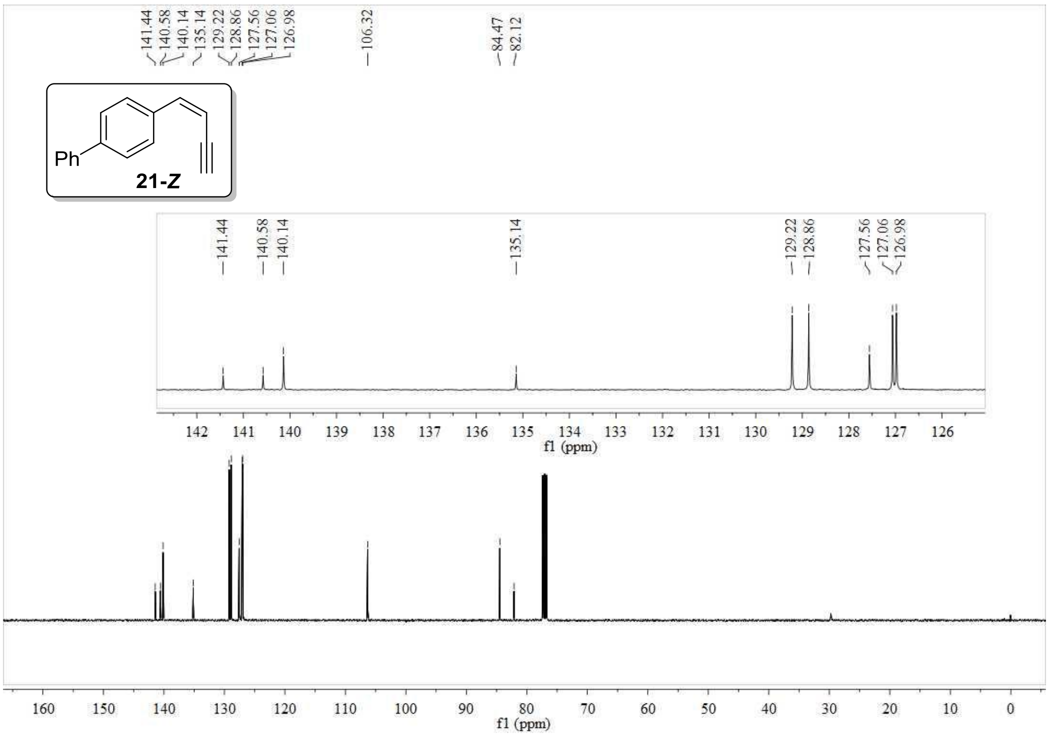

Supplementary Figure 38. ^{13}C NMR spectrum of (*Z*)-4-(but-1-en-3-yn-1-yl)-1,1'-biphenyl (21-*Z*)

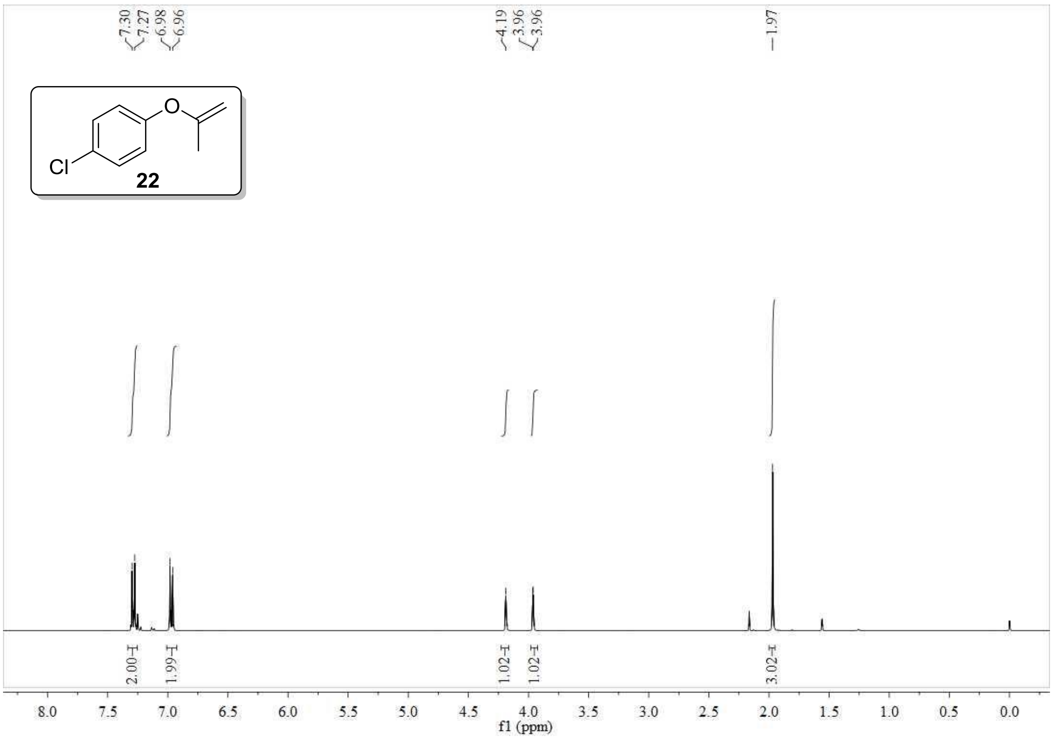

Supplementary Figure 39. ^{1}**H NMR spectrum of 1-chloro-4-(prop-1-en-2-yloxy)benzene (22)**

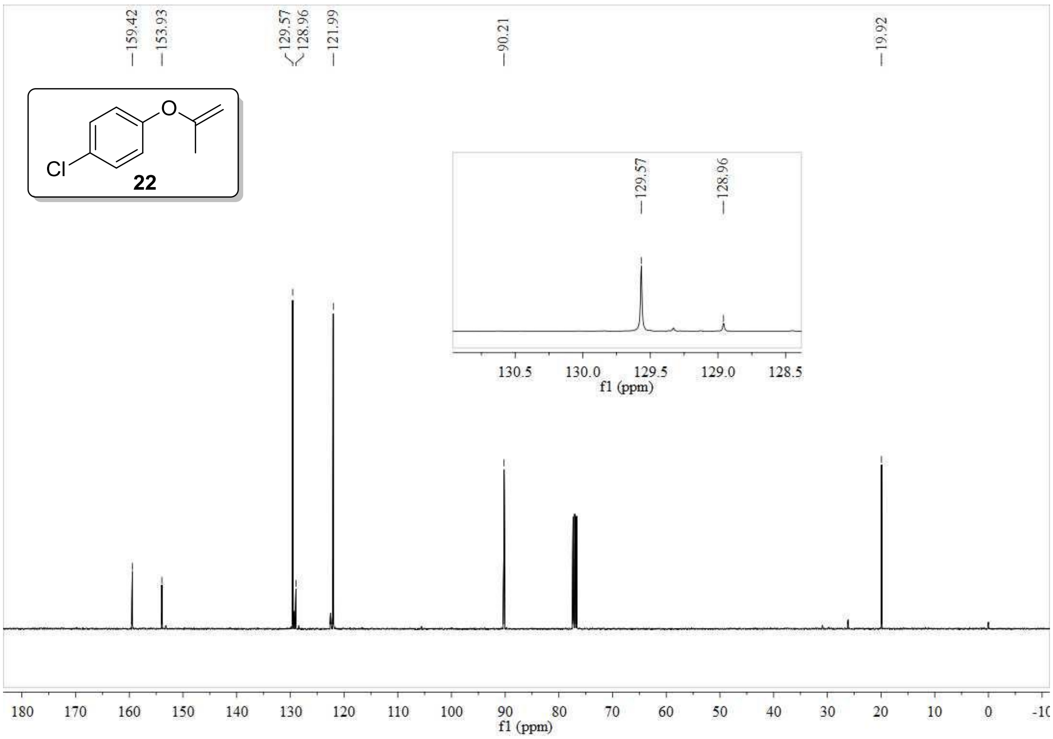

Supplementary Figure 40. ^{13}**C NMR spectrum of 1-chloro-4-(prop-1-en-2-yloxy)benzene (22)**

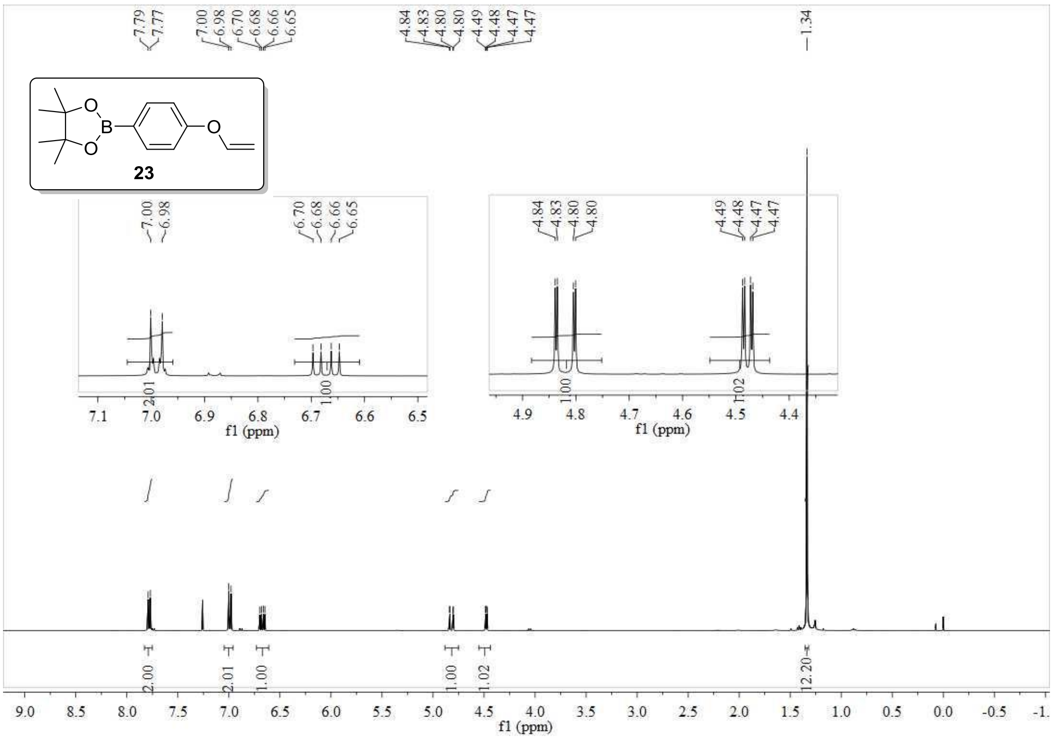

Supplementary Figure 41. ^{1}H NMR spectrum of product 23

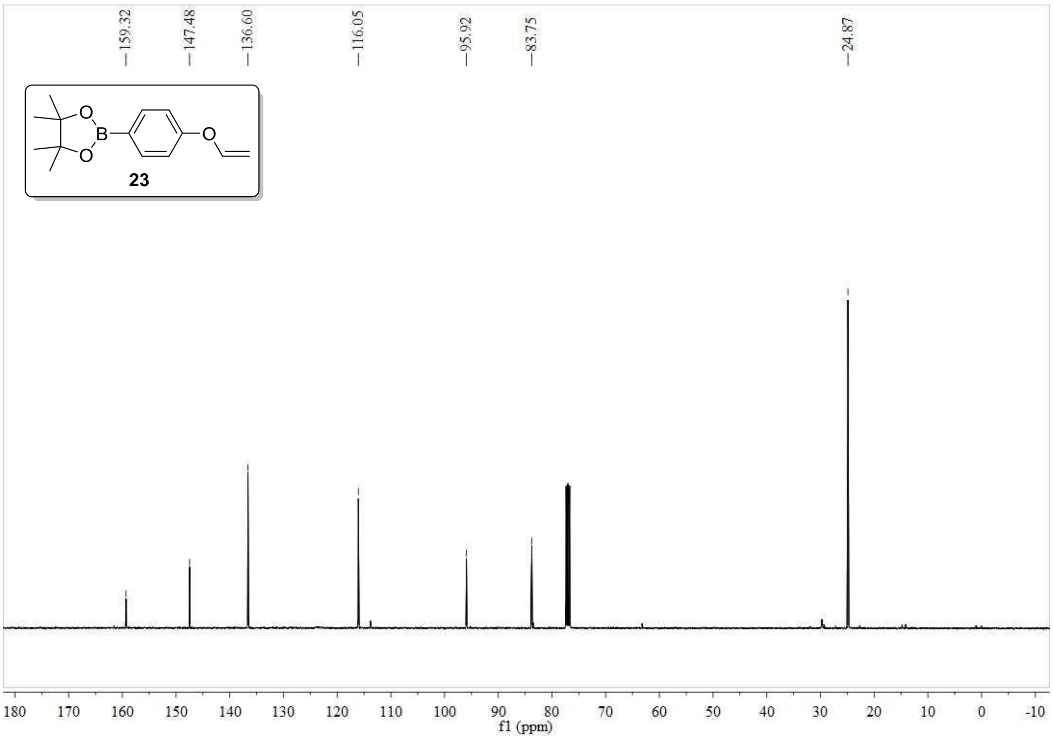

Supplementary Figure 42. ^{13}C NMR spectrum of product 23

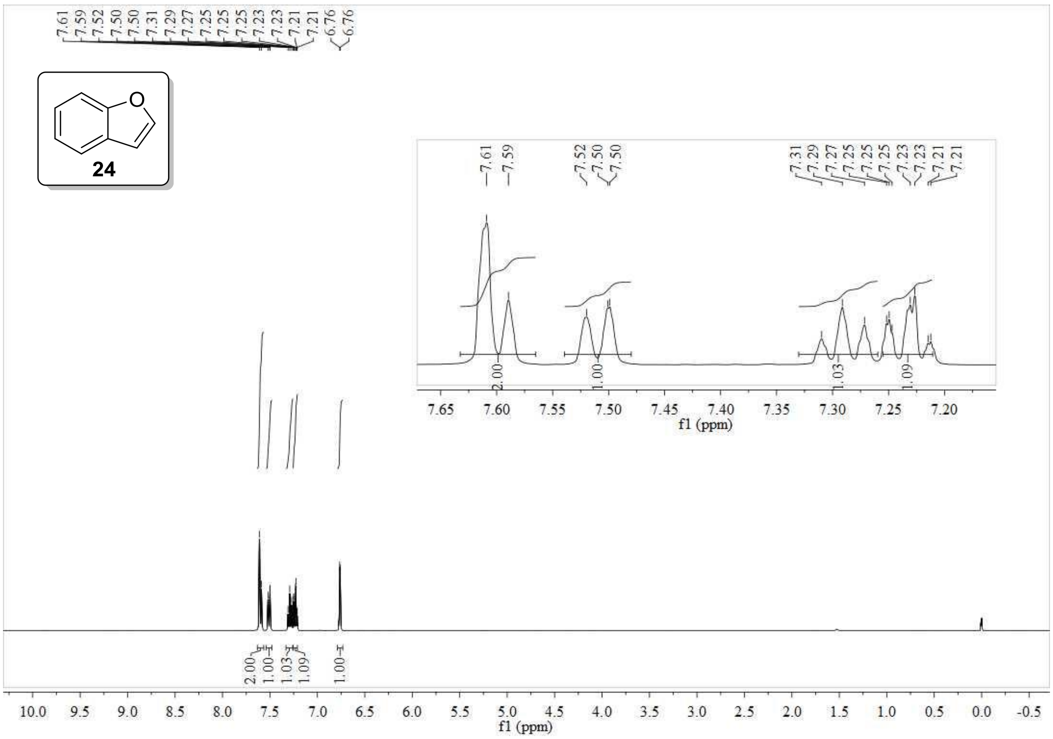

Supplementary Figure 43. ^{1}H NMR spectrum of benzofuran (24)

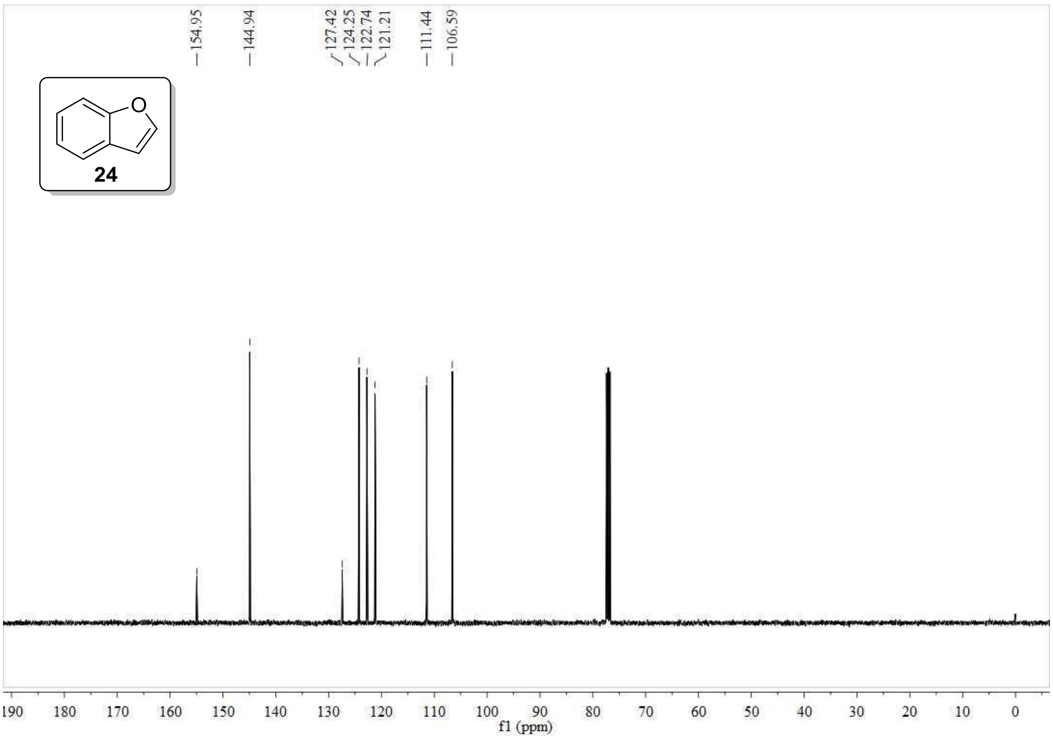

Supplementary Figure 44. ^{13}C NMR spectrum of benzofuran (24)

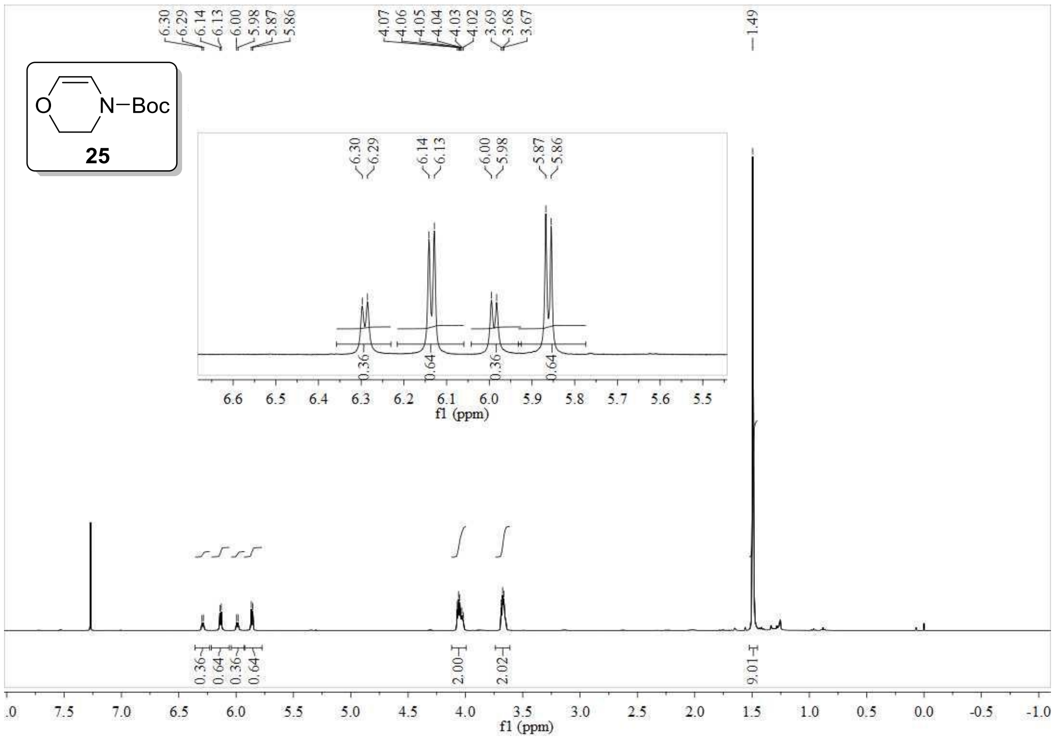

Supplementary Figure 45. ^{1}H NMR spectrum of product 25

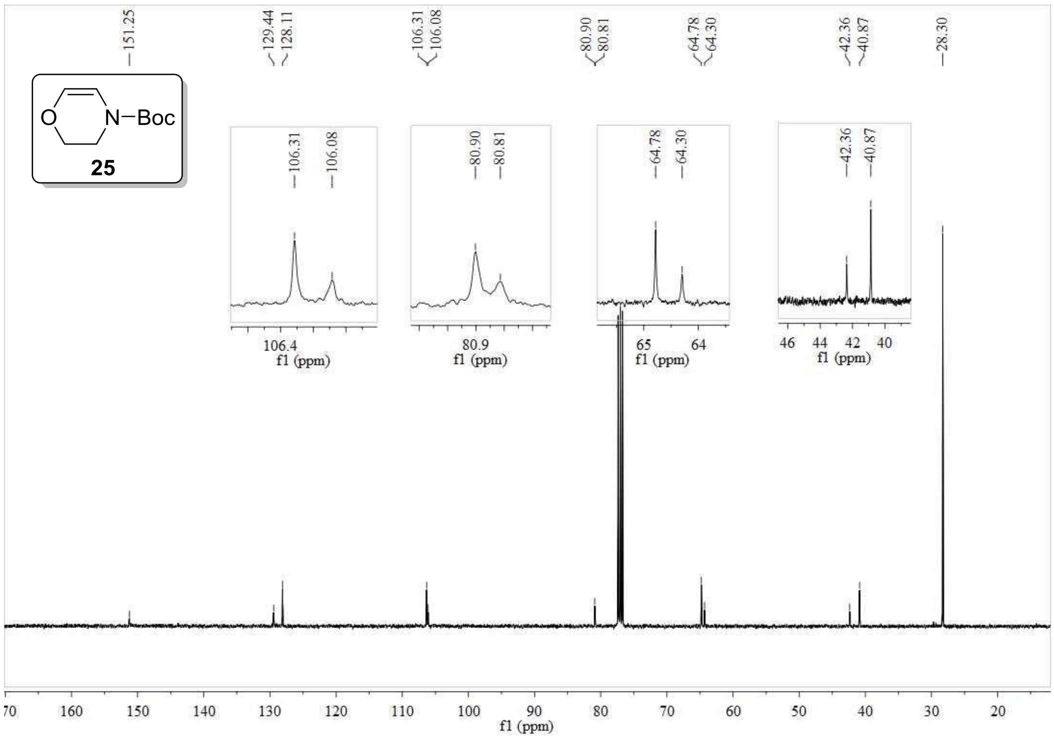

Supplementary Figure 46. ^{13}C NMR spectrum of product 25

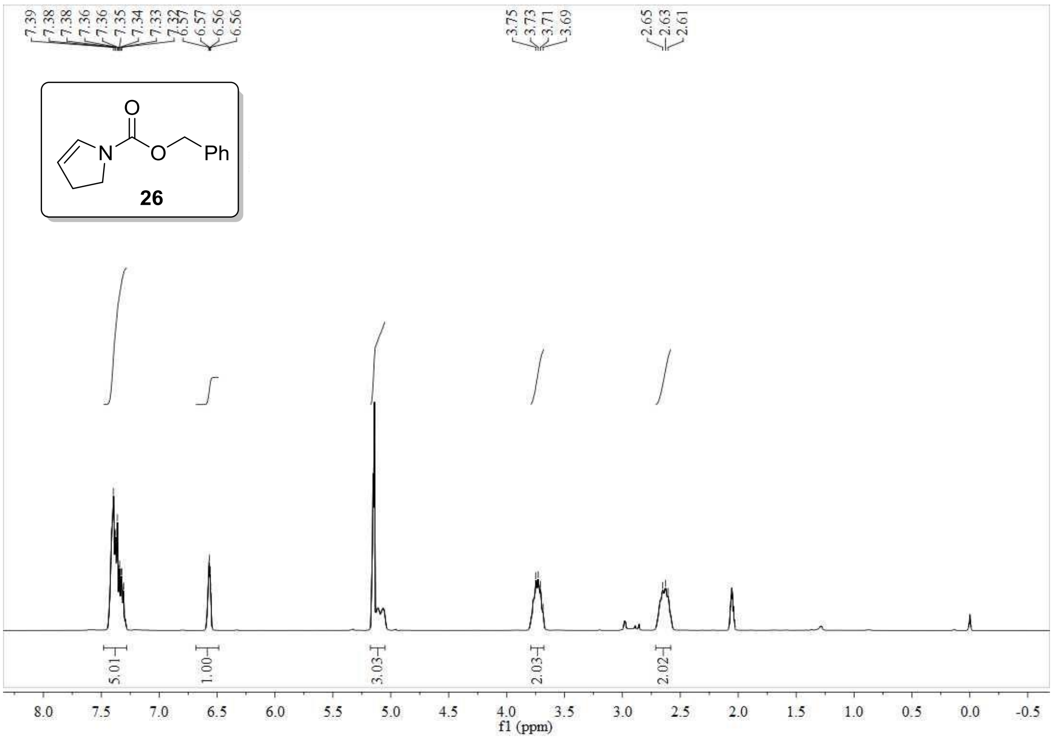

Supplementary Figure 47. ^{1}H NMR spectrum of product 26

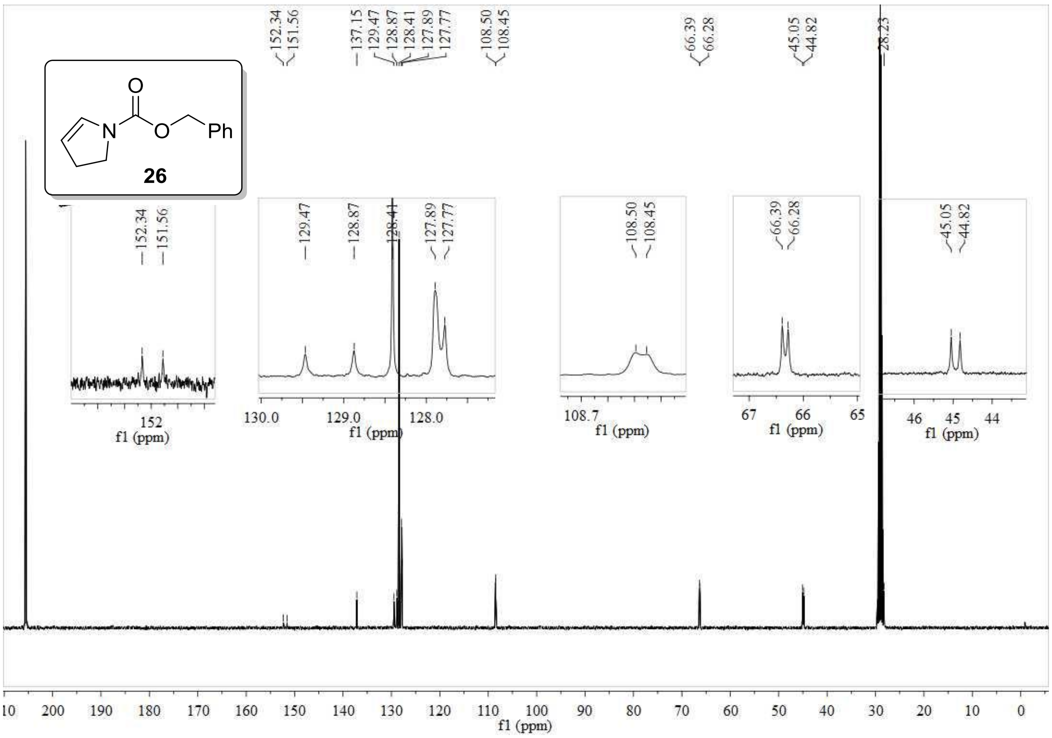

Supplementary Figure 48. ^{13}C NMR spectrum of product 26

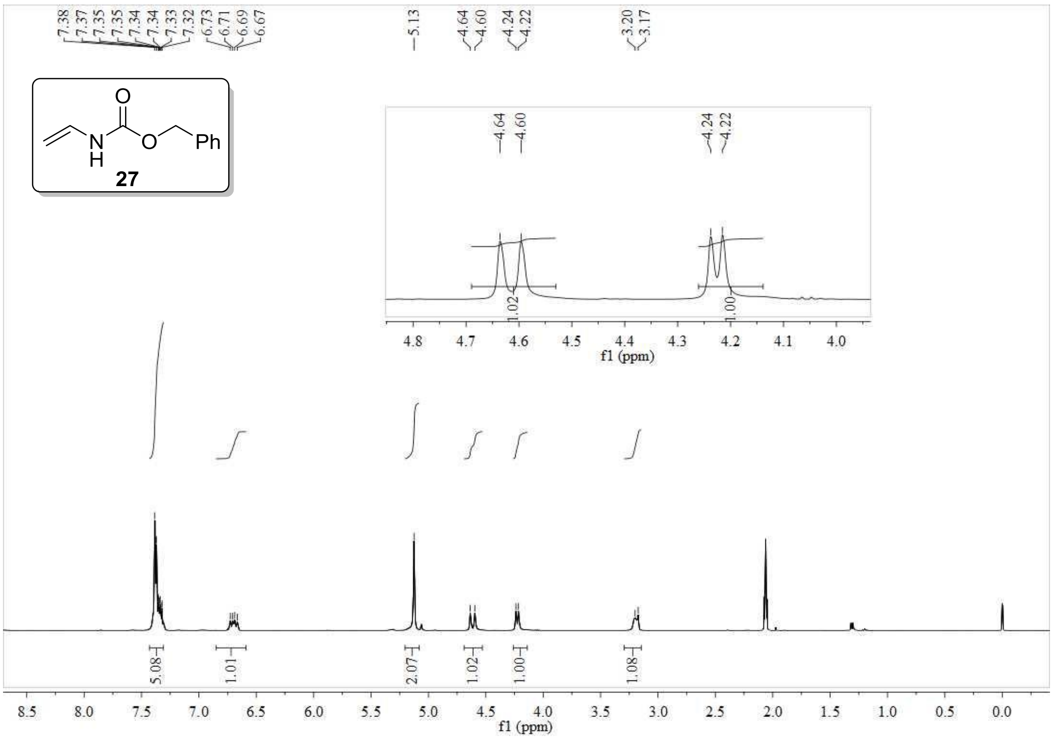

Supplementary Figure 49. ^{1}H NMR spectrum of benzyl vinylcarbamate (27)

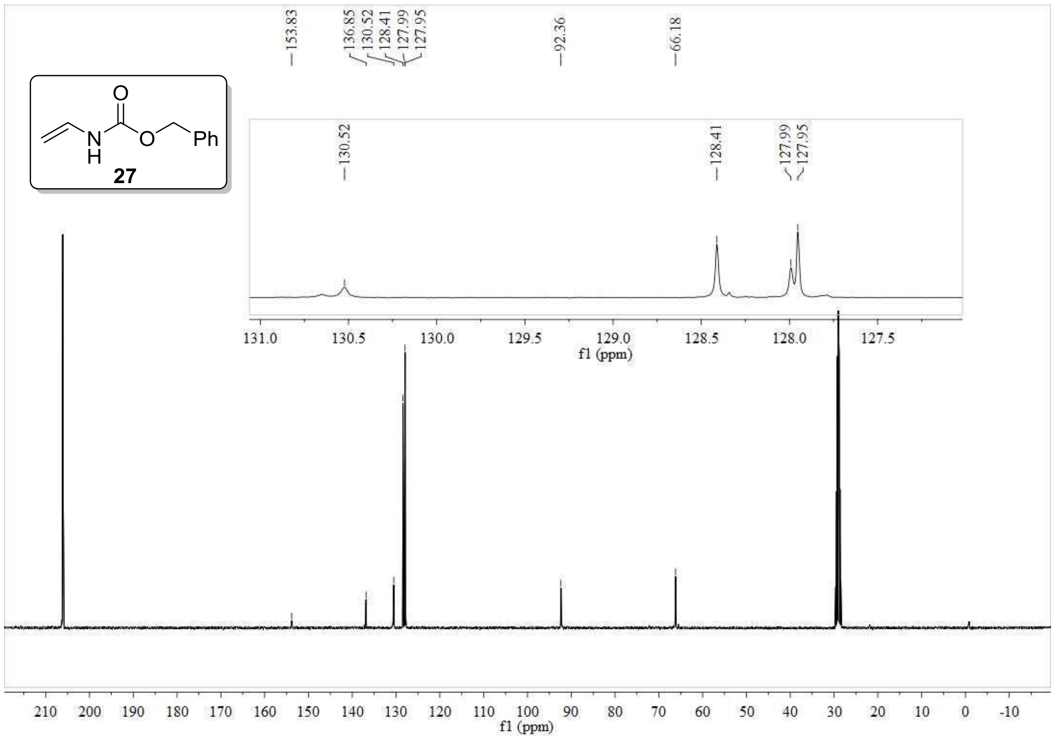

Supplementary Figure 50. ^{13}C NMR spectrum of benzyl vinylcarbamate (27)

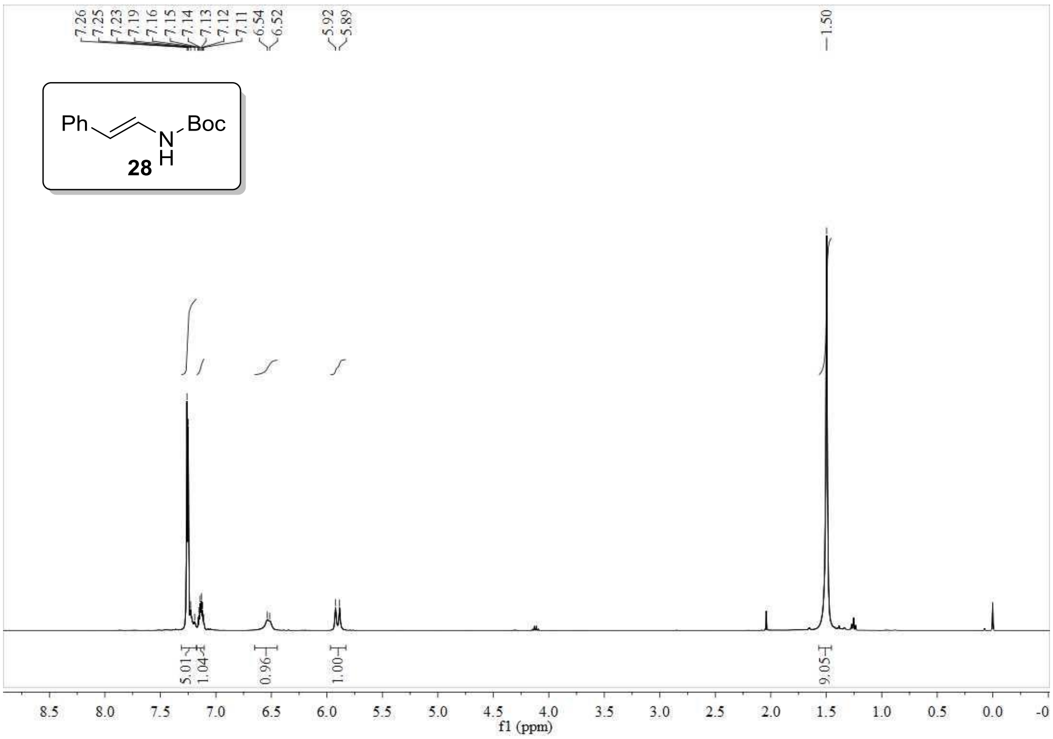

Supplementary Figure 51. ^{1}H NMR spectrum of (*E*)-*tert*-butyl styrylcarbamate (28)

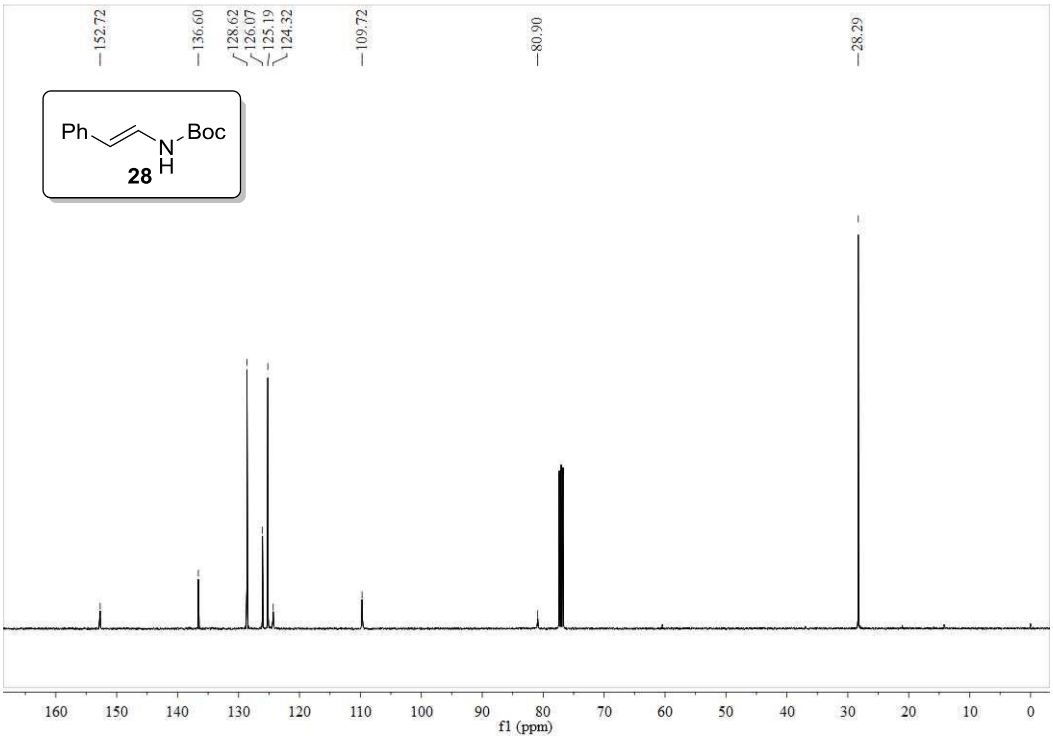

Supplementary Figure 52. ^{13}C NMR spectrum of (*E*)-*tert*-butyl styrylcarbamate (28)

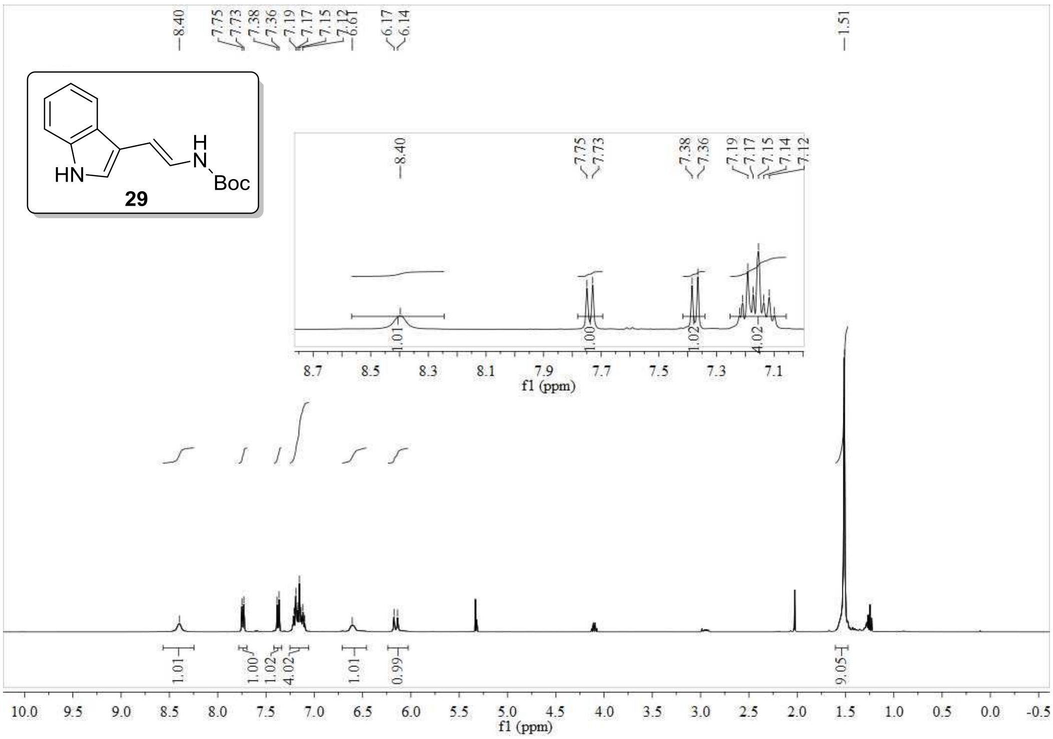

Supplementary Figure 53. ^{1}H NMR spectrum of product 29

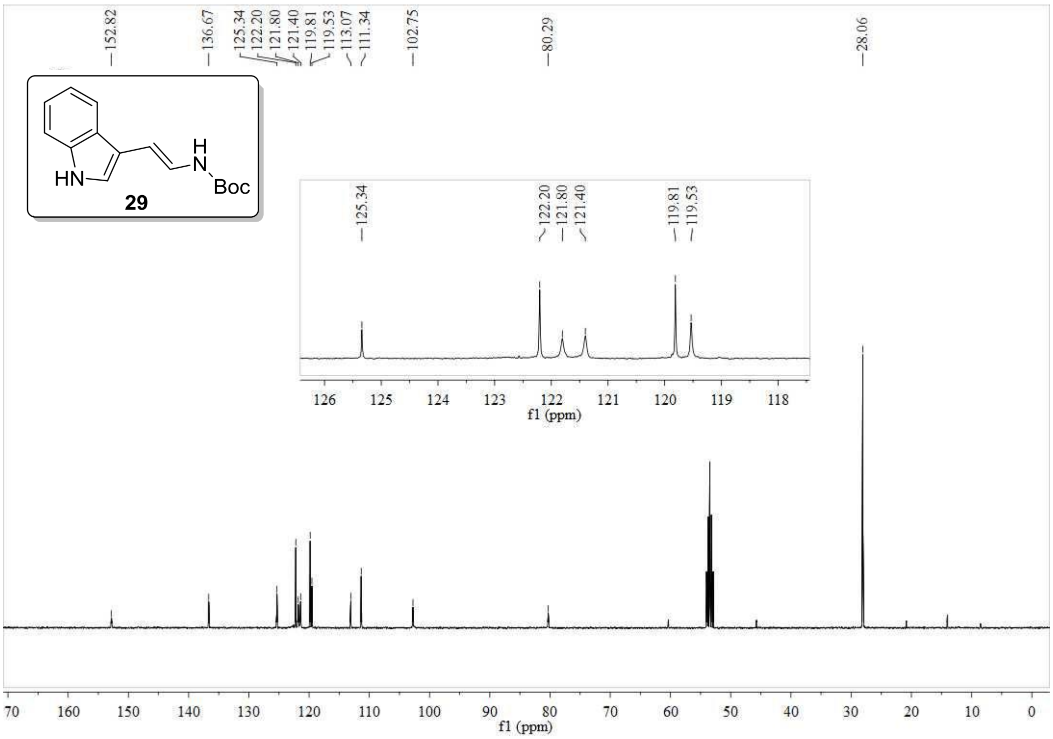

Supplementary Figure 54. ^{13}C NMR spectrum of product 29

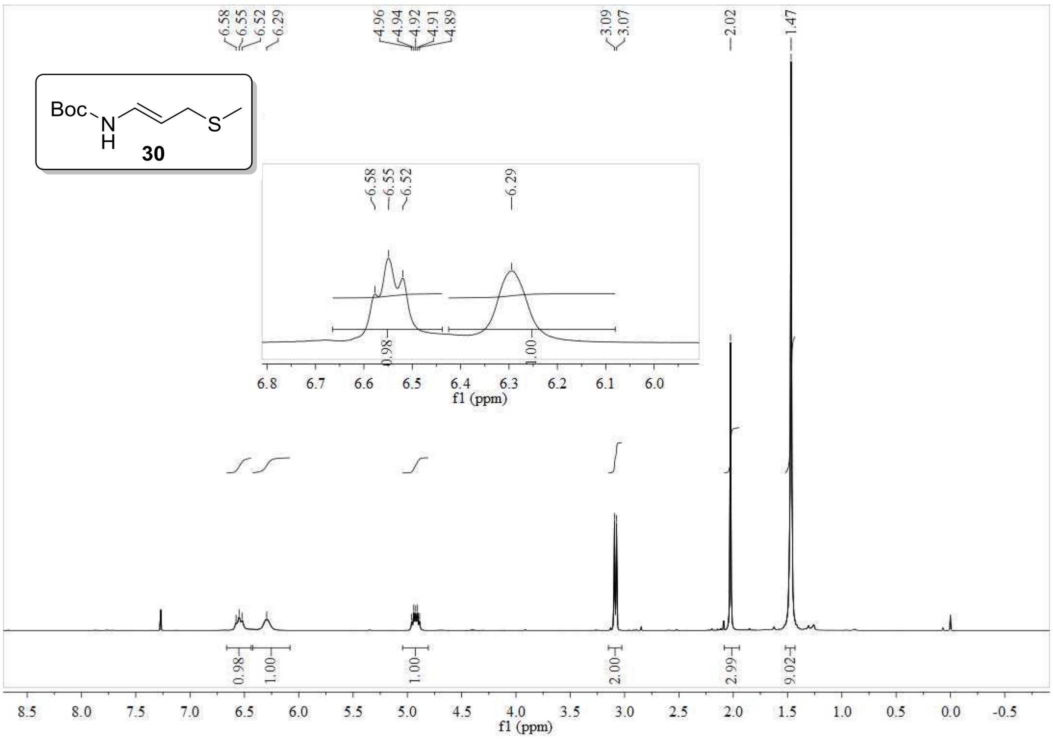

Supplementary Figure 55. ^{1}H NMR spectrum of product 30

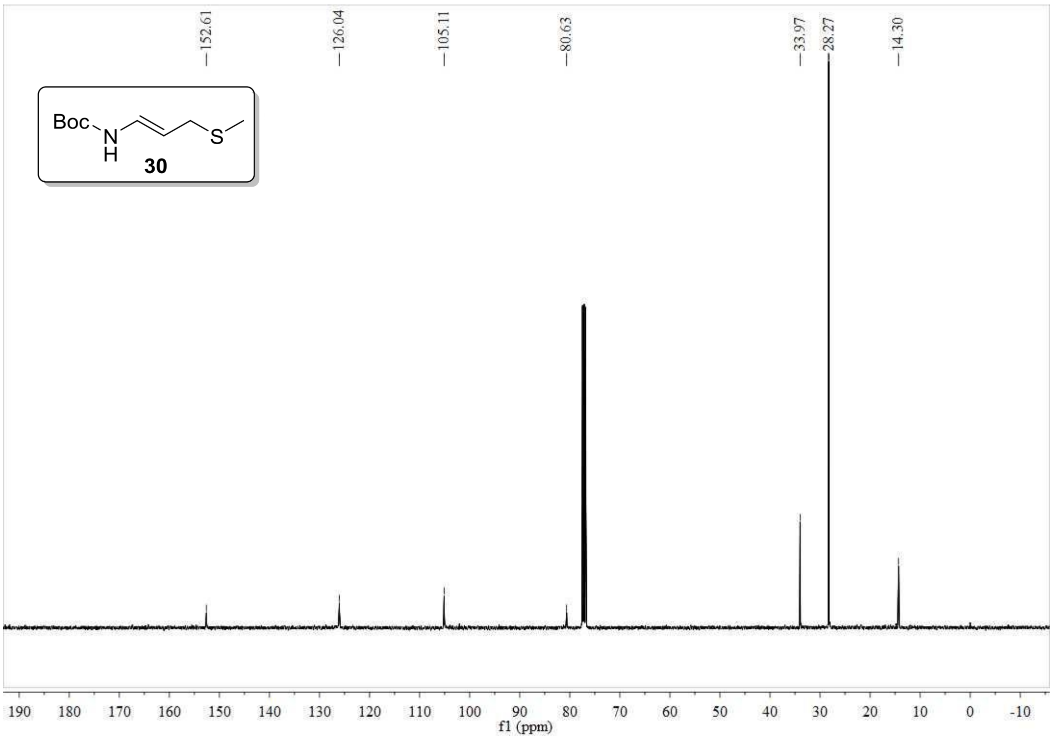

Supplementary Figure 56. ^{13}C NMR spectrum of product 30

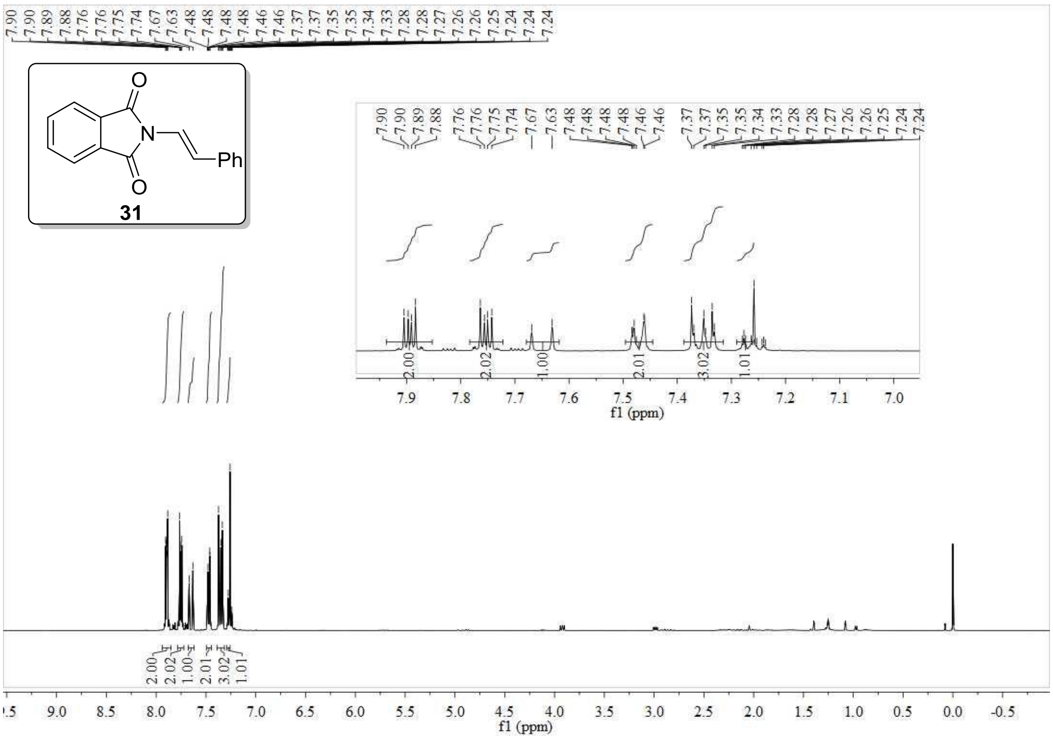

Supplementary Figure 57. ^{1}H NMR spectrum of (*E*)-2-styrylisoindoline-1,3-dione (31)

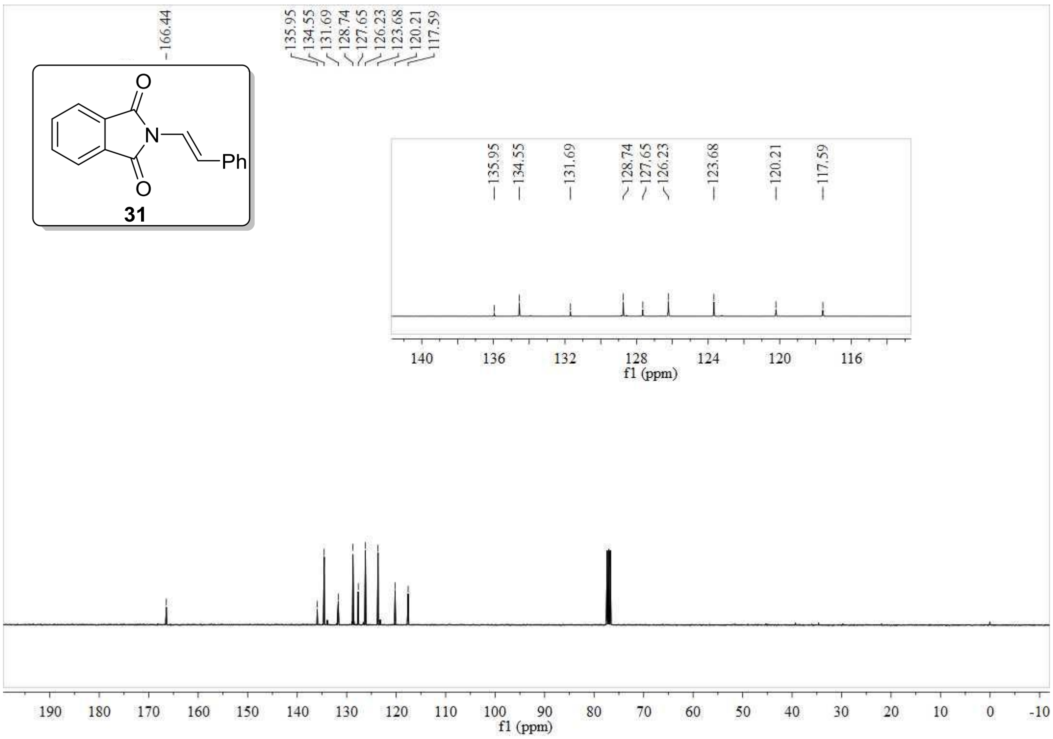

Supplementary Figure 58. ^{13}C NMR spectrum of (*E*)-2-styrylisoindoline-1,3-dione (31)

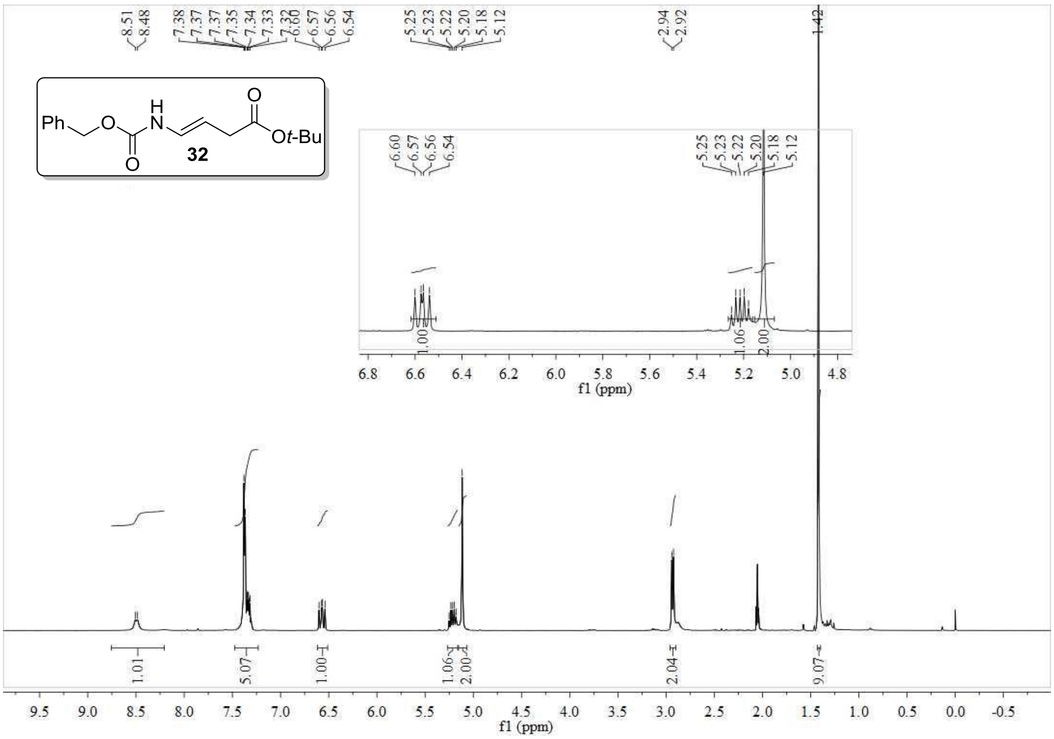

Supplementary Figure 59. ^{1}**H NMR spectrum of product 32**

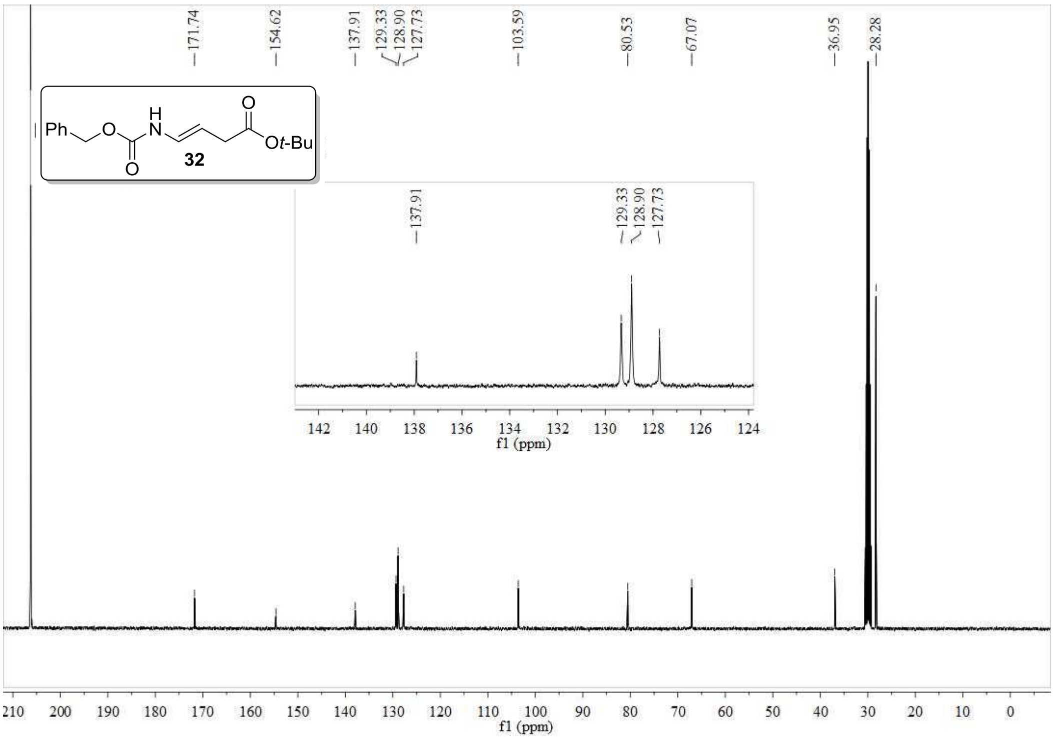

Supplementary Figure 60. ^{13}**C NMR spectrum of product 32**

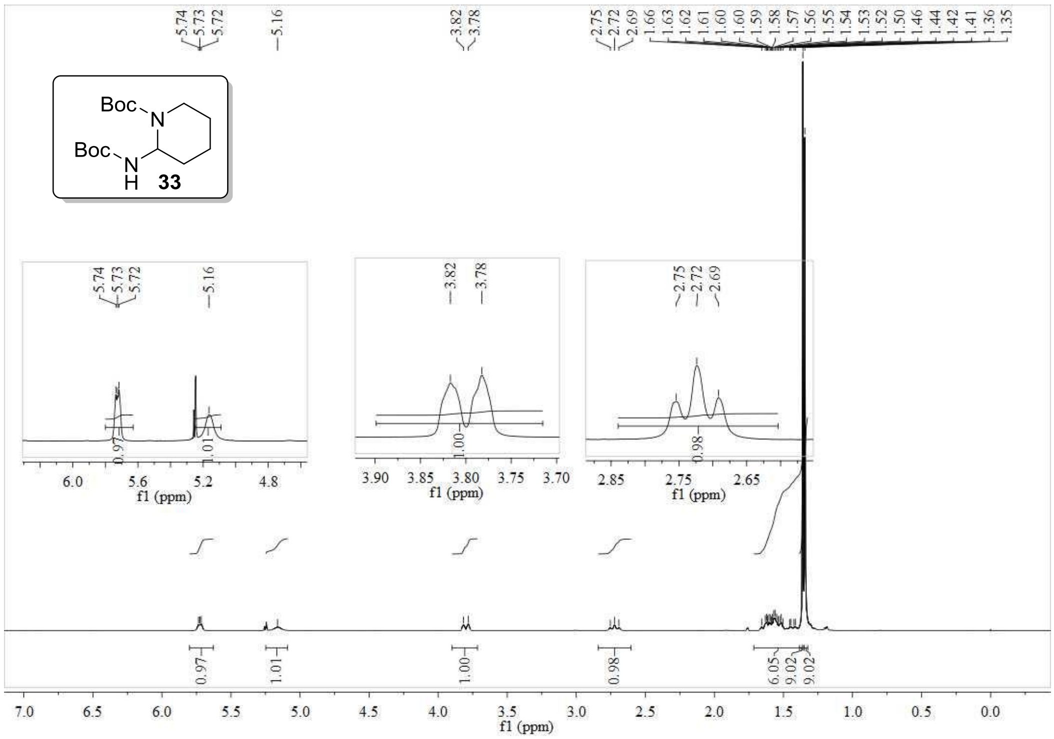

Supplementary Figure 61. ^{1}H NMR spectrum of product 33

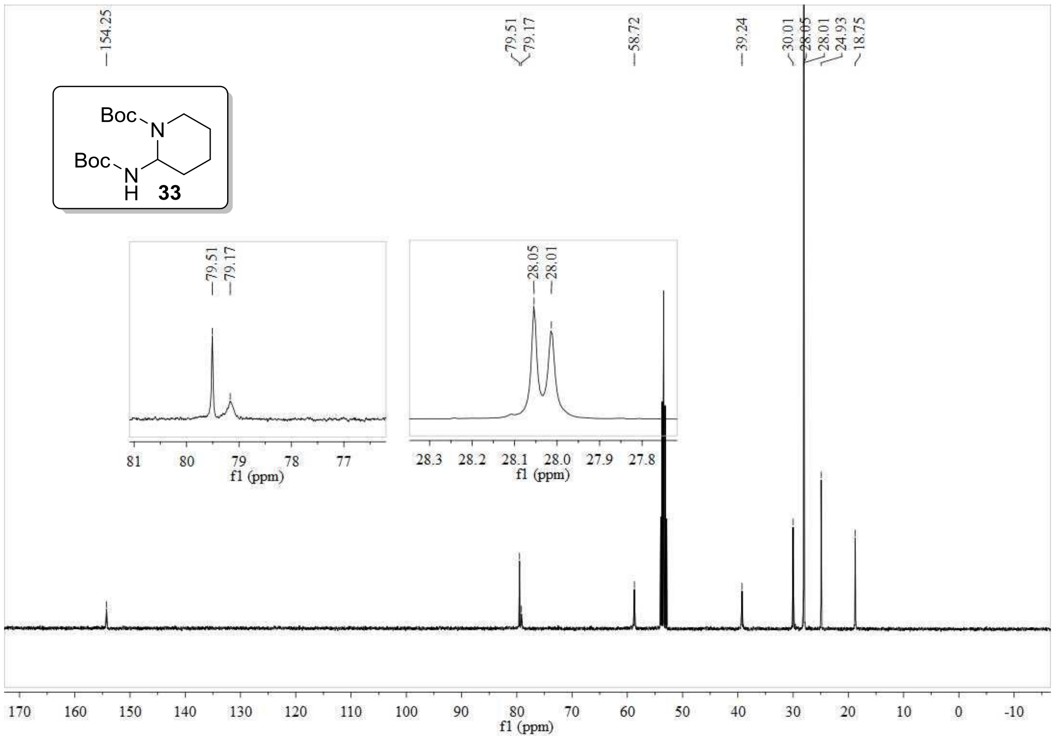

Supplementary Figure 62. ^{13}C NMR spectrum of product 33

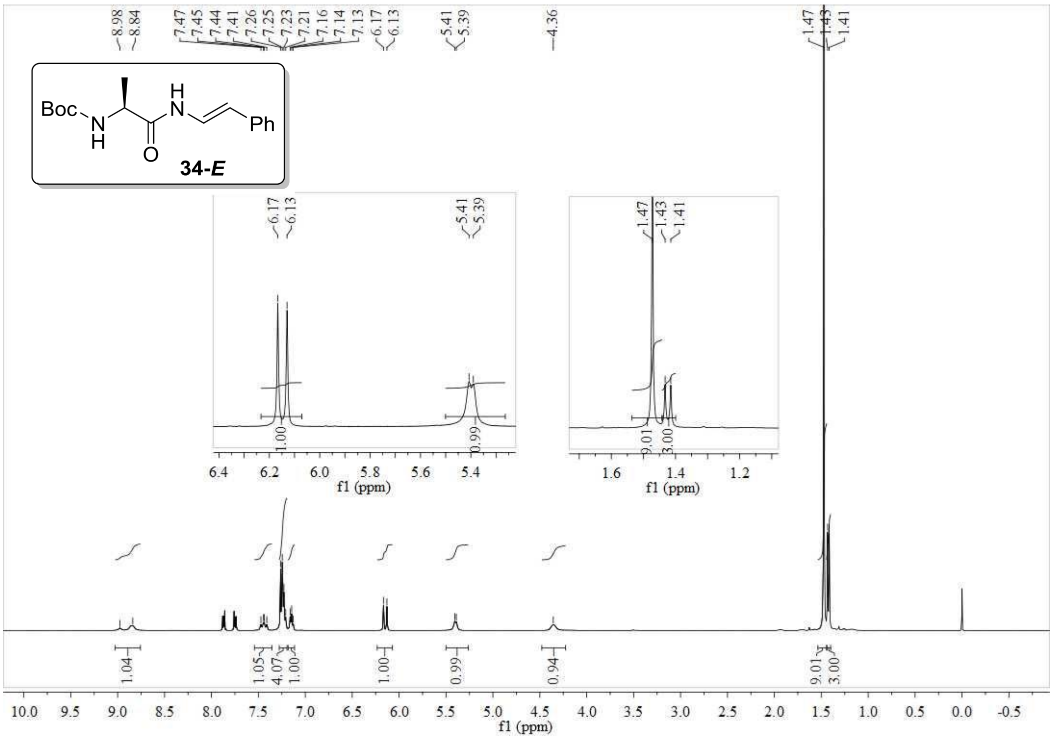

Supplementary Figure 63. ^{1}H NMR spectrum of product 34-*E*

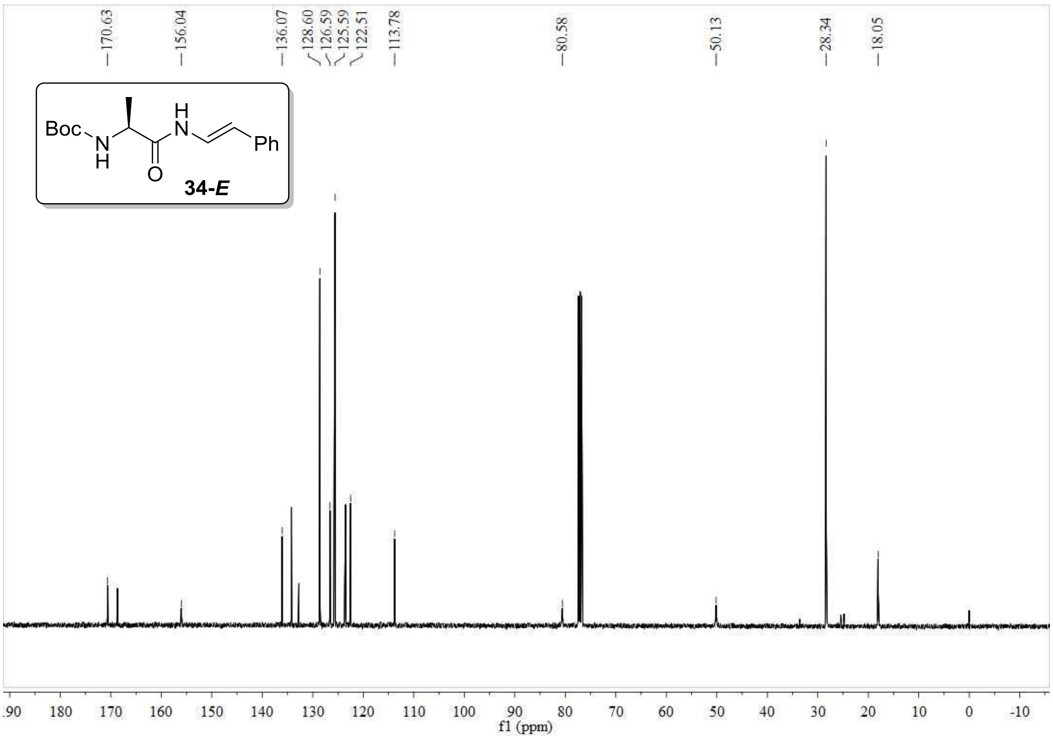

Supplementary Figure 64. ^{13}C NMR spectrum of product 34-*E*

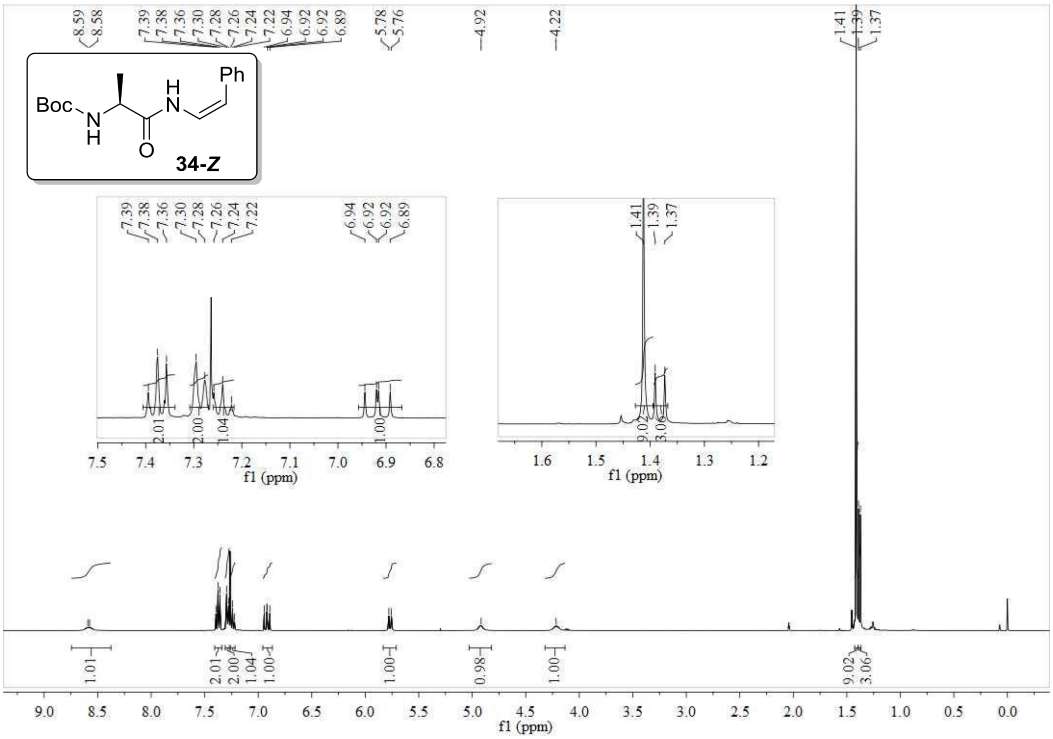

Supplementary Figure 65. ^{1}H NMR spectrum of product 34-*Z*

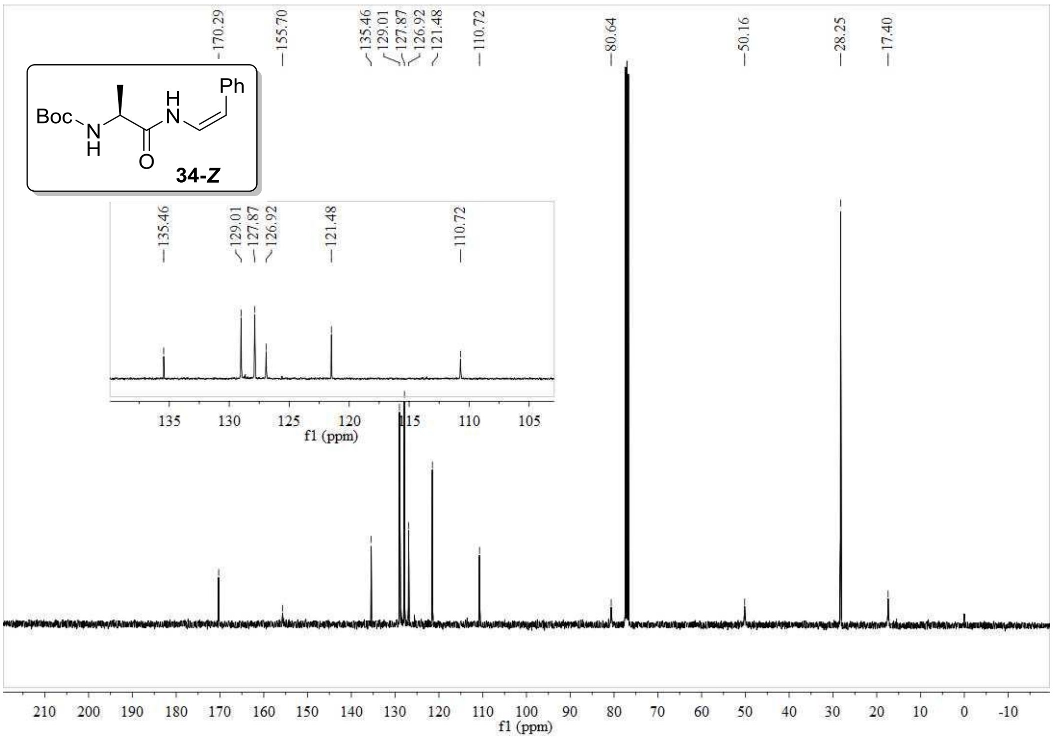

Supplementary Figure 66. ^{13}C NMR spectrum of product 34-*Z*

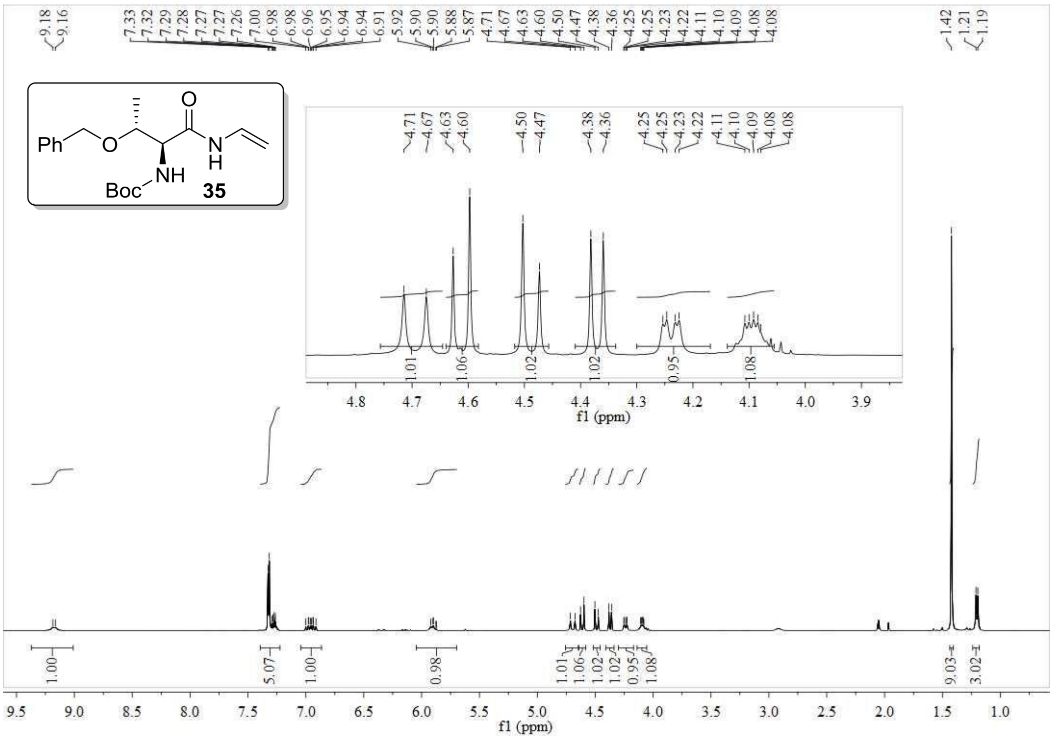

Supplementary Figure 67. ^{1}H NMR spectrum of product 35

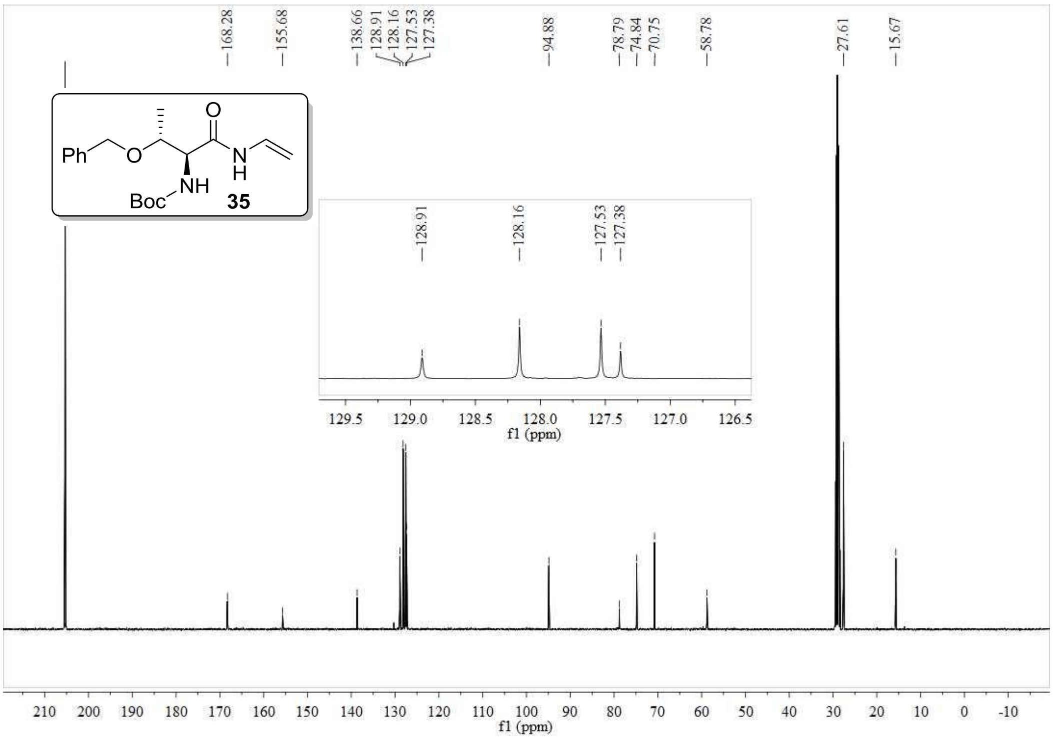

Supplementary Figure 68. ^{13}C NMR spectrum of product 35

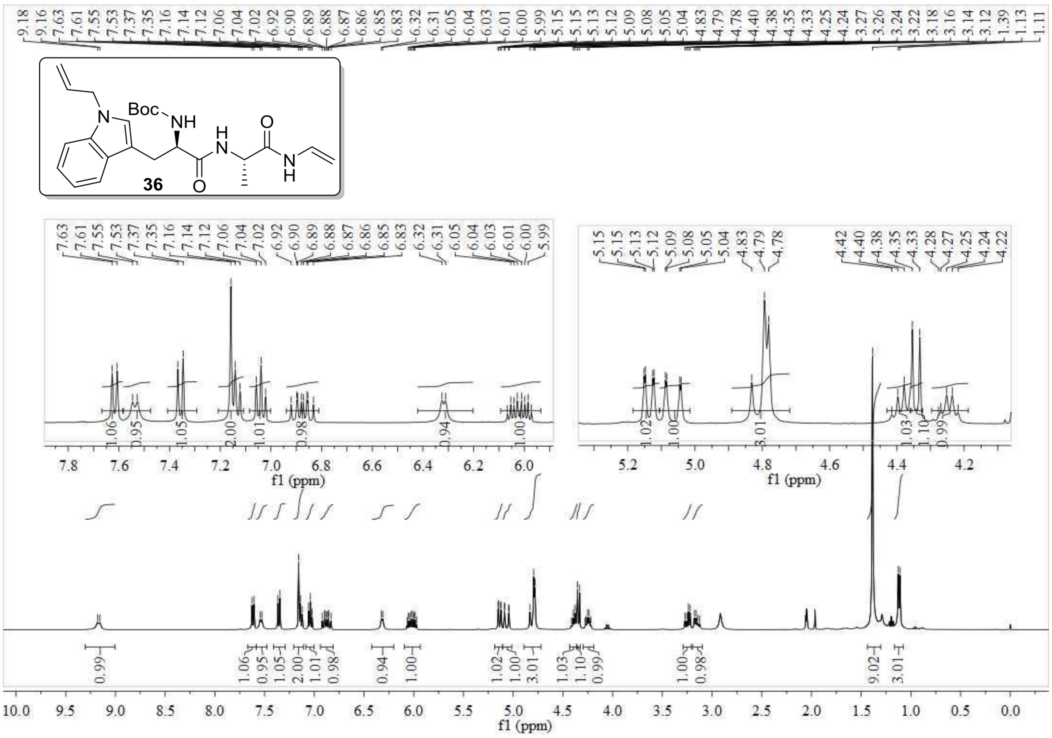

Supplementary Figure 69. ^{1}H NMR spectrum of product 36

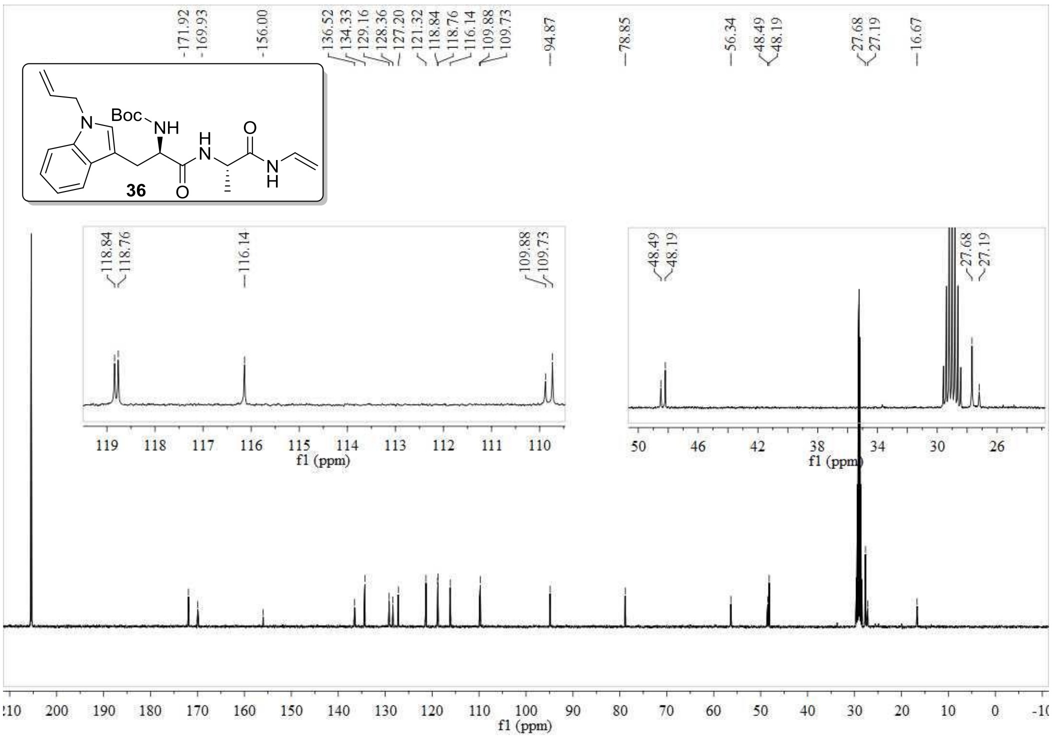

Supplementary Figure 70. ^{13}C NMR spectrum of product 36

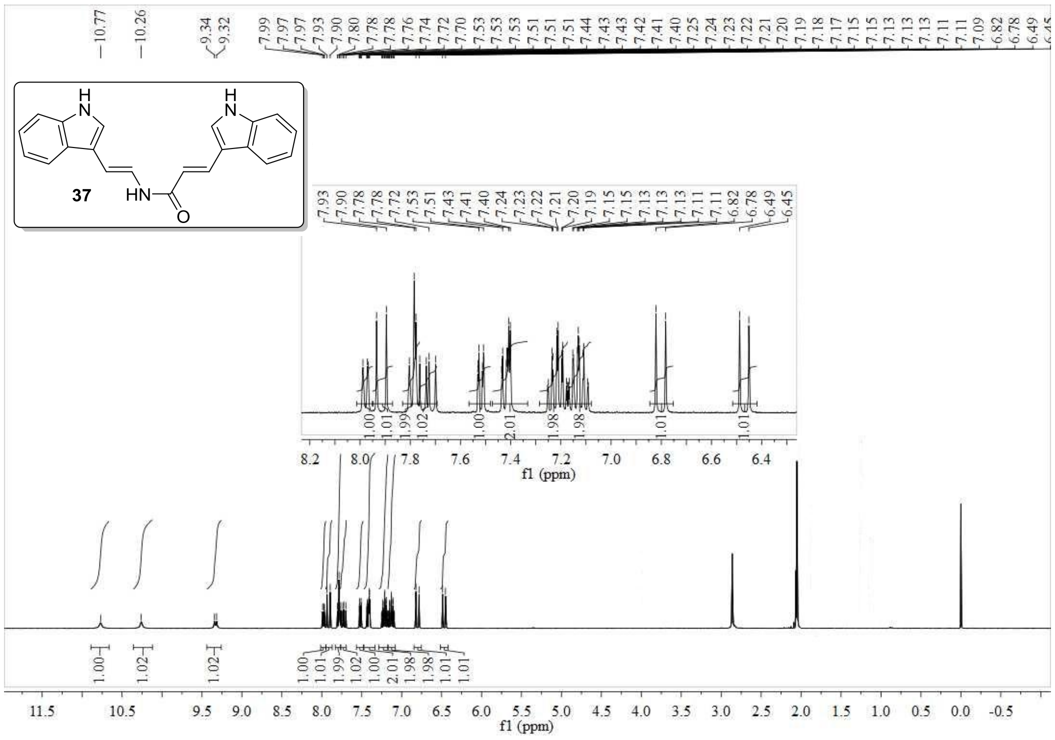

Supplementary Figure 71. ^{1}H NMR spectrum of chondriamide A (37)

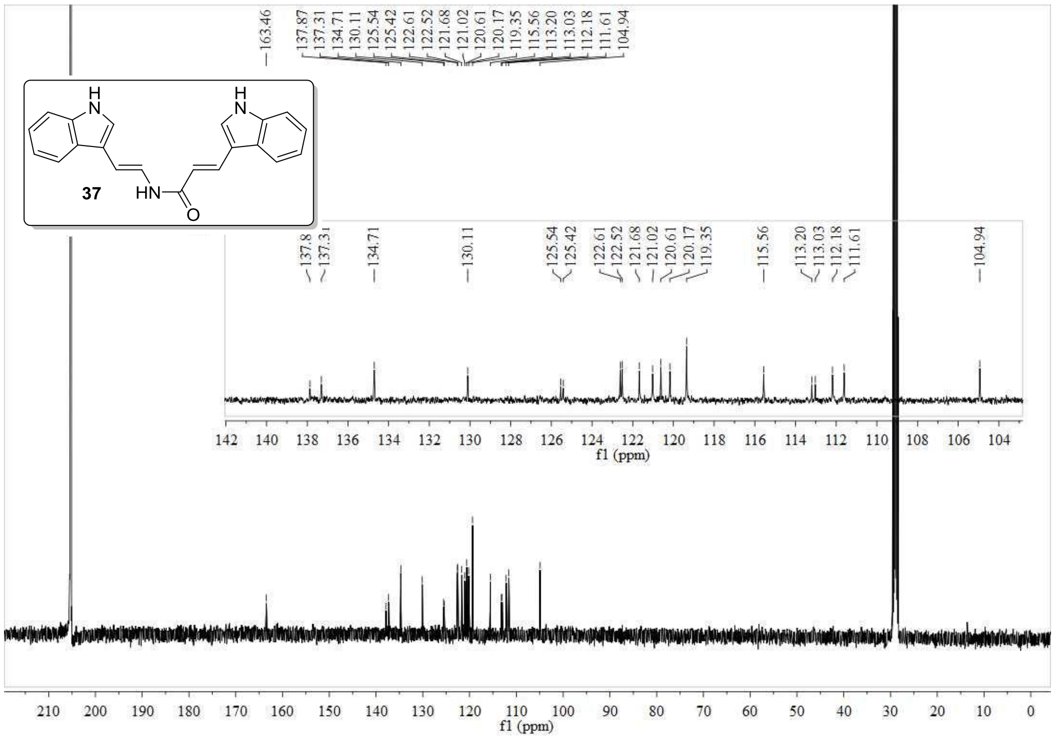

Supplementary Figure 72. ^{13}C NMR spectrum of chondriamide A (37)

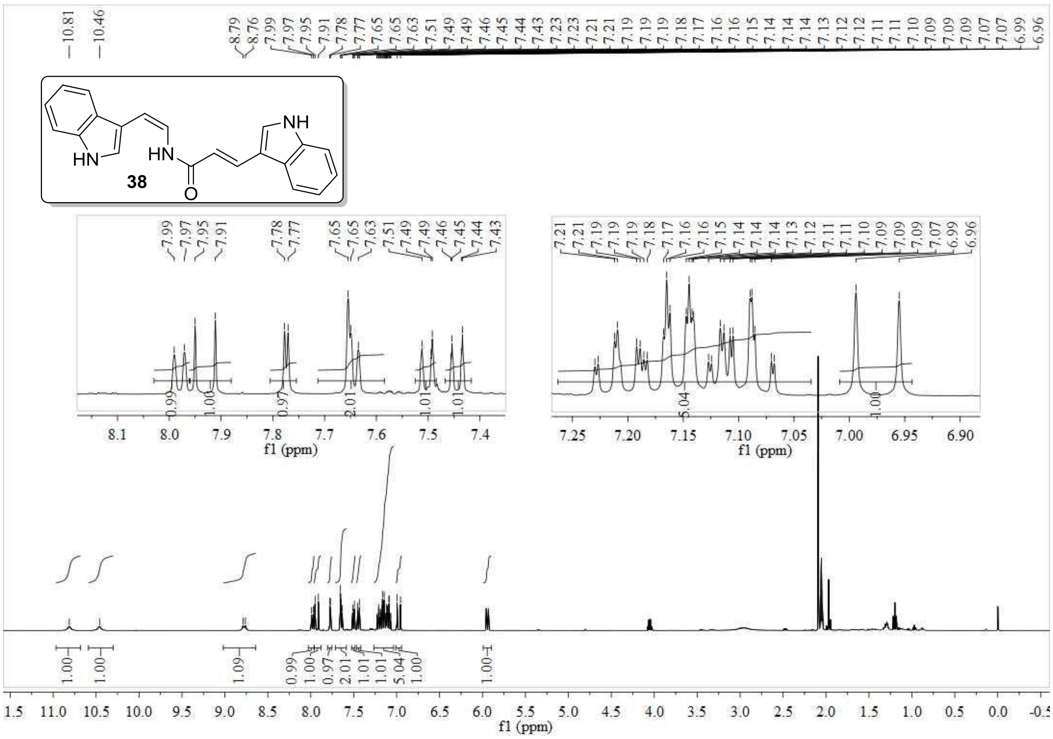

Supplementary Figure 73. ^{1}H NMR spectrum of chondriamide C (38)

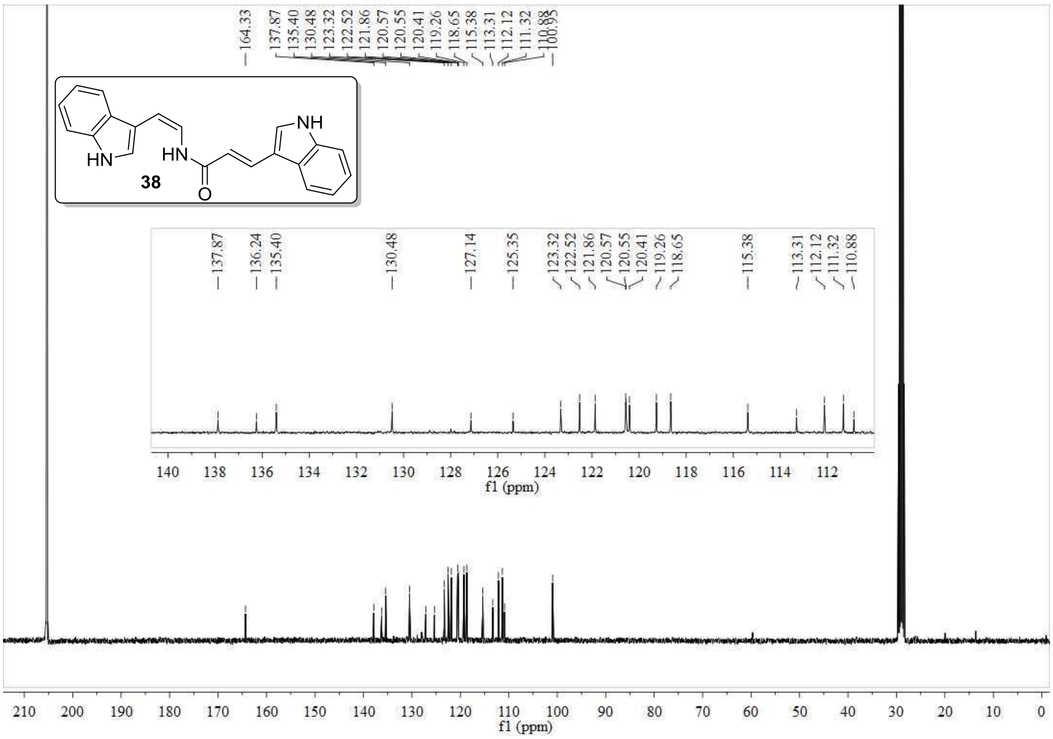

Supplementary Figure 74. ^{13}C NMR spectrum of chondriamide C (38)

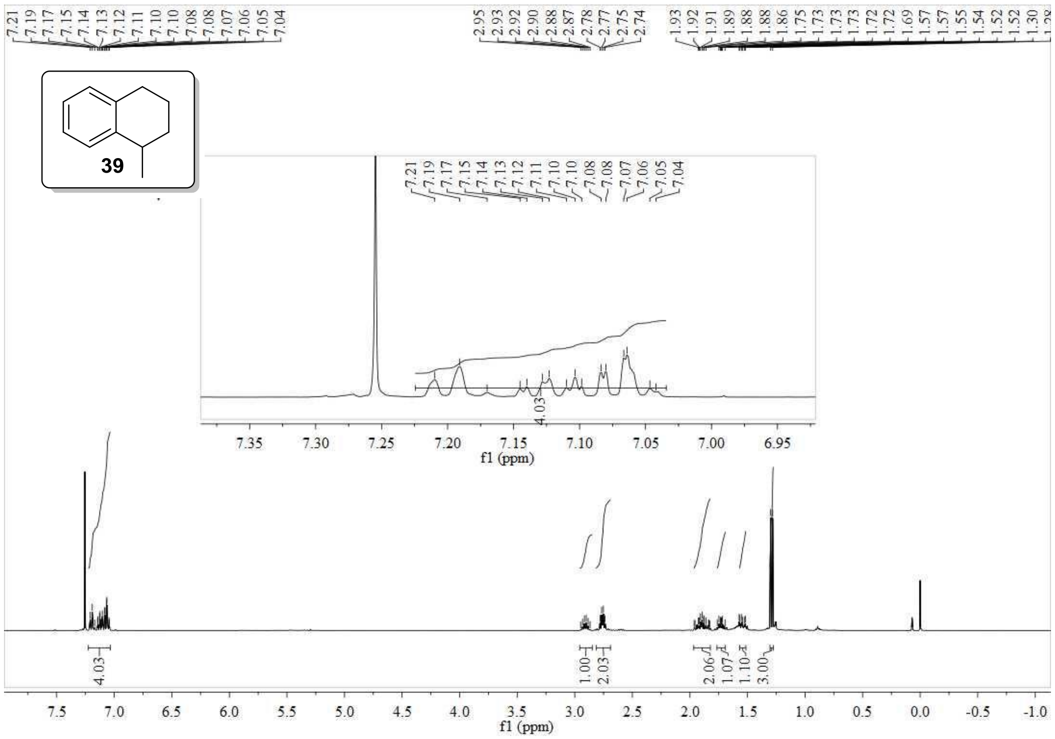

Supplementary Figure 75. ^{1}H NMR spectrum of 1-methyl-1,2,3,4-tetrahydronaphthalene (39)

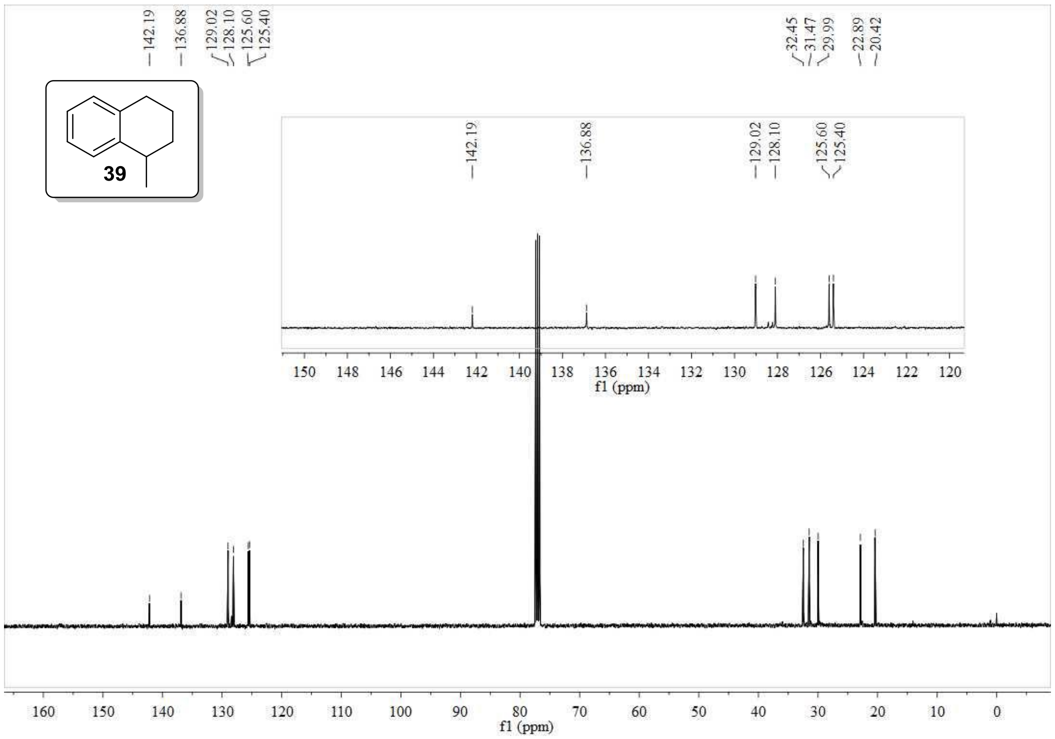

Supplementary Figure 76. ^{13}C NMR spectrum of 1-methyl-1,2,3,4-tetrahydronaphthalene (39)

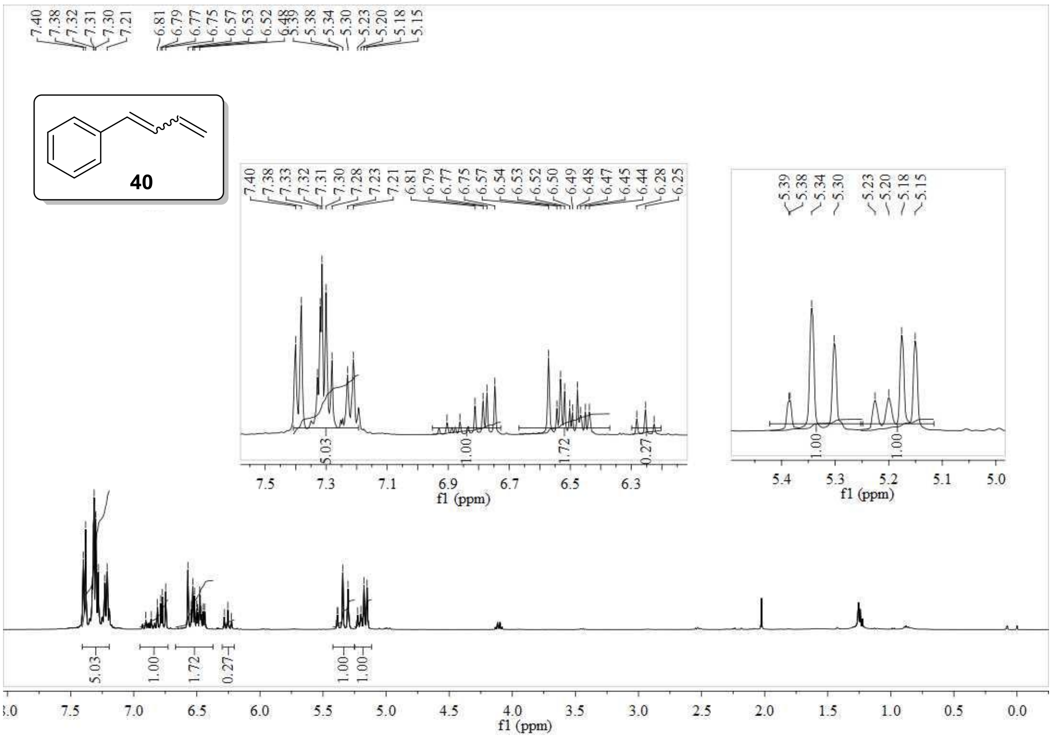

Supplementary Figure 77. ^{1}H NMR spectrum of crude products 40

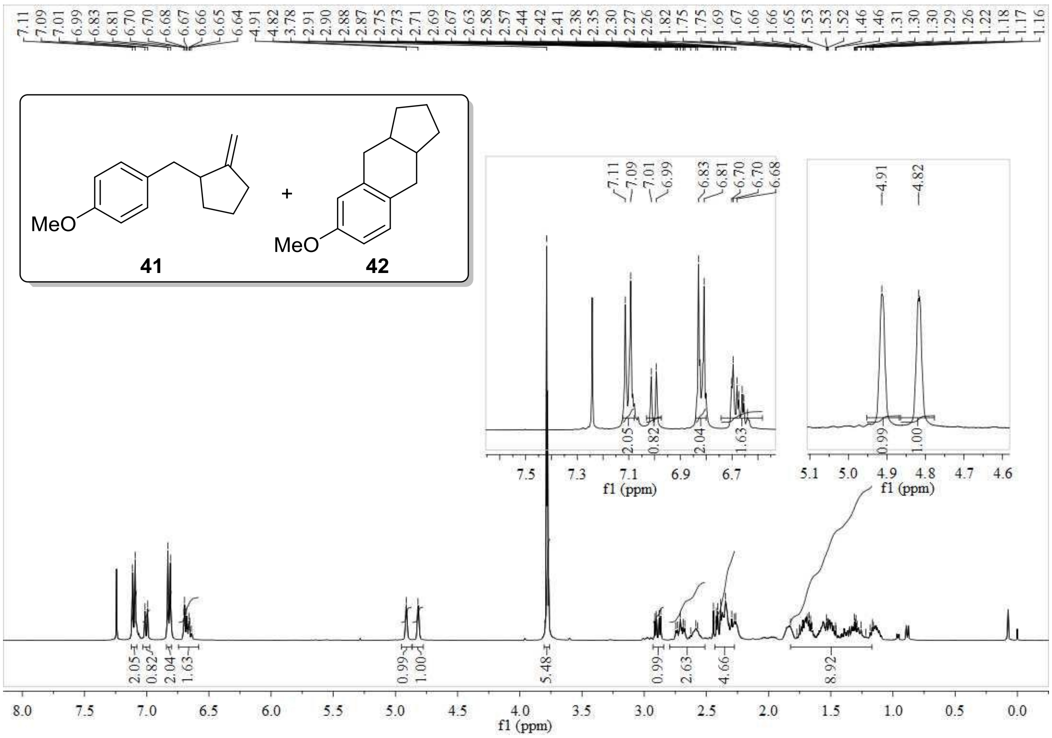

Supplementary Figure 78. ^{1}H NMR spectrum of crude products from S-9

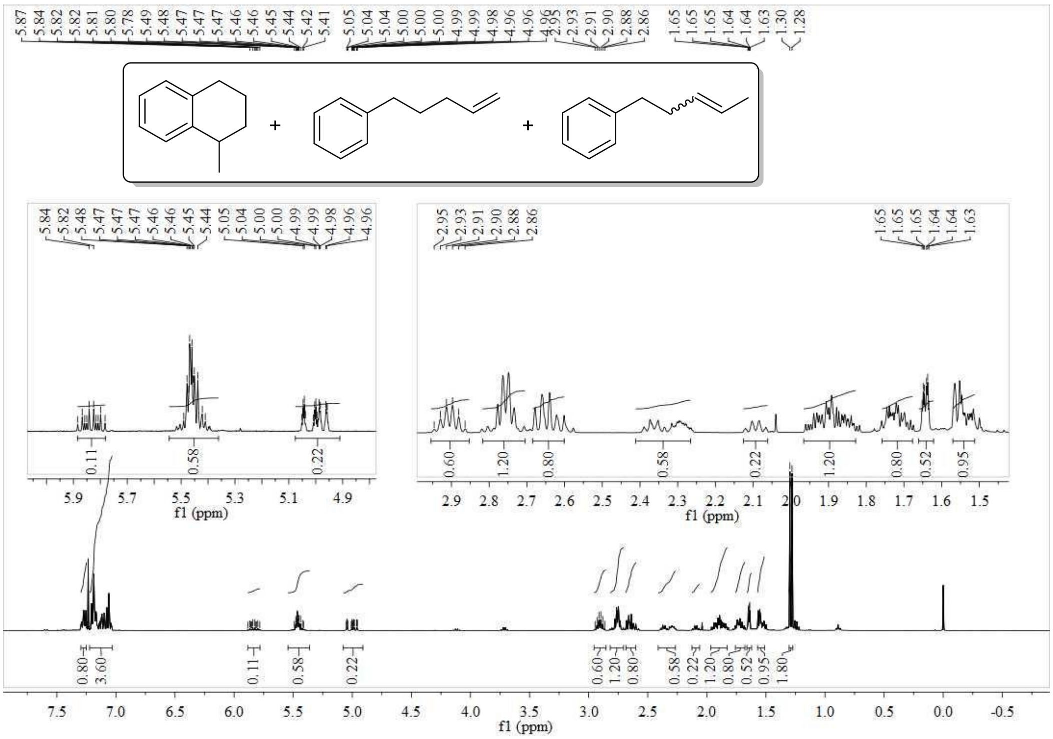

Supplementary Figure 79. ^{1}H NMR spectrum of crude products from S-7

Supplementary Table 1. Control experiments[a]

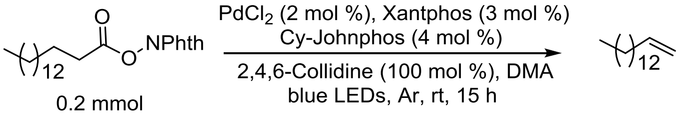

entry	variations from optimal conditions	yield (%)
1	none	93
2	without Cy-Johnphos	44
3	Without Xantphos	trace
4	without irradiation	0
5	without 2,4,6-collidine	51

[a]Reaction conditions: palmitic acid-derived redox-active ester (0.2 mmol), $PdCl_2$ (2 mol %), Xantphos (3 mol %), Cy-Johnphos (4 mol %), 2,4,6-collidine (100 mol %), DMA (2 mL), irradiated with blue LEDs at room temperature for 15 h under an Ar atmosphere. Yield determined by GC analysis using biphenyl as an internal standard.

Supplementary Table 2. Screening of different bases[a]

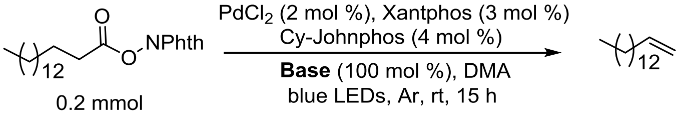

entry	base	yield (%)
1	none	51
2	K_2HPO_4	15
3	K_2CO_3	30
4	NaOAc	trace
5	DMAP	43
6	Et_3N	40
7	2,6-lutidine	64
8	Cy_2NH	66
9	2,4,6-Collidine	93

[a]Reaction conditions: palmitic acid-derived redox-active ester (0.2 mmol), $PdCl_2$ (2 mol %), Xantphos (3 mol %), Cy-Johnphos (4 mol %), base (100 mol %), DMA (2 mL), irradiated with blue LEDs at room temperature for 15 h under an Ar atmosphere. Yield determined by GC analysis using biphenyl as an internal standard.

Supplementary Table 3. Screening of different bidentate ligands[a]

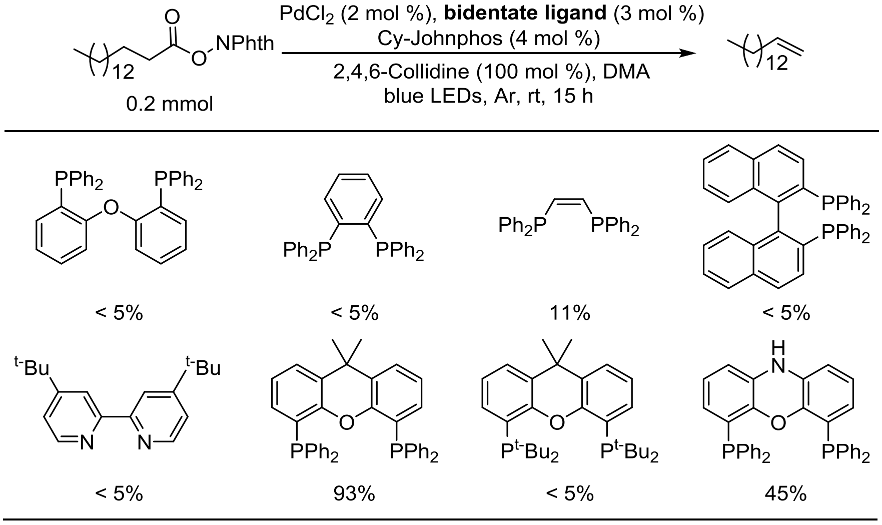

[a]Reaction conditions: palmitic acid-derived redox-active ester (0.2 mmol), $PdCl_2$ (2 mol %), bidentate ligand (3 mol %), Cy-Johnphos (4 mol %), 2,4,6-collidine (100 mol %), DMA (2 mL), irradiated with blue LEDs at room temperature for 15 h under an Ar atmosphere. Yield determined by GC analysis using biphenyl as an internal standard.

Supplementary Table 4. Screening of different monodentate ligands[a]

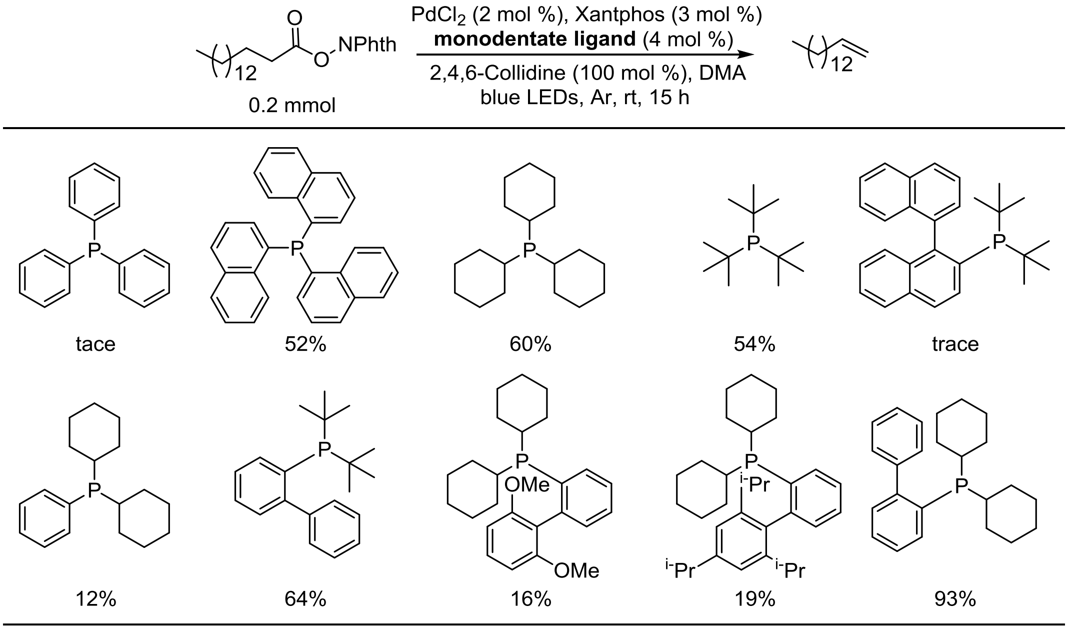

[a]Reaction conditions: palmitic acid-derived redox-active ester (0.2 mmol), $PdCl_2$ (2 mol %), Xantphos (3 mol %), monodentate ligand (4 mol %), 2,4,6-collidine (100 mol %), DMA (2 mL), irradiated with blue LEDs at room temperature for 15 h under an Ar atmosphere. Yield determined by GC analysis using biphenyl as an internal standard.

Supplementary Table 5. Study the effect of monodentate ligands on reaction selectivity[a]

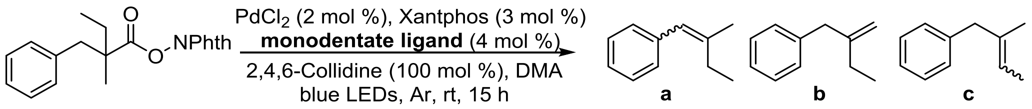

Entry	**monodentate ligand**	Yield (%)	**a / b / c**
1	Cy-Johnphos	50	1 / 2.8 / 2.1
2	PCy_3	72	1 / 3.1 / 2.5
3	P*t*Bu_3	60	1 / 3.2 / 2.3
4	Johnphos	20	1 / 2.7 / 2.3
5	$(Ad)_2PBu$	trace	-
6	Davephos	trace	-
7	S-phos	trace	-

[a]Reaction conditions: palmitic acid-derived redox-active ester (0.2 mmol), $PdCl_2$ (2 mol %), Xantphos (3 mol %), monodentate ligand (4 mol %), 2,4,6-collidine (100 mol %), DMA (2 mL), irradiated with blue LEDs at room temperature for 15 h under an Ar atmosphere. Yield and the ratio of isomers determined by ^1H NMR analysis using biphenyl as an internal standard.

Supplementary Methods

General Information

All reactions were carried out in oven-dried Schlenk tubes under argon atmosphere (purity≥99.999%) unless otherwise mentioned. Commercial reagents were purchased from Adamas-beta, TCI and Aldrich. Organic solutions were concentrated under reduced pressure on Buchi rotary evaporator. Flash column chromatographic purification of products was accomplished using forced-flow chromatography on Silica Gel (200-300 mesh).

^{1}H-NMR and ^{13}C-NMR spectra were recorded on a Bruker Avance 400 spectrometer at ambient temperature. Data for ^{1}H-NMR are reported as follows: chemical shift (ppm, scale), multiplicity (s = singlet, d = doublet, t = triplet, q = quartet, m = multiplet and/or multiplet resonances, br = broad), coupling constant (Hz), and integration. Data for ^{13}C-NMR are reported in terms of chemical shift (ppm, scale), multiplicity, and coupling constant (Hz). HRMS analysis was performed on Finnigan LCQ advantage Max Series MS System. ESI-mass data were acquired using a Thermo LTQ Orbitrap XL Instrument equipped with an ESI source and controlled by Xcalibur software. The blue LED lamps (λ_{max} =440 nm) were purchased from Kessil (PR160-440 nm). The decarboxylative desaturation reactions were conducted in front of a cooling fan in a room of constant temperature (t =25 ± 3 ℃).

Procedure for the Synthesis of Redox Active Esters

The redox active esters can be synthesized by the condensation of corresponding carboxylic acids with *N*-hydroxyphthalimide[1].

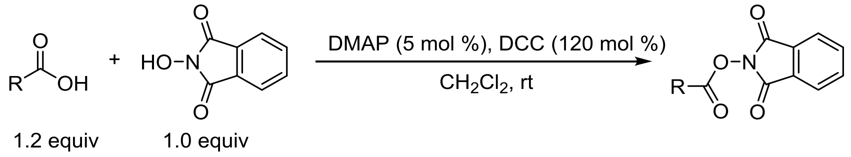

The corresponding alkyl carboxylic acids or *N*-protected amino acids (12 mmol, 1.2 equiv), *N*-hydroxyphthalimide (1.63 g, 10 mmol, 1.0 equiv), and 4-dimethylaminopyridine (61 mg, 0.5 mmol, 5 mol %) were mixed in a flask with a magnetic stirring bar. 40 mL dry CH_2Cl_2 was added, then a solution of *N,N'*-dicyclohexylcarbodiimide (2.48 g, 12 mmol, 120 mol %) in CH_2Cl_2 (15 mL) was added slowly at room temperature. The reaction mixture was stirred at room temperature for 1

h. After *N*-hydroxyphthalimide was completely converted, the white precipitate was filtered off and the solution was concentrated under vacuum. Corresponding redox active esters were purified by column chromatography on silica gel (CH_2Cl_2 or petroleum ether/ethyl acetate as eluent).

Experimental Procedures for Irradiation-Induced Palladium-Catalyzed Decarboxylative Desaturation

General Procedure A: Redox active ester (1.0 equiv, 0.2 mmol) (if solid), $PdCl_2$ (2 mol %, 0.7 mg), Xantphos (3 mol %, 3.5 mg) and Cy-Johnphos (4 mol %, 2.8 mg) were placed in a transparent Schlenk tube equipped with a stirring bar. The tube was evacuated and filled with argon (three times). To these solids, redox active ester (1.0 equiv, 0.2 mmol) (if liquid), anhydrous *N*,*N*-dimethylacetamide (DMA, 2.0 mL) and 2,4,6-collidine (1.0 equiv, 0.2 mmol, 24.2 mg) was added via a gastight syringe under argon atmosphere. The reaction mixture was stirred under the irradiation of blue LEDs (distance app. 3.0 cm from the bulb) at room temperature for 15 h. After 15 h, the mixture was quenched with saturated NaCl solution and extracted with ethyl acetate (3 x 10 mL). The organic layers were combined and concentrated under vacuo. The product was purified by flash column chromatography on silica gel with petroleum ether or a mixture of petroleum ether and ethyl acetate as eluent.

General Procedure B: Redox active ester (1.0 equiv, 0.2 mmol) (if solid), $PdCl_2$ (5 mol %, 1.8 mg), Xantphos (6 mol %, 6.9 mg) and Cy-Johnphos (10 mol %, 7.0 mg) were placed in a transparent Schlenk tube equipped with a stirring bar. The tube was evacuated and filled with argon (three times). To these solids, redox active ester (1.0 equiv, 0.2 mmol) (if liquid), anhydrous *N*,*N*-dimethylacetamide (DMA, 2.0 mL) and 2,4,6-collidine (1.0 equiv, 0.2 mmol, 24.2 mg) was added via a gastight syringe under argon atmosphere. The reaction mixture was stirred under the irradiation of blue LEDs (distance app. 3.0 cm from the bulb) at room temperature for 15 h. After 15 h, the mixture was quenched with saturated NaCl solution and extracted with ethyl acetate (3 x 10 mL). The organic layers were combined and concentrated under vacuo. The product was purified by flash column chromatography on silica gel with petroleum ether or a mixture of petroleum ether and ethyl acetate as eluent.

Characterization Data for Products in Figure 2 and Figure 3

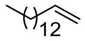

1-Pentadecene (2): Following the general procedure A, obtained in 93% yield as a colorless liquid (39.1 mg, eluent: petroleum ether). The compound data was in agreement with the literature[2].

^{1}H NMR (400 MHz, $CDCl_3$) δ 5.81 (ddt, *J* = 16.9, 10.2, 6.7 Hz, 1H), 5.07 – 4.84 (m, 2H), 2.09 – 1.98 (m, 2H), 1.40 – 1.24 (m, 22H), 0.88 (t, *J* = 6.8 Hz, 3H). ^{13}C NMR (101 MHz, $CDCl_3$) δ 139.3, 114.1, 33.8, 31.9, 29.7, 29.7, 29.7, 29.7, 29.6, 29.5, 29.4, 29.2, 29.0, 22.7, 14.1.

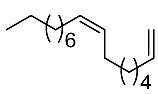

(*Z*)-heptadeca-1,8-diene (4): Following the general procedure A, obtained in 80% yield as a colorless liquid (37.8 mg, eluent: petroleum ether). The compound data was in agreement with the literature[3].

^{1}H NMR (400 MHz, CD_2Cl_2) δ 5.81 (ddt, *J* = 16.9, 10.2, 6.7 Hz, 1H), 5.38 – 5.33 (m, 2H), 5.04 – 4.90 (m, 2H), 2.08 – 1.98 (m, 6H), 1.41 – 1.25 (m, 18H), 0.88 (t, *J* = 6.9 Hz, 3H). ^{13}C NMR (101 MHz, CD_2Cl_2) δ 139.2, 130.0, 129.8, 114.2, 33.8, 31.9, 29.8, 29.6, 29.5, 29.3, 28.9, 28.8, 27.2, 27.1, 22.7, 14.1. (one carbon signal is overlapped)

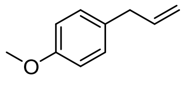

1-allyl-4-methoxybenzene (5): Following the general procedure B, obtained in 65% yield as a colorless liquid (19.3 mg, eluent: petroleum ether). The compound data was in agreement with the literature[4].

^{1}H NMR (400 MHz, $CDCl_3$) δ 7.11 (d, *J* = 8.5 Hz, 2H), 6.84 (d, *J* = 8.6 Hz, 2H), 5.95 (ddt, *J* = 16.9, 10.2, 6.7 Hz, 1H), 5.11 – 5.00 (m, 2H), 3.79 (s, 3H), 3.33 (d, *J* = 6.7 Hz, 2H). ^{13}C NMR (101 MHz, $CDCl_3$) δ 157.9, 137.9, 132.1, 129.5, 115.4, 113.8, 55.3, 39.3.

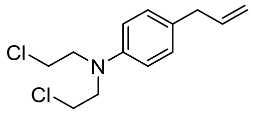

4-allyl-*N,N*-bis(2-chloroethyl)aniline (6): Following the general procedure B, obtained in 54% yield as a colorless liquid (27.9 mg, eluent: petroleum ether/ethyl acetate = 50/1). ^{1}H NMR (400 MHz, $CDCl_3$) δ 7.09 (d, *J* = 8.8 Hz, 2H), 6.65 (d, *J* = 8.7 Hz, 2H), 5.94 (ddt, *J* = 16.8, 10.0, 6.7 Hz, 1H), 5.15 – 4.96 (m, 2H), 3.74 – 3.67 (m, 4H), 3.65 – 3.59 (m, 4H), 3.30 (d, *J* = 6.7 Hz, 2H). ^{13}C NMR (101 MHz, $CDCl_3$) δ 144.2, 137.9, 129.8, 129.5, 115.4, 112.4, 53.7, 40.4, 39.2. HRMS (ESI): $[M+H]^+$ Calcd. for $C_{13}H_{18}Cl_2N$, 258.0816; found: 258.0811.

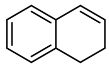

1,2-dihydronaphthalene (7): Following the general procedure A, obtained in 76% yield as a colorless liquid (19.8 mg, eluent: petroleum ether). The compound data was in agreement with the literature[5].

^{1}H NMR (400 MHz, $CDCl_3$) δ 7.17 – 7.08 (m, 3H), 7.01 (d, *J* = 6.9 Hz, 1H), 6.46 (dd, *J* = 9.6, 1.4 Hz, 1H), 6.06 – 5.99 (m, 1H), 2.80 (t, *J* = 8.2 Hz, 2H), 2.36 – 2.26 (m, 2H). ^{13}C NMR (101 MHz, $CDCl_3$) δ 135.4, 134.1, 128.7, 127.7, 127.5, 126.8, 126.4, 125.9, 27.5, 23.2.

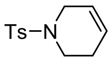

1-tosyl-1,2,3,6-tetrahydropyridine (8): Following the general procedure B, obtained in 82% yield as a white solid (38.9 mg, eluent: petroleum ether/ethyl acetate = 10/1). The compound data was in agreement with the literature[6].

^{1}H NMR (400 MHz, $CDCl_3$) δ 7.67 (d, *J* = 8.3 Hz, 2H), 7.32 (d, *J* = 8.0 Hz, 2H), 5.78 – 5.72 (m, 1H), 5.64 – 5.58 (m, 1H), 3.63 – 3.53 (m, 2H), 3.17 (t, *J* = 5.7 Hz, 2H), 2.43 (s, 3H), 2.25 – 2.18 (m, 2H). ^{13}C NMR (101 MHz, $CDCl_3$) δ 143.5, 133.3, 129.6, 127.7, 125.1, 122.7, 44.8, 42.7, 25.3, 21.6.

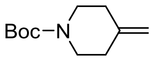

***tert*-butyl 4-methylenepiperidine-1-carboxylate (9):** Following the general procedure A, obtained in 50% yield as a colorless liquid (19.7 mg, eluent: petroleum ether/ethyl acetate = 5/1). The compound data was in agreement with the literature[7].

^{1}H NMR (400 MHz, $CDCl_3$) δ 4.74 (s, 2H), 3.46 – 3.39 (m, 4H), 2.21 – 2.08 (m, 4H), 1.47 (s, 9H). ^{13}C NMR (101 MHz, $CDCl_3$) δ 154.7, 145.4, 109.1, 79.5, 45.2, 34.5, 28.5.

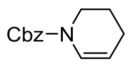

Benzyl 3,4-dihydropyridine-1(2*H*)-carboxylate (10): Following the general procedure B, obtained in 62% yield as a colorless liquid (26.9 mg, eluent: petroleum ether/ethyl acetate = 10/1). The compound data was in agreement with the literature[8].

^{1}H NMR (400 MHz, Acetone) (46:54 mixture of rotamers) δ 7.45 – 7.29 (m, 5H), 6.83 (d, J = 8.5 Hz, 0.46H), 6.79 (d, J = 8.4 Hz, 0.54H), 5.16 (s, 2H), 5.00 – 4.91 (m, 0.46H), 4.90 – 4.79 (m, 0.54H), 3.65 – 3.52 (m, 2H), 2.08 – 1.96 (m, 2H). 1.82 – 1.76 (m, 2H). ^{13}C NMR (101 MHz, Acetone) (rotamers observed) δ 154.0, 153.5, 137.9, 129.4, 129.0, 128.9, 126.2, 125.8, 107.2, 106.8, 67.9, 67.8, 43.2, 42.9, 22.5, 22.2, 22.0.

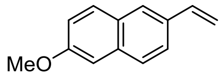

2-methoxy-6-vinylnaphthalene (11): Following the general procedure A, obtained in 96% yield as a white solid (35.4 mg, eluent: petroleum ether). The compound data was in agreement with the literature[9].

^{1}H NMR (400 MHz, $CDCl_3$) δ 7.71 – 7.65 (m, 3H), 7.59 (d, J = 8.8 Hz, 1H), 7.15 – 7.04 (m, 2H), 6.83 (dd, J = 17.6, 10.9 Hz, 1H), 5.80 (d, J = 17.6 Hz, 1H), 5.26 (d, J = 10.9 Hz, 1H), 3.89 (s, 3H). ^{13}C NMR (101 MHz, $CDCl_3$) δ 157.8, 136.9, 134.3, 133.0, 129.5, 129.0, 127.0, 126.2, 123.8, 118.9, 113.1, 105.9, 55.3.

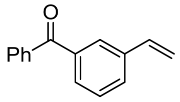

Phenyl(3-vinylphenyl)methanone (12): Following the general procedure A, obtained in 94% yield as a colorless oil (39.1 mg, eluent: petroleum ether). The compound data was in agreement with the literature[10].

^{1}H NMR (400 MHz, $CDCl_3$) δ 7.85 – 7.78 (m, 3H), 7.68 – 7.55 (m, 3H), 7.52 – 7.40 (m, 3H), 6.76 (dd, J = 17.6, 10.9 Hz, 1H), 5.80 (d, J = 17.6 Hz, 1H), 5.32 (d, J = 10.9 Hz, 1H). ^{13}C NMR (101 MHz, $CDCl_3$) δ 196.6, 138.0, 137.8, 137.6, 136.0, 132.5, 130.1, 129.9, 129.4, 128.5, 128.3, 127.7, 115.3.

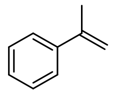

Prop-1-en-2-ylbenzene (13): Following the general procedure A, obtained in 94% yield as a colorless liquid (22.2 mg, eluent: petroleum ether). The compound data was in agreement with the literature[11].

^{1}H NMR (400 MHz, $CDCl_3$) δ 7.50 – 7.45 (m, 2H), 7.36 – 7.30 (m, 2H), 7.29 – 7.25 (m, 1H), 5.37 (s, 1H), 5.08 (s, 1H), 2.16 (s, 3H). ^{13}C NMR (101 MHz, $CDCl_3$) δ 143.3, 141.2, 128.2, 127.4, 125.5, 112.4, 21.8.

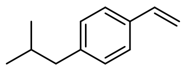

1-isobutyl-4-vinylbenzene (14): Following the general procedure A, obtained in 96% yield as a colorless oil (30.7 mg, eluent: petroleum ether). The compound data was in agreement with the literature[12].

^{1}H NMR (400 MHz, $CDCl_3$) δ 7.32 (d, *J* = 8.1 Hz, 2H), 7.10 (d, *J* = 8.0 Hz, 2H), 6.69 (dd, *J* = 17.6, 10.9 Hz, 1H), 5.70 (dd, *J* = 17.6, 1.0 Hz, 1H), 5.18 (dd, *J* = 10.9, 1.0 Hz, 1H), 2.45 (d, *J* = 7.2 Hz, 2H), 1.91 – 1.79 (m, 1H), 0.90 (d, *J* = 6.6 Hz, 6H). ^{13}C NMR (101 MHz, $CDCl_3$) δ 141.5, 136.8, 135.0, 129.3, 126.0, 112.8, 45.2, 30.2, 22.4.

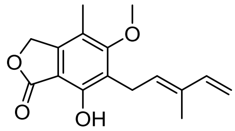

(*E*)-7-hydroxy-5-methoxy-4-methyl-6-(3-methylpenta-2,4-dien-1-yl)isobenzofuran-1(3*H*)-one (18): Following the general procedure B, using 0.4 mmol 2,4,6-collidine, obtained in 35% yield as a white solid (19.2 mg, eluent: petroleum ether/ethyl acetate = 4/1).

^{1}H NMR (400 MHz, $CDCl_3$) δ 7.70 (s, 1H), 6.33 (dd, *J* = 17.4, 10.7 Hz, 1H), 5.54 (t, *J* = 7.1 Hz, 1H), 5.21 (s, 2H), 5.13 (d, *J* = 17.4 Hz, 1H), 4.94 (d, *J* = 10.7 Hz, 1H), 3.77 (s, 3H), 3.54 (d, *J* = 7.2 Hz, 2H), 2.15 (s, 3H), 1.92 (d, *J* = 0.8 Hz, 3H). ^{13}C NMR (101 MHz, $CDCl_3$) δ 172.9, 163.7, 153.6, 144.2, 141.3, 134.5, 130.1, 121.5, 116.8, 111.2, 106.4, 70.1, 61.0, 22.8, 11.8, 11.6. HRMS (ESI): $[M+Na]^+$ Calcd. for $C_{16}H_{18}O_4Na$, 297.1097; found: 297.1089.

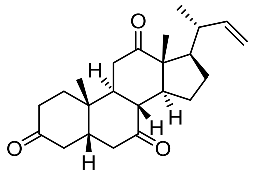

(5*S*,8*R*,9*S*,10*S*,13*R*,14*S*,17*R*)-17-((*R*)-but-3-en-2-yl)-10,13-dimethyldecahydro-1*H*-cyclopenta[a]phenanthrene-3,7,12(2*H*,4*H*,8*H*)-trione (19): Following the general procedure A, obtained in 77% yield as a white solid (54.9 mg, eluent: petroleum ether/ethyl acetate = 3/1). The compound data was in agreement with the literature[13].

^{1}H NMR (400 MHz, $CDCl_3$) δ 5.72 (ddd, *J* = 17.1, 10.1, 8.3 Hz, 1H), 5.03 – 4.80 (m, 2H), 2.96 – 2.81 (m, 3H), 2.38 – 1.96 (m, 12H), 1.89 – 1.81 (m, 2H), 1.67 – 1.61 (m, 1H), 1.41 (s, 3H), 1.33 – 1.22 (m, 2H), 1.09 (s, 3H), 0.97 (d, *J* = 6.6 Hz, 3H). ^{13}C NMR (101 MHz, $CDCl_3$) δ 211.9, 209.1, 208.8, 144.1, 112.8, 56.7, 51.5, 48.9, 46.9, 45.3, 45.2, 45.0, 42.8, 41.4, 38.6, 36.5, 36.0, 35.3, 27.4, 25.0, 21.9, 20.8, 12.2.

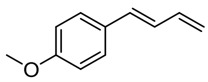

(*E*)-1-(buta-1,3-dien-1-yl)-4-methoxybenzene (20-*E*):Following the general procedure B, obtained in 66% yield as a white solid (21.1 mg, eluent: petroleum ether). The compound data was in agreement with the literature[14].

^{1}H NMR (400 MHz, $CDCl_3$) δ 7.34 (d, *J* = 8.7 Hz, 2H), 6.86 (d, *J* = 8.8 Hz, 2H), 6.67 (dd, *J* = 15.4, 10.5 Hz, 1H), 6.55 – 6.42 (m, 2H), 5.34 – 5.22 (m, 1H), 5.14 – 5.09 (m, 1H), 3.81 (s, 3H). ^{13}C NMR (101 MHz, $CDCl_3$) δ 159.3, 137.4, 132.4, 129.9, 127.6, 116.4, 114.1, 55.3. (one carbon signal is overlapped)

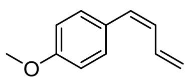

(*Z*)-1-(buta-1,3-dien-1-yl)-4-methoxybenzene (20-*Z*): Following the general procedure B, obtained in 25% yield as a colorless liquid (8.0 mg, eluent: petroleum ether). The compound data was in agreement with the literature[15].

^{1}H NMR (400 MHz, $CDCl_3$) δ 7.27 (d, *J* = 8.7 Hz, 2H), 6.98 – 6.78 (m, 3H), 6.40 (d, *J* = 11.5 Hz, 1H), 6.19 (t, *J* = 11.3 Hz, 1H), 5.39 – 5.31 (m, 1H), 5.20 (d, *J* = 10.1 Hz, 1H), 3.82 (s, 3H). ^{13}C NMR (101 MHz, $CDCl_3$) δ 158.7, 133.3, 130.3, 130.0, 130.0, 129.4, 119.0, 113.7, 55.3.

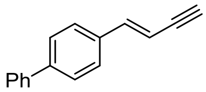

(*E*)-4-(but-1-en-3-yn-1-yl)-1,1'-biphenyl (21-*E*): Following the general procedure B, obtained in 58% yield as a solid (23.7 mg, eluent: petroleum ether).

^{1}H NMR (400 MHz, $CDCl_3$) δ 7.59 – 7.55 (m, 4H), 7.45 – 7.41 (m, 4H), 7.34 (t, *J* = 7.3 Hz, 1H), 7.07 (d, *J* = 16.3 Hz, 1H), 6.16 (dd, *J* = 16.3, 2.3 Hz, 1H), 3.07 (d, *J* = 2.2 Hz, 1H). ^{13}C NMR (101 MHz, $CDCl_3$) δ 142.7, 141.7, 140.4, 134.9, 128.9, 127.6, 127.4, 127.0, 126.9, 107.0, 83.1, 79.5. HRMS (ESI): $[M+H]^+$ Calcd. for $C_{16}H_{13}$, 205.1012; found: 205.1016.

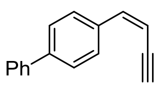

(*Z*)-4-(but-1-en-3-yn-1-yl)-1,1'-biphenyl (21-*Z*): Following the general procedure B, obtained in 28% yield as a solid (11.4 mg, eluent: petroleum ether).

^{1}H NMR (400 MHz, $CDCl_3$) δ 7.95 (d, *J* = 8.3 Hz, 2H), 7.67 – 7.54 (m, 4H), 7.44 (t, *J* = 7.5 Hz, 2H), 7.39 – 7.32 (m, 1H), 6.75 (d, *J* = 12.1 Hz, 1H), 5.70 (dd, *J* = 12.1, 2.7 Hz, 1H), 3.40 (dd, *J* = 2.7, 0.9 Hz, 1H). ^{13}C NMR (101 MHz, $CDCl_3$) δ 141.4, 140.6, 140.1, 135.1, 129.2, 128.9, 127.6, 127.1, 127.0, 106.3, 84.5, 82.1. HRMS (ESI): $[M+H]^+$ Calcd. for $C_{16}H_{13}$, 205.1012; found: 205.1010.

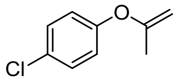

1-chloro-4-(prop-1-en-2-yloxy)benzene (22): Following the general procedure B, obtained in 78% yield as a colorless liquid (26.3 mg, eluent: petroleum ether).

^{1}H NMR (400 MHz, $CDCl_3$) δ 7.29 (d, *J* = 8.8 Hz, 2H), 6.97 (d, *J* = 8.8 Hz, 2H), 4.19 (s, 1H), 3.96 (d, *J* = 1.4 Hz, 1H), 1.97 (s, 3H). ^{13}C NMR (101 MHz, $CDCl_3$) δ 159.4, 153.9, 129.6, 129.0, 122.0, 90.2, 19.9. HRMS (ESI): $[M+H]^+$ Calcd. for $C_9H_{10}ClO$, 169.0415; found: 169.0410.

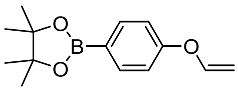

4,4,5,5-tetramethyl-2-(4-(vinyloxy)phenyl)-1,3,2-dioxaborolane (23): Following the

general procedure B, obtained in 72% yield as a colorless oil (35.4 mg, eluent: petroleum ether/ethyl acetate = 20/1).

^{1}H NMR (400 MHz, $CDCl_3$) δ 7.78 (d, *J* = 8.6 Hz, 2H), 6.99 (d, *J* = 8.6 Hz, 2H), 6.67 (dd, *J* = 13.7, 6.1 Hz, 1H), 4.82 (dd, *J* = 13.7, 1.7 Hz, 1H), 4.48 (dd, *J* = 6.1, 1.7 Hz, 1H), 1.34 (s, 12H). ^{13}C NMR (101 MHz, $CDCl_3$) δ 159.3, 147.5, 136.6, 116.0, 95.9, 83.7, 24.9. The carbon directly attached to the boron atom was not detected due to quadrupolar broadening. HRMS (ESI): $[M+H]^+$ Calcd. for $C_{14}H_{20}O_3B$, 247.1500; found: 247.1491.

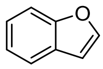

Benzofuran (24): Following the general procedure B, obtained in 82% yield as a colorless liquid (19.4 mg, eluent: petroleum ether/ethyl acetate = 50/1). The compound data was in agreement with the literature[16].

^{1}H NMR (400 MHz, $CDCl_3$) δ7.62 – 7.58 (m, 2H), 7.51 (d, *J* = 8.3 Hz, 1H), 7.29 (t, *J* = 7.7 Hz, 1H), 7.26 – 7.21 (m, 1H), 6.76 (d, *J* = 1.8 Hz, 1H). ^{13}C NMR (101 MHz, $CDCl_3$) δ 155.0, 144.9, 127.4, 124.2, 122.7, 121.2, 111.4, 106.6.

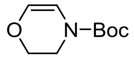

***tert*-butyl 2*H*-1,4-oxazine-4(3*H*)-carboxylate (25):** Following the general procedure B, obtained in 70% yield as a colorless liquid (25.9 mg, eluent: petroleum ether/ethyl acetate = 5/1).

^{1}H NMR (400 MHz, $CDCl_3$) (36:64 mixture of rotamers) δ 6.29 (d, *J* = 4.9 Hz, 0.36H), 6.14 (d, *J* = 5.0 Hz, 0.64H), 5.99 (d, *J* = 5.0 Hz, 0.36H), 5.86 (d, *J* = 5.0 Hz, 0.64H), 4.14 – 4.00 (m, 2H), 3.72 – 3.62 (m, 2H), 1.49 (s, 9H). ^{13}C NMR (101 MHz, $CDCl_3$) (rotamers observed) δ 151.2, 129.4, 128.1, 106.3, 106.1, 80.9, 80.8, 64.8, 64.3, 42.4, 40.9, 28.3. HRMS (ESI): $[M+H]^+$ Calcd. for $C_9H_{16}O_3N$, 186.1125; found: 186.1118.

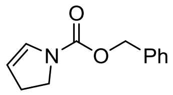

Benzyl 2,3-dihydro-1*H*-pyrrole-1-carboxylate (26): Following the general procedure B, obtained in 75% yield as a colorless oil (30.5 mg, eluent: petroleum ether/ethyl acetate = 10/1). The compound data was in agreement with the literature[17].

^{1}H NMR (400 MHz, Acetone) δ 7.62 – 7.14 (m, 5H), 6.61 – 6.54 (m, 1H), 5.25 – 4.95 (m, 3H), 3.83 – 3.59 (m, 2H), 2.82 – 2.48 (m, 2H). ^{13}C NMR (101 MHz, Acetone) (rotamers observed) δ 152.3, 151.6, 137.1, 129.5, 128.9, 128.4, 127.9, 127.8, 108.5, 108.4, 66.4, 66.3, 45.0, 44.8, 28.2.

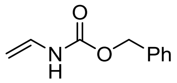

Benzyl vinylcarbamate (27): Following the general procedure B, obtained in 78% yield as a white solid (27.6 mg, eluent: petroleum ether/ethyl acetate = 10/1). The compound data was in agreement with the literature[18].

^{1}H NMR (400 MHz, Acetone) δ 7.42 – 7.31 (m, 5H), 6.70 (dd, *J* = 15.9, 8.7 Hz, 1H), 5.13 (s, 2H), 4.62 (d, *J* = 15.9 Hz, 1H), 4.23 (d, *J* = 8.8 Hz, 1H), 3.27 – 3.17 (m, 1H). ^{13}C NMR (101 MHz, Acetone) δ 153.8, 136.8, 130.5, 128.4, 128.0, 127.9, 92.4, 66.2.

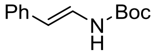

(*E*)-*tert*-butyl styrylcarbamate (28): Following the general procedure B, obtained in 95% yield as a white solid (41.6 mg, *E/Z* = 93/7, eluent: petroleum ether/ethyl acetate = 5/1). The compound data was in agreement with the literature[19].

^{1}H NMR (400 MHz, $CDCl_3$) δ 7.31 – 7.18 (m, 5H), 7.17 – 7.10 (m, 1H), 6.53 (d, *J* = 8.4 Hz, 1H), 5.90 (d, *J* = 14.6 Hz, 1H), 1.50 (s, 9H). ^{13}C NMR (101 MHz, $CDCl_3$) δ 152.7, 136.6, 128.6, 126.1, 125.2, 124.3, 109.7, 80.9, 28.3.

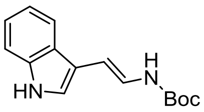

(*E*)-*tert*-butyl (2-(1*H*-indol-3-yl)vinyl)carbamate (29): Following the general procedure B, obtained in 80% yield as yellow viscous oil (41.3 mg, *E/Z* = 94/6, eluent: petroleum ether/ethyl acetate = 3/1). The compound data was in agreement with the literature[20].

^{1}H NMR (400 MHz, CD_2Cl_2) δ 8.40 (s, 1H), 7.74 (d, *J* = 7.9 Hz, 1H), 7.37 (d, *J* = 8.1 Hz, 1H), 7.26 – 7.06 (m, 4H), 6.61 (s, 1H), 6.16 (d, *J* = 14.7 Hz, 1H), 1.51 (s, 9H). ^{13}C NMR

(101 MHz, CD_2Cl_2) δ 152.8, 136.7, 125.3, 122.2, 121.8, 121.4, 119.8, 119.5, 113.1, 111.3, 102.8, 80.3, 28.1.

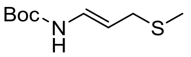

(*E*)-*tert*-butyl (3-(methylthio)prop-1-en-1-yl)carbamate (30): Following the general procedure B, obtained in 75% yield as a colorless liquid (30.5 mg, *E/Z* = 96/4, eluent: petroleum ether/ethyl acetate = 5/1).

^{1}H NMR (400 MHz, $CDCl_3$) δ 6.61 – 6.47 (m, 1H), 6.29 (br s, 1H), 4.97 – 4.88 (m, 1H), 3.08 (d, *J* = 7.6 Hz, 2H), 2.02 (s, 3H), 1.47 (s, 9H). ^{13}C NMR (101 MHz, $CDCl_3$) δ 152.6, 126.0, 105.1, 80.6, 34.0, 28.3, 14.3. HRMS (ESI): $[M+Na]^+$ Calcd. for $C_9H_{17}NO_2SNa$, 226.0872; found: 226.0865.

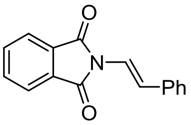

(*E*)-2-styrylisoindoline-1,3-dione (31): Following the general procedure B, obtained in 65% yield as a yellow powder (32.4 mg, *E/Z* = 95/5, eluent: petroleum ether/ethyl acetate = 5/1). The compound data was in agreement with the literature[19].

^{1}H NMR (400 MHz, $CDCl_3$) δ 7.89 (dd, *J* = 5.5, 3.0 Hz, 2H), 7.75 (dd, *J* = 5.5, 3.0 Hz, 2H), 7.65 (d, *J* = 15.2 Hz, 1H), 7.49 – 7.45 (m, 2H), 7.39 – 7.32 (m, 3H), 7.29 – 7.23 (m, 1H). ^{13}C NMR (101 MHz, $CDCl_3$) δ 166.4, 136.0, 134.5, 131.7, 128.7, 127.6, 126.2, 123.7, 120.2, 117.6.

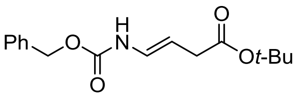

(*E*)-*tert*-butyl 4-(((benzyloxy)carbonyl)amino)but-3-enoate (32): Following the general procedure B, obtained in 68% yield as a colorless oil (39.6 mg, *E/Z* = 93/7, eluent: petroleum ether/ethyl acetate = 5/1).

^{1}H NMR (400 MHz, $CDCl_3$) δ 8.49 (d, *J* = 9.1 Hz, 1H), 7.51 – 7.23 (m, 5H), 6.57 (dd, *J* = 14.3, 10.4 Hz, 1H), 5.22 (dt, *J* = 14.6, 7.4 Hz, 1H), 5.12 (s, 2H), 2.93 (d, *J* = 7.3 Hz, 2H), 1.42 (s, 9H). ^{13}C NMR (101 MHz, $CDCl_3$) δ 171.7, 154.6, 137.9, 129.3, 128.9, 127.7,

103.6, 80.5, 67.1, 36.9, 28.3. (one carbon signal is overlapped) HRMS (ESI): $[M+Na]^+$ Calcd. for $C_{16}H_{21}NO_4Na$, 314.1363; found: 314.1352.

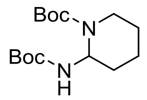

***tert*-butyl 2-((*tert*-butoxycarbonyl)amino)piperidine-1-carboxylate (33):** Following the general procedure B, obtained in 75% yield as a colorless liquid (45.0 mg, eluent: petroleum ether/ethyl acetate = 4/1).

^{1}H NMR (400 MHz, CD_2Cl_2) δ 5.81 – 5.67 (m, 1H), 5.16 (br s, 1H), 3.82 – 3.78 (m, 1H), 2.75 – 2.69 (m, 1H), 1.67 – 1.38 (m, 6H), 1.36 (s, 9H), 1.35 (s, 9H). ^{13}C NMR (101 MHz, CD_2Cl_2) δ 154.3, 79.5, 79.2, 58.7, 39.2, 30.0, 28.1, 28.0, 24.9, 18.8. (one carbon signal is overlapped) HRMS (ESI): $[M+Na]^+$ Calcd. for $C_{15}H_{28}N_2O_4Na$, 323.1941; found: 323.1931.

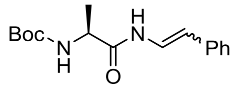

(*S*)-*tert*-butyl (1-oxo-1-(styrylamino)propan-2-yl)carbamate (34): Following the general procedure B, obtained in 88% yield as a crystalline solid (51.1 mg, *E/Z* = 82/18, eluent: petroleum ether/ethyl acetate = 3/1). The compound data was in agreement with the literature[20].

(*E*)-isomer

^{1}H NMR (400 MHz, $CDCl_3$) δ 9.00 – 8.80 (m, 1H), 7.44 (dd, *J* = 14.6, 10.6 Hz, 1H), 7.29 – 7.20 (m, 4H), 7.14 (t, *J* = 6.6 Hz, 1H), 6.15 (d, *J* = 14.7 Hz, 1H), 5.40 (d, *J* = 6.8 Hz, 1H), 4.46 – 4.26 (m, 1H), 1.47 (s, 9H), 1.42 (d, *J* = 7.1 Hz, 3H). ^{13}C NMR (101 MHz, $CDCl_3$) δ 170.6, 156.0, 136.1, 128.6, 126.6, 125.6, 122.5, 113.8, 80.6, 50.1, 28.3, 18.1.

(*Z*)-isomer

^{1}H NMR (400 MHz, $CDCl_3$) δ 8.72 – 8.44 (m, 1H), 7.38 (t, *J* = 7.6 Hz, 2H), 7.29 (d, *J* = 7.3 Hz, 2H), 7.24 (t, *J* = 7.3 Hz, 1H), 6.92 (dd, *J* = 11.4, 9.7 Hz, 1H), 5.77 (d, *J* = 9.6 Hz, 1H), 5.00 – 4.85 (m, 1H), 4.32 – 4.14 (m, 1H), 1.41 (s, 9H), 1.38 (d, *J* = 7.1 Hz, 3H). ^{13}C NMR (101 MHz, $CDCl_3$) δ 170.3, 155.7, 135.5, 129.0, 127.9, 126.9, 121.5, 110.7, 80.6, 50.2, 28.2, 17.4.

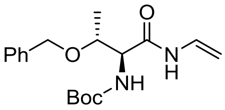

***tert*-butyl ((2*S*,3*R*)-3-(benzyloxy)-1-oxo-1-(vinylamino)butan-2-yl)carbamate (35):** Following the general procedure B, obtained in 62% yield as a colorless oil (41.5 mg, eluent: petroleum ether/ethyl acetate = 5/1).

^{1}H NMR (400 MHz, Acetone) δ 9.17 (d, *J* = 9.1 Hz, 1H), 7.36 – 7.23 (m, 5H), 6.96 (ddd, *J* = 16.0, 10.4, 9.0 Hz, 1H), 5.97 – 5.85 (m, 1H), 4.69 (d, *J* = 16.0 Hz, 1H), 4.61 (d, *J* = 11.8 Hz, 1H), 4.49 (d, *J* = 11.8 Hz, 1H), 4.37 (d, *J* = 8.9 Hz, 1H), 4.24 (dd, *J* = 8.8, 2.8 Hz, 1H), 4.14 – 4.07 (m, 1H), 1.42 (s, 9H), 1.20 (d, *J* = 6.3 Hz, 3H). ^{13}C NMR (101 MHz, Acetone) δ 168.3, 155.7, 138.7, 128.9, 128.2, 127.5, 127.4, 94.9, 78.8, 74.8, 70.7, 58.8, 27.6, 15.7. HRMS (ESI): $[M+Na]^+$ Calcd. for $C_{18}H_{26}N_2O_4Na$, 357.1785; found: 357.1780.

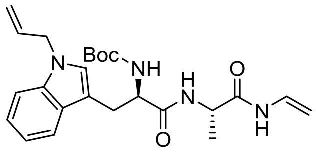

***tert*-butyl ((*R*)-3-(1-allyl-1*H*-indol-3-yl)-1-oxo-1-(((*S*)-1-oxo-1-(vinylamino)propan-2-yl)amino)propan-2-yl)carbamate (36):** Following the general procedure B, obtained in 42% yield as a yellow powder (37.0 mg, eluent: petroleum ether/ethyl acetate = 1/1).

^{1}H NMR (400 MHz, Acetone) δ 9.17 (d, *J* = 9.5 Hz, 1H), 7.62 (d, *J* = 7.9 Hz, 1H), 7.54 (d, *J* = 7.3 Hz, 1H), 7.36 (d, *J* = 8.2 Hz, 1H), 7.19 – 7.08 (m, 2H), 7.04 (t, *J* = 7.2 Hz, 1H), 6.88 (ddd, *J* = 16.0, 10.3, 8.9 Hz, 1H), 6.32 (d, *J* = 5.5 Hz, 1H), 6.06 – 5.98 (m, 1H), 5.14 (dd, *J* = 10.3, 1.4 Hz, 1H), 5.06 (dd, *J* = 17.1, 1.5 Hz, 1H), 4.85 – 4.75 (m, 3H), 4.42 – 4.36 (m, 1H), 4.34 (d, *J* = 8.9 Hz, 1H), 4.28 – 4.20 (m, 1H), 3.25 (dd, *J* = 14.3, 7.1 Hz, 1H), 3.15 (dd, *J* = 14.3, 7.4 Hz, 1H), 1.39 (s, 9H), 1.12 (d, *J* = 7.2 Hz, 3H). ^{13}C NMR (101 MHz, Acetone) δ 171.9, 169.9, 156.0, 136.5, 134.3, 129.2, 128.4, 127.2, 121.3, 118.8, 118.7, 116.1, 109.9, 109.7, 94.9, 78.8, 56.3, 48.5, 48.2, 27.7, 27.2, 16.7. HRMS (ESI): $[M+Na]^+$ Calcd. for $C_{24}H_{32}N_4O_4Na$, 463.2316; found: 463.2321.

Procedure for the Synthesis of Chondriamides A and C

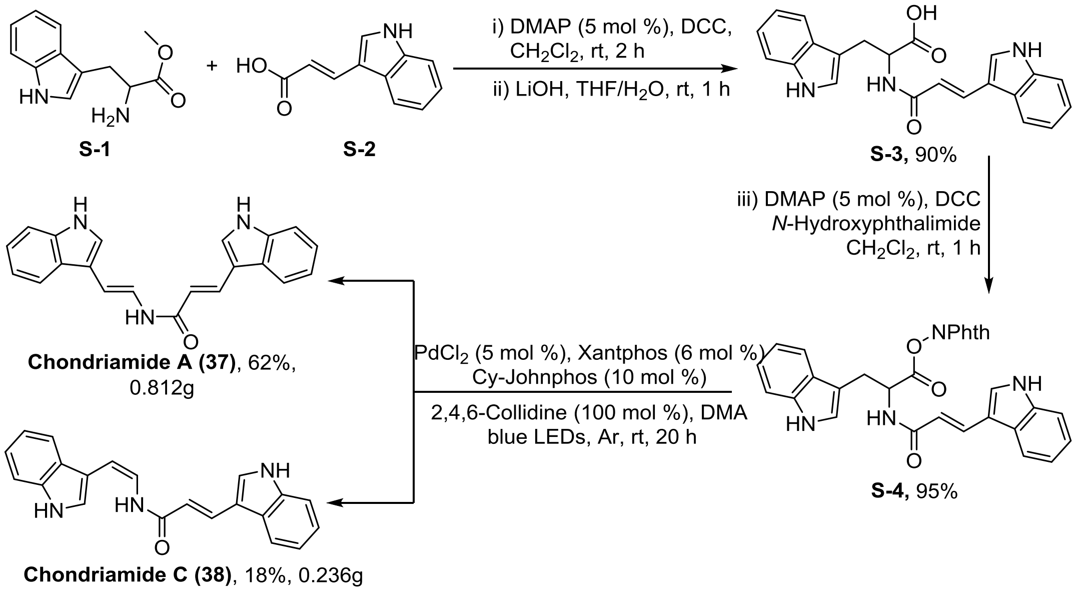

Procedure: The methyl tryptophan (**S-1**, 5 mmol, 1.091 g), 3-indoleacrylic acid (**S-2**, 5 mmol, 0.936 g), and 4-dimethylaminopyridine (DMAP, 5 mol %, 30.5 mg) were mixed in a flask with a magnetic stirring bar. 30 mL dry CH_2Cl_2 was added, then a solution of *N,N*′-dicyclohexylcarbodiimide (DCC, 5 mmol, 1.031 g) in CH_2Cl_2 (10 mL) was added slowly at room temperature. The reaction mixture was stirred at room temperature for 2 h. Then the white precipitate was filtered off and the solution was concentrated under vacuum. Corresponding amide was purified by column chromatography on silica gel. To a solution of the amide in distilled THF (15 mL) and distilled water (15 mL) at room temperature was added lithium hydroxide monohydrate (8 mmol, 336 mg). The resulting solution was stirred for 1 h at room temperature. The pH of the aqueous layer was adjusted to 2 with 1 N HCl solution. The mixture was extracted with EtOAc, dried over Na_2SO_4, filtered, and concentrated in vacuo to afford acid **S-3** (90%, 1.680 g). The compound **S-3** was added to an oven-dried flask with a magnetic stirring bar, then *N*-hydroxyphthalimide (4.5 mmol, 734 mg), 4-dimethylaminopyridine (5 mol %, 27.5 mg) and 20 mL dry CH_2Cl_2 was added. A solution of *N,N*′-dicyclohexylcarbodiimide (DCC, 4.5 mmol, 0.928 g) in CH_2Cl_2 (10 mL) was added to the mixture slowly at room temperature. The resulting solution was stirred at room temperature for 1 h. After *N*-hydroxyphthalimide was completely converted, the white precipitate was filtered off and the solution was concentrated under vacuum. The crude mixture was purified by column chromatography on silica gel to give the redox active ester **S-4** (2.216 g, 95%).

The redox active ester **S-4** (4 mmol, 2.074g), $PdCl_2$ (5 mol %, 35.5 mg), Xantphos (6 mol %, 138.9 mg) and Cy-Johnphos (10 mol %, 140.2 mg) were placed in a transparent Schlenk tube equipped with a stirring bar. The tube was evacuated and filled with argon (three times). To these solids, anhydrous *N*,*N*-dimethylacetamide (DMA, 20 mL) and 2,4,6-collidine (100 mol %, 484.7 mg) was added via a gastight syringe under argon atmosphere. The reaction mixture was stirred under the irradiation of blue LEDs (distance app. 3.0 cm from the bulb) at room temperature for 20 h. After 20 h, the mixture was quenched with saturated NaCl solution and extracted with ethyl acetate (3 x 40 mL). The organic layers were combined and concentrated under vacuo. The products was purified by flash column chromatography on silica gel (eluent: petroleum ether/ethyl acetate = 1/2).

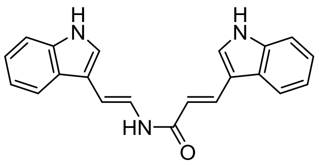

Chondriamide A (37): Following the above procedure, obtained in 62% yield as a yellow powder (812 mg). The compound data was in agreement with the literature[21].
^{1}H NMR (400 MHz, Acetone) δ 10.77 (br s, 1H), 10.26 (br s, 1H), 9.33 (d, *J* = 10.6 Hz, 1H), 7.98 (d, *J* = 7.2 Hz, 1H), 7.91 (d, *J* = 15.6 Hz, 1H), 7.82 – 7.77 (m, 2H), 7.73 (dd, *J* = 14.9, 10.5 Hz, 1H), 7.54 – 7.50 (m, 1H), 7.46 – 7.39 (m, 2H), 7.26 – 7.17 (m, 2H), 7.17 – 7.09 (m, 2H), 6.80 (d, *J* = 15.6 Hz, 1H), 6.47 (d, *J* = 14.9 Hz, 1H). ^{13}C NMR (101 MHz, Acetone) δ 163.5, 137.9, 137.3, 134.7, 130.1, 125.5, 125.4, 122.6, 122.5, 121.7, 121.0, 120.6, 120.2, 119.3, 115.6, 113.2, 113.0, 112.2, 111.6, 104.9. (one carbon signal is overlapped) HRMS (ESI): $[M+Na]^+$ Calcd. for $C_{21}H_{17}N_3ONa$, 350.1264; found: 350.1253.

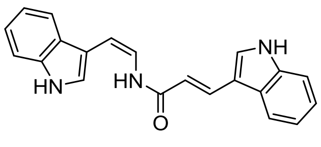

Chondriamide C (38): Following the above procedure, obtained in 18% yield as a yellow powder (236 mg). The compound data was in agreement with the literature[21].

^{1}H NMR (400 MHz, Acetone) δ 10.81 (br s, 1H), 10.46 (br s, 1H), 8.77 (d, *J* = 10.7 Hz, 1H), 7.98 (d, *J* = 7.9 Hz, 1H), 7.93 (d, *J* = 15.6 Hz, 1H), 7.77 (d, *J* = 2.7 Hz, 1H), 7.67 – 7.62 (m, 2H), 7.53 – 7.48 (m, 1H), 7.44 (dd, *J* = 8.0, 0.8 Hz, 1H), 7.24 – 7.06 (m, 5H), 6.97 (d, *J* = 15.6 Hz, 1H), 5.94 (d, *J* = 9.5 Hz, 1H). ^{13}C NMR (101 MHz, Acetone) δ 164.3, 137.9, 136.2, 135.4, 130.5, 127.1, 125.3, 123.3, 122.5, 121.9, 120.6, 120.5, 120.4, 119.3, 118.7, 115.4, 113.3, 112.1, 111.3, 110.9, 100.9. HRMS (ESI): $[M+Na]^+$ Calcd. for $C_{21}H_{17}N_3ONa$, 350.1264; found: 350.1258.

Supplementary Discussion

Study the Reaction Selectivity of S-5

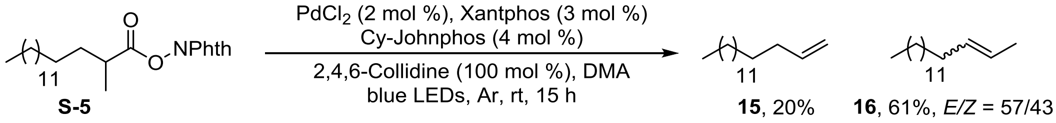

Following the general procedure A, the **S-5** was used as substrate, the terminal olefin and internal olefin can be obtained in 81% yield (36.3 mg, eluent: petroleum ether). The ratio of products was determined by ^{1}H NMR analysis (Supplementary Figure 25).

^{1}H NMR (400 MHz, $CDCl_3$) δ 5.81 (ddt, *J* = 16.9, 10.2, 6.7 Hz, 1H, terminal olefin), 5.46 – 5.34 (m, 6.09H, internal olefins), 5.03 – 4.90 (m, 2H, terminal olefin), 2.08 – 1.92 (m, 8.11H), 1.66 – 1.63 (m, 5.21H, *E*-isomer), 1.60 (d, *J* = 6.0 Hz, 3.94H, *Z*-isomer), 1.32 – 1.23 (m, 91.15H), 0.90 – 0.86 (m, 12.18H).

Study the Reaction Selectivity of S-6

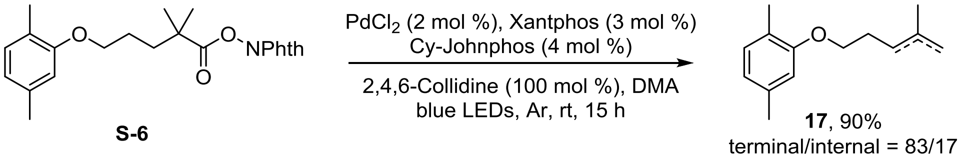

Following the general procedure A, the **S-6** was used as substrate, the terminal olefin and internal olefin can be obtained in 90% yield as colorless liquid (36.8 mg, eluent: petroleum ether/ethyl acetate = 100/1). The ratio of products was determined by ^{1}H NMR analysis (Supplementary Figure 26).

^{1}H NMR (400 MHz, $CDCl_3$) δ 7.00 (d, *J* = 7.4 Hz, 1H), 6.73 – 6.57 (m, 2H), 5.23 (t, *J* = 7.2 Hz, 0.17H, internal olefins), 4.76 – 4.72 (m, 1.65H, terminal olefin), 4.03 – 3.87 (m, 2H), 2.53 – 2.45 (m, 0.34H), 2.31 (s, 3H), 2.24 – 2.16 (m, 4.66H), 1.98 – 1.90 (m, 1.66H), 1.76 (s, 2.49H), 1.73 (s, 0.51H), 1.67 (s, 0.51H).

Study the Reaction Selectivity of S-7

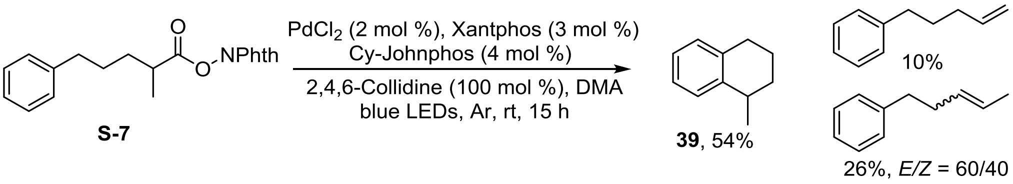

Following the general procedure A, the **S-7** was used as substrate, the olefins and

cyclization product can be obtained in 90% yield as colorless liquid (26.3 mg, eluent: petroleum ether). The ratio of products was determined by ^{1}H NMR analysis (Supplementary Figure 79).

^{1}H NMR (400 MHz, $CDCl_3$) δ 7.31 – 7.25 (m, 0.80H), 7.22 – 7.03 (m, 3.60H), 5.83 (ddt, *J* = 16.9, 10.2, 6.6 Hz, 0.11H, terminal olefin), 5.54 – 5.38 (m, 0.58H, internal olefins), 5.08 – 4.94 (m, 0.22H, terminal olefin), 2.96 – 2.85 (m, 0.60H, cyclization product), 2.82 – 2.70 (m, 1.20H), 2.69 – 2.59 (m, 0.80H), 2.42 – 2.25 (m, 0.58H), 2.13 – 2.05 (m, 0.22H), 1.98 – 1.81 (m, 1.20H), 1.78 – 1.67 (m, 0.80H), 1.64 (dt, *J* = 4.6, 1.2 Hz, 0.52H, *E*-isomer), 1.58 – 1.51 (m, 0.95H), 1.29 (d, *J* = 7.0 Hz, 1.80H).

1-methyl-1,2,3,4-tetrahydronaphthalene (39): The compound data was in agreement with the literature[22].

^{1}H NMR (400 MHz, $CDCl_3$) δ 7.22 – 7.03 (m, 4H), 2.96 – 2.85 (m, 1H), 2.81 – 2.73 (m, 2H), 1.97 – 1.82 (m, 2H), 1.76 – 1.69 (m, 1H), 1.57 – 1.52 (m, 1H), 1.29 (d, *J* = 7.0 Hz, 3H). ^{13}C NMR (101 MHz, $CDCl_3$) δ 142.2, 136.9, 129.0, 128.1, 125.6, 125.4, 32.5, 31.5, 30.0, 22.9, 20.4.

Radical Experiments

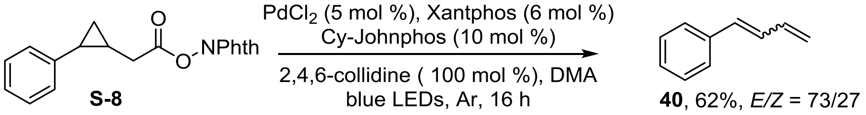

The **40** was prepared according to the general procedure B in 62% yield with **S-8** as substrate. The ratio of products was determined by ^{1}H NMR analysis (Supplementary Figure 77).

^{1}H NMR (400 MHz, $CDCl_3$) δ 7.44 – 7.15 (m, 5H), 6.96 – 6.73 (m, 1H), 6.61 – 6.40 (m, 1.73H), 6.25 (t, *J* = 11.3 Hz, 0.27H, *Z*-isomer), 5.44 – 5.27 (m, 1H), 5.26 – 5.11 (m, 1H).

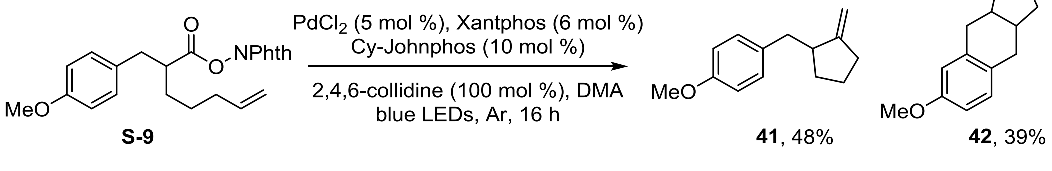

Using **S-9** as a reaction substrate, cyclization products **41** and **42** were obtained in 48% yield and 39% yield, respectively, without the formation of direct elimination product.

The ratio of products **41** and **42** was determined by ^{1}H NMR analysis (Supplementary Figure 78).

^{1}H NMR (400 MHz, $CDCl_3$) δ 7.10 (d, J = 8.5 Hz, 2H, **41**), 7.00 (d, J = 8.0 Hz, 0.82H, **42**), 6.82 (d, J = 8.6 Hz, 2H, **41**), 6.72 – 6.60 (m, 1.63H, **42**), 4.91 (s, 1H, **41**), 4.82 (s, 1H, **41**), 3.78 (s, 5.48H, **41+42**), 2.89 (dd, J = 13.6, 5.1 Hz, 1H, **41**), 2.75 – 2.54 (m, 2.63H, **41+42**), 2.45 – 2.26 (m, 4.66H, **41+42**), 1.83 – 1.13 (m, 8.92H, **41+42**).

Photophysical Studies

To understand the role of palladium catalyst, a series of photophysical studies was conducted. First, the UV-Vis spectrum of reaction mixture was measured, suggesting that palladium catalyst was the light-absorbing species between 400-500 nm and the redox-active ester did not absorb light at this region (Supplementary Figure 80). Then, the Stern-Volmer studies were conducted by irradiating $Pd(PPh_3)_4$ (1.0×10^{-4} M in DMA) at 440 nm, where the blue LED has the maximum emission, to show emission peak at 620 nm. The emission band intensity was decreased by increasing concentration of palmitic acid-derived redox-active ester, indicating that the redox-active ester quenches excited Pd(0) species. The linear correlation for Stern-Volmer plot was obtained between Io/I and the concentration of redox-active ester (Supplementary Figure 81).

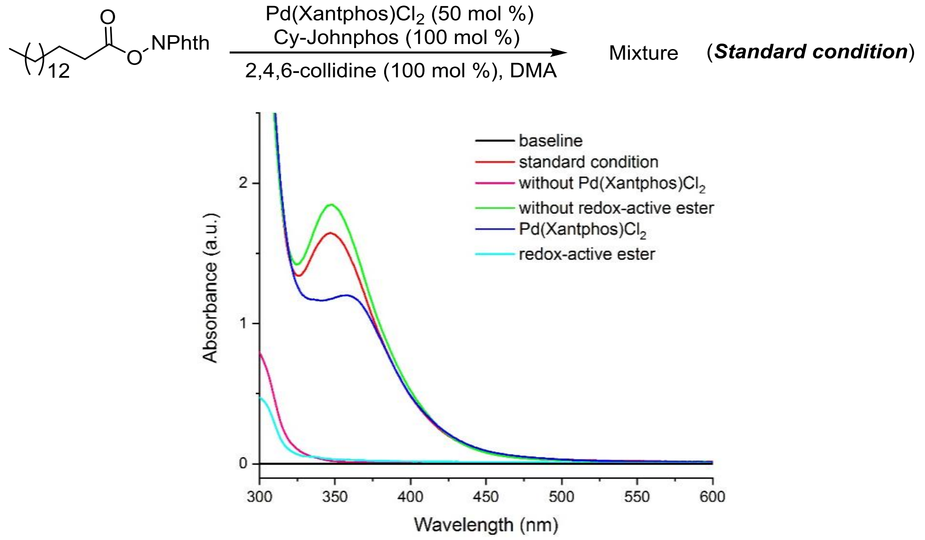

Supplementary Figure 80. UV-Vis spectrum of reaction mixture. $Pd(Xantphos)Cl_2$ (1.25×10^{-4} M), Cy-Johnphos (2.50×10^{-4} M), redox-active ester (2.50×10^{-4} M) and collidine (2.50×10^{-4} M) in DMA.

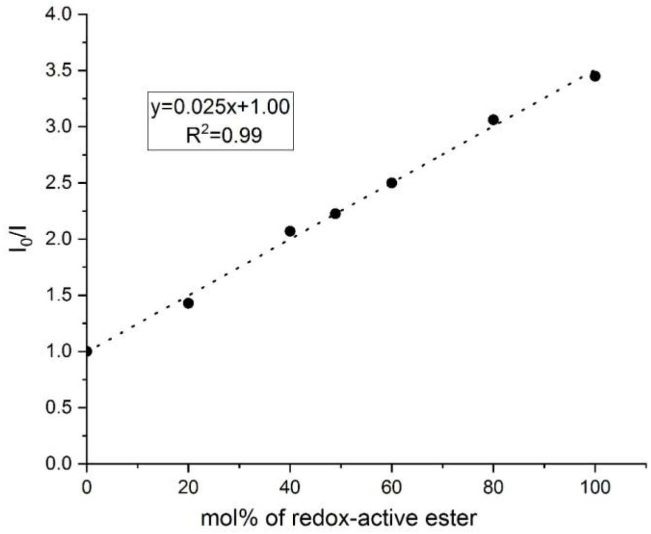

Supplementary Figure 81. Stern-Volmer studies. Stern-Volmer plot for the emission quenching of $Pd(PPh_3)_4$ by various concentrations of redox-active ester (from 0 to 100 mol% with respect to $Pd(PPh_3)_4$ in DMA)

X-band EPR Spectra

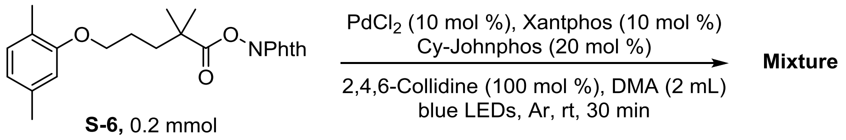

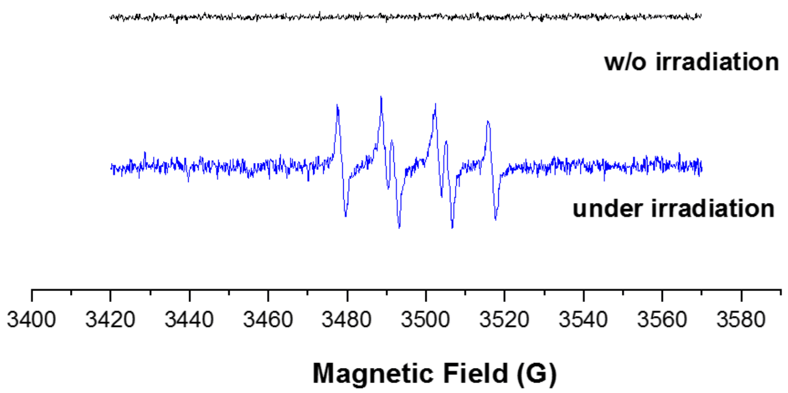

Supplementary Figure 82. X-band EPR spectra. Electron paramagnetic resonance (EPR) studies of reaction mixtures with and without (w/o) irradiation.

Black line: redox active ester (**S-6**, 0.2 mmol), $PdCl_2$ (10 mol %), Xantphos (10 mol %), Cy-Johnphos (20 mol %) were placed in a transparent Schlenk tube equipped with a

stirring bar. The tube was evacuated and filled with argon (three times). To these solids, anhydrous DMA (2 mL) and 2,4,6-collidine (100 mol %, 0.2 mmol) were added via a gastight syringe under argon atmosphere. The reaction mixture was stirred in the dark for 30 minutes at room temperature and then transferred via syringe (20 μL) under nitrogen to an EPR tube *(with 1 μL DMPO inside for tapping the alkyl radical)* for X-band EPR measurement at 298 K.

Blue line: redox active ester (**S-6**, 0.2 mmol), $PdCl_2$ (10 mol %), Xantphos (10 mol %), Cy-Johnphos (20 mol %) were placed in a transparent Schlenk tube equipped with a stirring bar. The tube was evacuated and filled with argon (three times). To these solids, anhydrous DMA (2 mL) and 2,4,6-collidine (100 mol %, 0.2 mmol) were added via a gastight syringe under argon atmosphere. The reaction mixture was stirred under the irradiation of Blue LEDs for 30 minutes at room temperature and then transferred via syringe (20 μL) under nitrogen to an EPR tube *(with 1 μL DMPO inside for tapping the alkyl radical)* for X-band EPR measurement at 298 K.

Supplementary References

1. Schwarz, J. & König, B. Metal-free, visible-light-mediated, decarboxylative alkylation of biomass-derived compounds. *Green Chem.* **18**, 4743−4749 (2016).
2. Ouairy, C. M. J., Ferraz, M. J., Boot, R. G., Baggelaar, M. P., van der Stelt, M., Appelman, M., van der Marel, G. A., Florea, B. I., Aerts, J. M. F. G. & Overkleeft, H. S. Development of an acid ceramidase activity-based probe. *Chem. Commun.* **51**, 6161−6163 (2015).
3. John, A., Dereli, B., Ortuño, M. A., Johnson, H. E., Hillmyer, M. A., Cramer, C. J. & Tolman, W. B. Selective decarbonylation of fatty acid esters to linear α-olefins. *Organometallics* **36**, 2956−2964 (2017).
4. Akram, M. O., Mali, P. S. & Patil, N. T. Cross-coupling reactions of aryldiazonium salts with allylsilanes under merged gold/visible-light photoredox catalysis. *Org. Lett.* **19**, 3075−3078 (2017).
5. Xi, X., Chen, T., Zhang, J.-S. & Han, L.-B. Efficient and selective hydrogenation of C–O bonds with a simple sodium formate catalyzed by nickel. *Chem. Commun.* **54**, 1521−1524 (2018).
6. Schmid, T. E., Bantreil, X., Citadelle, C. A., Slawin, A. M. Z. & Cazin, C. S. J. Phosphites as ligands in ruthenium-benzylidene catalysts for olefin metathesis. *Chem. Commun.* **47**, 7060−7062 (2011).
7. Ando, K., Kobayashi, T. & Uchida, N. Practical methylenation reaction for aldehydes and ketones using new Julia-type reagents. *Org. Lett.* **17**, 2554–2557 (2015).
8. De Simone, F., Gertsch, J. & Waser, J. Catalytic selective cyclizations of aminocyclopropanes: formal synthesis of aspidospermidine and total synthesis of goniomitine. *Angew. Chem. Int. Ed.* **49**, 5767–5770 (2010).
9. Butcher, T. W., McClain, E. J., Hamilton, T. G., Perrone, T. M., Kroner, K. M., Donohoe, G. C., Akhmedov, N. G., Petersen, J. L. & Popp, B. V. Regioselective copper-catalyzed boracarboxylation of vinyl arenes. *Org. Lett.* **18**, 6428–6431 (2016).
10. Wang, X., Chen, R.-X., Wei, Z.-F., Zhang, C.-Y., Tu, H.-Y. & Zhang, A.-D. Chemoselective transformation of diarylethanones to arylmethanoic acids and diarylmethanones and mechanistic insights. *J. Org. Chem.* **81**, 238–249 (2016).
11. Chen, W., Tao, H., Huang, W., Wang, G., Li, S., Cheng, X. & Li, G. Hantzsch ester as a photosensitizer for the visible-light-induced debromination of vicinal dibromo

compounds. *Chem. Eur. J.* **22**, 9546–9550 (2016).

12. Greenhalgh, M. D. & Thomas, S. P. Iron-catalyzed, highly regioselective synthesis of α-aryl carboxylic acids from styrene derivatives and CO_2. *J. Am. Chem. Soc.* **134**, 11900–11903 (2012).
13. Fang, X., Cacherat, B. & Morandi, B. CO- and HCl-free synthesis of acid chlorides from unsaturated hydrocarbons via shuttle catalysis. *Nat. Chem.* **9**, 1105–1109 (2017).
14. Luo, S.-X., Cannon, J. S., Taylor, B. L. H., Engle, K. M., Houk, K. N. & Grubbs, R. H. *Z*-selective cross-metathesis and homodimerization of 3E-1,3-dienes: reaction optimization, computational analysis, and synthetic applications. *J. Am. Chem. Soc.* **138**, 14039–14046 (2016).
15. Wang, Q., Khoury, M. E. & Schlosser, M. Bridging the final gap in stereocontrolled Wittig reactions: methoxymethoxy-armed allylic phosphorus ylides affording conjugated dienes with high *cis* selectivity. *Chem. Eur. J.* **6**, 420–426 (2000).
16. Dupuy, S. & Nolan, S. P. Gold(I)-catalyzed protodecarboxylation of (hetero)aromatic carboxylic acids. *Chem. Eur. J.* **19**, 14034−14038 (2013).
17. Wegner, J., Ley, S. V., Kirschning, A., Hansen, A. L., Garcia, J. M. & Baxendale, I. R. A total synthesis of millingtonine A. *Org. Lett.* **14**, 696–699 (2012).
18. Carboni, A., Dagousset, G., Magnier, E. & Masson, G. Photoredox-induced three-component oxy-, amino-, and carbotrifluoromethylation of enecarbamates. *Org. Lett.* **16**, 1240–1243 (2014).
19. García-Reynaga, P., Carrillo, A. K. & VanNieuwenhze, M. S. Decarbonylative approach to the synthesis of enamides from amino acids: stereoselective synthesis of the (*Z*)-aminovinyl-d-cysteine unit of mersacidin. *Org. Lett.* **14**, 1030–1033 (2012).
20. Min, G. K., Hernández, D., Lindhardt, A. T. & Skrydstrup, T. Enamides accessed from aminothioesters via a Pd(0)-catalyzed decarbonylative/β-hydride elimination sequence. *Org. Lett.* **12**, 4716–4719 (2010).
21. Wang, X. & Porco, J. A. Modification of C-terminal peptides to form peptide enamides: synthesis of chondriamides A and C. *J. Org. Chem.* **66**, 8215−8221 (2001).
22. Kato, S., Saga, Y., Kojima, M., Fuse, H., Matsunaga, S., Fukatsu, A., Kondo, M., Masaoka, S. & Kanai, M. Hybrid catalysis enabling room-temperature hydrogen gas release from *N*-heterocycles and tetrahydronaphthalenes. *J. Am. Chem. Soc.* **139**, 2204−2207 (2017).
